# Theory of Condensate Size Control by Molecular Charge Asymmetry

**DOI:** 10.1021/acsmacrolett.5c00342

**Published:** 2025-09-29

**Authors:** Chengjie Luo, Nathaniel Hess, Dilimulati Aierken, Yicheng Qiang, Jerelle A. Joseph, David Zwicker

**Affiliations:** † 28283 Max Planck Institute for Dynamics and Self-Organization, Am Faßberg 17, 37077 Göttingen, Germany; ‡ Department of Chemical and Biological Engineering, 6740 Princeton University, Princeton, New Jersey 08544, United States; § Omenn−Darling Bioengineering Institute, 6740 Princeton University, Princeton, New Jersey 08544, United States

## Abstract

Biomolecular condensates are complex droplets comprising diverse molecules that interact by various mechanisms. Condensation is often driven by short-range attraction, but net charges can also mediate long-range repulsion. Using molecular dynamics simulations and an equilibrium field theory, we show that such opposing interactions can suppress coarsening, so many droplets of equal size coexist at equilibrium. This size control depends strongly on the charge asymmetry between molecular constituents, while the strength of the short-range attractions has a weak influence. The mechanism relies on droplets expelling ions; therefore, they cannot screen electrostatics effectively, implying that droplets acquire a net charge and cannot grow indefinitely. Our simulations indicate that this effect is likely less prevalent in biomolecular condensates within cells, although we still observe stable small clusters in this case. Taken together, our work reveals that electrostatic effects through molecular charge asymmetries can control droplet size, which contributes to our understanding of biomolecular condensates and the creation of synthetic patterns in chemical engineering.

## Graphical abstract

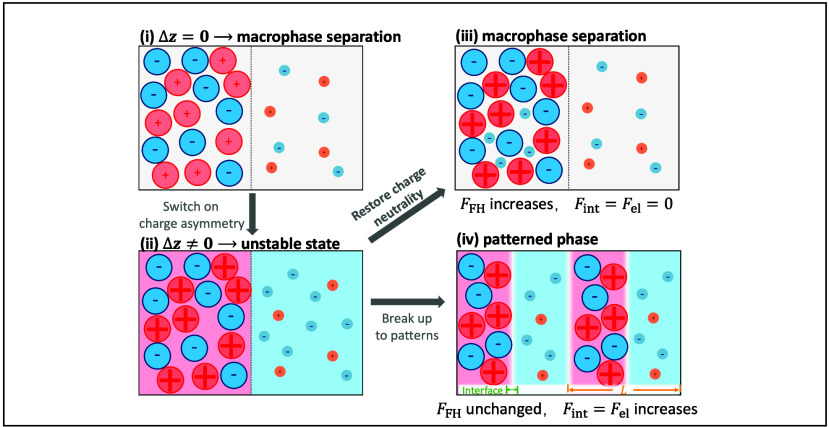

Biomolecular condensates are complex droplets that are key for numerous cellular functions.^1^ They typically comprise diverse biomolecules, including nucleic acids and proteins, and form by phase separation due to various short-range interactions. On microscopic length scales, typical interactions include π–π-stacking, cation−π interactions, hydrogen bonding, and hydrophobic interactions.^2,3^ Moreover, many of the involved molecules are charged to different degrees, and their charges can be dynamically modified (e.g., by post-translational modifications),^3^ implying electrostatic interactions. These electrostatic interactions can contribute to the short-range interactions, leading to phase separation. An example are complex coacervates, where oppositely charged polyions attract each other and phase separate from a solvent.^4–8^ Electrostatics also induce interactions between regions that are large compared with molecules if these regions possess a net charge. However, such long-range interactions are typically screened by mobile salt ions that neutralize charges on macromolecules in dilute electrolytes like the cytosol,^9^ although such screening might be suppressed if phase separation affects the distribution of ions. Taken together, many types of short-range interactions are crucial for forming biomolecular condensates, but the influence of long-range electrostatic effects is unclear.

Previous theoretical work suggests that long-range electrostatic effects are relevant in phase separating systems. For instance, a net charge of droplets affects their interfaces,^10,11^ reduces coarsening,^12–14^ and can potentially lead to fission.^15,16^ In particular, reduced surface tension^17^ and an energy barrier suppressing coalescence^13^ slow down coarsening. Simulations also find that heterogeneous charges can lead to multiphasic coacervates,^18^ and while polyelectrolytes with more charges phase separate more strongly,^19^ a large charge asymmetry can suppress phase separation completely.^17^ Polyelectrolytes can also form regular patterns, often called microphase separation,^20,21^ when the electrostatic interactions that drive phase separation are counterbalanced by either short-range interactions^22^ or surface tension.^23^ These clusters are stabilized by a nonzero net charge resulting from an unequal number of polycations and polyanions.^20^ These observations indicate that net charge fundamentally affects the phase separation of macromolecules. However, it is unclear whether the same effects occur in weakly charged biopolymers that phase separate due to specific short-range attraction in a salt-rich environment.

To investigate the phase separation of weakly charged macromolecules in the presence of ions, we first performed molecular dynamics (MD) simulations of a system consisting of positively charged polymers *P* ^+^, negatively charged polymers *P* ^–^, and related ions, *e* ^+^ and *e* ^–^, in the *NVT* ensemble with an implicit solvent modeled using a Langevin thermostat (Figure 1a). We induced phase separation using an attractive Lennard-Jones interaction between *P* ^+^ and *P* ^–^, while all other species exhibit excluded volume interactions. For simplicity, we kept the length of the polymers fixed at ten times the size of an ion and initialized the system such that *P* ^+^ and *P* ^–^ each occupy 10% of the volume, expecting the formation of droplets enriched in *P* ^+^ and *P* ^–^. Electrostatic interactions are given by Coulomb potentials between all species, which have charge numbers of *z*_*i*_. In particular, we varied the respective charge numbers *z*_*P* ^+^_ > 0 and *z*_*P* ^–^_ < 0 per monomer, whereas the ions have *z*_*e* ^+^_ = 1 and *z*_*e* ^–^_ = −1. To have comparable electrostatic effects, we added as many positive ions *e* ^+^ as there are charges on the negatively charged polymers *P* ^–^, and vice versa for *e* ^–^. Consequently, the overall system is neutral, and excess ions play the role of salt, which can screen electrostatic effects between polymers. Additional simulation details are described in the Supporting Information. The snapshots of equilibrated MD simulations shown in Figure 1b demonstrate that polymers form dense droplets surrounded by a dilute region. Importantly, droplets are smaller for larger charge asymmetry Δ*z* = *z*_*P* ^+^_ + *z*_*P* ^–^_, indicating that charges can control the droplet size and multiple droplets coexist stably.

**Figure 1:**
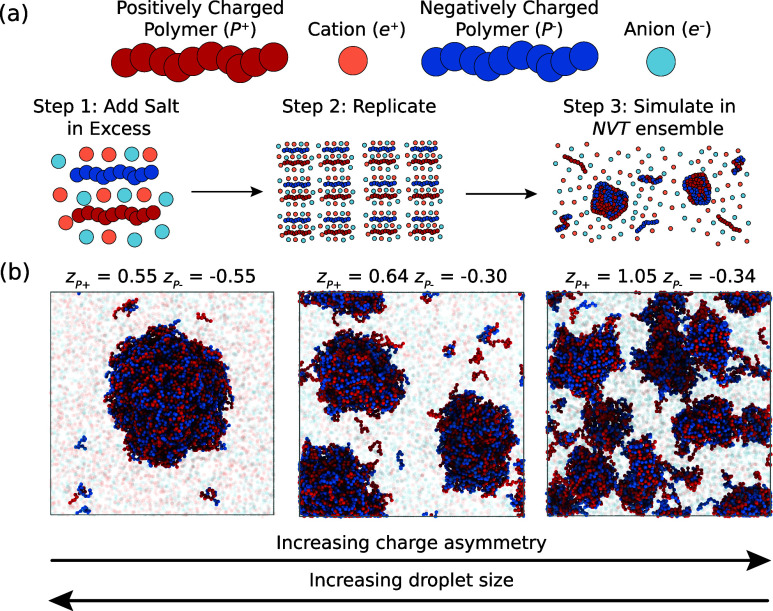
MD simulations show smaller droplets for larger charge asymmetry. (a) Schematic of simulations setup, where a system of two oppositely charged polymers is neutralized with an excess of ions, replicated, and simulated in the *NVT* ensemble (constant number of particles, volume, and temperature). (b) Snapshots from three simulations at various charges of the positively charged (*z*_*P* ^+^_, red) and negatively charged polymers (*z*_*P* ^–^_, blue) showing that larger charge asymmetry leads to smaller droplets. For clarity, ions are shown to be 99% transparent.

To reveal the physical mechanism of the observed droplet size regulation, we next describe the system using continuous field theory. Here, the state of the incompressible, isothermal mixture is captured by the volume fraction fields ϕ_*P* ^+^_(**r**), ϕ_*P* ^–^_(**r**), ϕ_*e* ^+^_(**r**), and ϕ_*e* ^–^_(**r**) of the charged species. The remaining fraction, ϕ_*S*_ = 1 – ∑_*i*_ ϕ_*i*_ (here and below sums are over the four species *P* ^+^, *P* ^–^, *e* ^+^, and *e* ^–^), is filled by an inert solvent *S*. The system’s equilibrium is governed by the minimum of the total free energy *F* = *F*_FH_ + *F*_int_ + *F*_el_, where the terms, respectively, capture local, interfacial, and long-range electrostatic interactions akin to ref 11. We approximate local interactions using the Flory–Huggins model^24,25^

1 $$F_{\mathrm{FH}}=\frac{k_{B}T}{v}\int \left[\chi \phi_{P^{-}}\phi_{P^{+}}+\phi_{S}\ln\phi_{S}+\underset{i}{\sum }\frac{\phi_{i}}{\nu_{i}}\ln\phi_{i}\right]dV$$

where integrals are over the system of volume *V*; *k*_B_ *T* is the relevant thermal energy scale; and *v* denotes the molecular volume of solvent molecules, while all other species have molecular volumes ν_*i*_ *v*. In the integrand, the first term proportional to the Flory parameter χ < 0 captures the short-range attraction between *P* ^+^ and *P* ^–^, whereas the other terms capture translational entropies. To mimic the MD simulations, we chose ν_*e* ^+^_ = ν_*e* ^–^_ = 1 and ν_*P* ^+^_ = ν_*P* ^–^_ = 10. The short-range interaction also implies an interfacial energy, which we describe by a simple gradient term^26^

$$F_{\mathrm{int}}=\frac{\kappa k_{B}T}{2v}\int \underset{i}{\sum }|\nabla \phi_{i}{|}^{2}dV$$ 2

which limits the width of interfaces between coexisting phases to roughly $\sqrt{\kappa }$ in strongly interacting systems.^27^ We chose a constant coefficient κ to simplify the interpretation, and we will later check whether more realistic models alter the behavior. Finally, long-range electrostatic effects are captured by the electrostatic potential ψ, which is governed by the free energy

$$F_{\mathrm{el}}=\int \left[-\frac{\varepsilon }{8\pi }|\nabla \psi {|}^{2}+\frac{e\psi }{v}\underset{i}{\sum }z_{i}\phi_{i}\right]dV$$ 3

where *ε* is the dielectric constant, assumed to be constant in space, and *z*_*i*_ is the number of charges per unit volume *v* for species *i*. Taken together, the equilibrium state thus depends on the local attraction (χ), the interfacial penalty (κ), and the charge numbers (*z*_*P* ^+^_ and *z*_*P* ^–^_).

As in the MD simulations, we impose constant average fractions ϕ̅_*i*_ = *V* ^–1^ ∫ ϕ_*i*_d*V* with ϕ̅_*P* ^+^_ = ϕ̅_*P* ^–^_ = 0.1, ϕ̅_*e* ^–^_ = *z*_*P* ^+^_ϕ̅_*P* ^+^_, and ϕ̅_*e* ^+^_ = – *z*_*P* ^–^_ϕ̅_*P* ^–^_. Based on the results shown in Figure 1b, we expect that equilibrium states exhibit droplets of a well-defined size, which correspond to periodic profiles in field theory. To describe such states, we consider a one-dimensional, periodic system of variable length *L*, and minimize *F* by varying *L*, ψ­(*x*), and ϕ_*i*_(*x*). Here, we allow for coexisting, thermodynamically large phases, each of which can exhibit periodic concentration profiles. We impose mass conservation, incompressibility, and charge neutrality within each phase using Lagrange multipliers, akin to ref 28 and described in the Supporting Information. The resulting equilibrium profiles shown in Figure 2 indicate that the field theory indeed predicts periodic patterns, where regions of large polymer density correspond to the droplets, and the surrounding region enriched in ions represents the dilute region. Note that the ions do not neutralize the system everywhere (black line), which we confirmed in MD simulations (Supporting Figure S1). These net charges are reminiscent of the double layer structure of complex coacervates^29^ and indicate that electrostatics are crucial for droplet size control.^23,30,31^ Indeed, we observe larger periods *L* for smaller charge asymmetry (Figure 2), consistent with the larger droplets in our MD simulations (Figure 1b). There are also subtle differences between the two panels: For small charge asymmetry (Figure 2a), the charge density ρ exhibits a dip inside the droplet and reaches neutrality (ρ = 0) in the dilute region. Both effects are absent for larger charge asymmetry (Figure 2b), suggesting that these two cases are qualitatively different.

**Figure 2:**
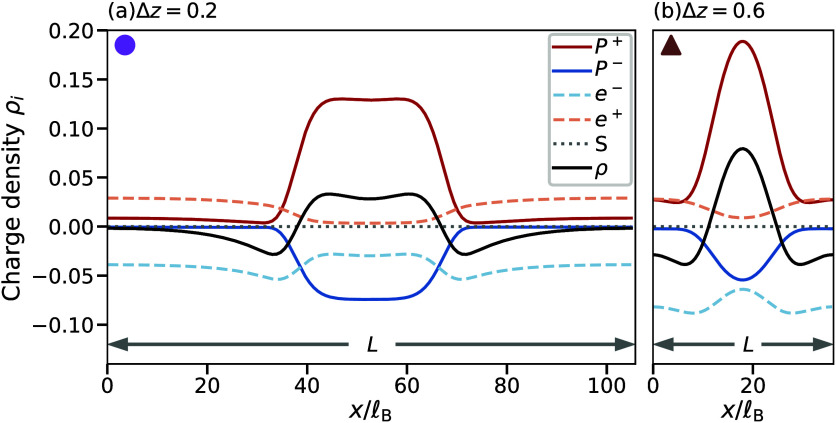
Field theory predicts droplet-like periodic patterns. Periodic profiles of charge density ρ_*i*_ = *z*_*i*_ϕ_*i*_ for all species *i* and total charge density ρ = ∑_*i*_ ρ_*i*_ as a function of position *x* for weak (left) and strong (right) charge asymmetry Δ*z* = *z*_*P* ^+^_ + *z*_*P* ^–^_. Parameters are *z*_*P* ^–^_ = −0.2, $v=100l_{B}^{3}$ , ν_*P* ^+^_ = ν_*P* ^–^_ = 10, ν_*e* ^+^_ = ν_*e* ^–^_ = 1, ϕ̅_*P* ^+^_ = ϕ̅_*P* ^–^_ = 0.1, $\kappa =10l_{B}^{2}$ , and χ = – 5, with Bjerrum length $l_{B}=e^{2}/\left(\varepsilon k_{B}T\right)$ .

We next analyze in detail how the periodic patterns depend on the charge numbers *z*_*P* ^+^_ and *z*_*P* ^–^_. Figure 3a shows that periodic patterns appear only in a restricted parameter region (yellow region P). In particular, this patterned phase (also known as microphase separation^21^) does not exist for symmetric mixtures (diagonal line, Δ*z* = 0). Instead, the system exhibits macrophase separation (blue H + H region) if χ is sufficiently negative, and this region becomes larger for weaker interactions (Figure 3b and Supporting Figure S37). Moreover, the system stays homogeneous for very asymmetric mixtures (teal H region). Collectively, these observations suggest that periodic patterns emerge for sufficiently strong attraction (large −χ) and intermediate charge asymmetry |Δ*z*| between polymers.

**Figure 3:**
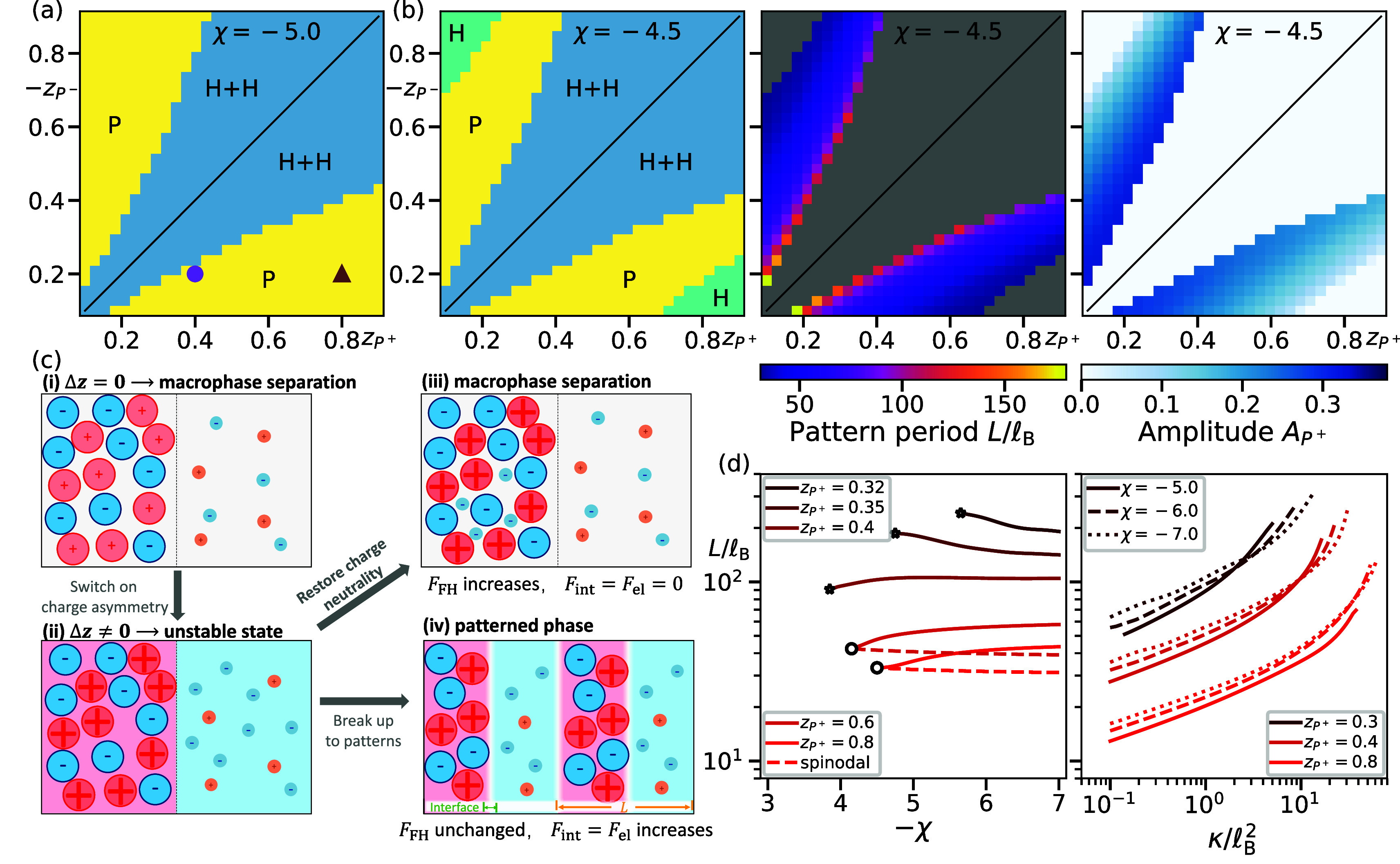
Field theory predicts transitions to patterned phases. (a) Phase diagram as a function of the charge numbers *z*_*P* ^+^_ and *z*_*P* ^–^_ of the polymers revealing parameter regions with the coexistence of two homogeneous phases (H + H) and patterned phases (P) for strong attraction (χ = −5). The colored markers correspond to the two panels in Figure 2. (b) Phase diagram for a weaker attraction (χ = −4.5), including regions with a single homogeneous phase (H). The remaining columns show respective periods *L* and amplitudes *A*_*P* ^+^_ = max­(ϕ_*P* ^+^_) – min­(ϕ_*P* ^+^_) . (c) Schematic explanation of the origin of the patterned phase. The initial phase-separated charge-neutral state in (i) is destabilized due to net charges if charge asymmetry is enabled in (ii). An equilibrium state can be reached either by restoring charge neutrality in (iii) or by breaking the phases into smaller droplets in (iv). (d) Pattern length scale *L* as a function of interaction parameter χ (left, interfacial parameter κ = 10) and κ (right, various χ) for various *z*_*P* ^+^_ at *z*_*P* ^–^_ = −0.2. As |χ| decreases, the patterned phase transitions to macrophase separation (stars) or a homogeneous state (open disk). Dashed lines indicate the most unstable length scale predicted by the linear stability analysis in the Supporting Information. (a–d) Additional parameters are given in Figure 2.

We can understand the emergence of patterned phases starting from the charge-balanced state (Δ*z* = 0, Figure 3c, subpanel (i)). In this case, the polymers form a dense macrophase, while small species (*e* ^+^, *e* ^–^, and *S*) accumulate in the corresponding dilute phase. Here, we have *F* = *F*_FH_ since *F*_el_ = 0 and the energy *F*_int_ of the single interface is negligible in a thermodynamically large system. Switching on charge asymmetry (Δ*z* ≠ 0, subpanel (ii)) destabilizes this state because the macrophases acquire a net charge, so the electrostatic energy *F*_el_ diverges. To minimize the total free energy *F*, the state can change in two fundamentally different ways: it can either restore charge neutrality (subpanel (iii)) or form a patterned phase (subpanel (iv)). In the first alternative, ions move into the dense phase (maintaining macrophase separation, subpanel (iii)), which restores charge neutrality and maintains *F*_el_ = *F*_int_ = 0. However, the ions decrease the concentration of polymers, thus reducing short-range contacts between polymers and increasing *F*_FH_. In the second alternative, the dense phase is broken up to form a pattern of finite length scale *L* (subpanel (iv)). In the simplest case, the polymers maintain a high concentration, implying that *F*_FH_ hardly changes. However, the electrostatic energy *F*_el_ is now finite, and the breakup creates many interfaces; so, both *F*_el_ and *F*_int_ are positive. Taken together, both alternatives increase *F* compared to the neutral initial case, but breakup into the patterned phase is favored for strong attraction (large −χ).

The preceding argument also makes qualitative predictions for pattern period *L* as a proxy for droplet size. Assuming that the density of polymers stays constant in the patterned phase, *F*_FH_ is independent of *L*, so *L* is instead governed by the minimum of *F*_int_ + *F*_el_. Generally, *F*_int_ decreases with increasing *L* (since there are fewer interfaces per unit length), whereas *F*_el_ increases with *L* (since opposite charges are separated further for larger *L*, i.e., exchanging the two central slabs in Figure 3c­(iv) costs electrostatic energy). These arguments predict that *L* increases with a larger κ (increasing *F*_int_), whereas *L* decreases with a larger |Δ*z*| (increasing *F*_el_). We present a more quantitative argument in the Supporting Information, which shows that at the free energy minimum the two energies have opposing dependencies on *L*, d*F*_int_/d*L* = −d*F*_el_/d*L*, leading to a trade-off where *F*_int_ = *F*_el_ at the minimum. We thus generally expect that *L* increases with a larger κ and smaller |Δ*z*|.

We test our predictions by analyzing the patterned phases obtained from field theory. Figure 3b shows that *L* indeed decreases for larger |Δ*z*| (away from the symmetry line), although the patterns completely vanish for large |Δ*z*| where the homogeneous state exhibits the lowest free energy. The right-most panel in Figure 3b shows that the amplitude of ϕ_*P* ^+^_(*x*) decreases with increasing |Δ*z*|, implying that polymers become less concentrated. This is not captured by the simple argument above, suggesting that the system compromises the segregation of polymers (implying a larger *F*_FH_) for a lower electrostatic energy *F*_el_. Indeed we find that *F*_el_ exhibits a nonmonotonous dependency on |Δ*z*| (Supporting Figure S42), and the patterns cease when *F*_el_ vanishes. Concomitantly, the amplitude approaches zero (Figure 3b), suggesting that the transition to the homogeneous phase is continuous.

To also test the influence of interaction strength χ and interfacial parameter κ on pattern size *L*, we performed additional minimizations. Figure 3d confirms a weak influence of χ, whereas *L* increases significantly with κ. Even if we use a density-dependent gradient term in eq 2, which is expected for polymer solutions,^32–35^ the dependence on κ remains the same (Supporting Figure S31). The weak influence of χ is consistent with the behavior in hydrophobic polyelectrolytes, where the cluster size is independent of the strength of the attractive interactions and is governed solely by electrostatics.^23^ However, these length scales cannot be predicted from a simple stability analysis of the homogeneous state (dashed line in Figure 3d; details in the Supporting Information), except at the transition of the patterned phase to the homogeneous state (open circles). This observation, and the fact that the corresponding critical exponent is consistent with the mean-field universality class (Supporting Figure S41), provides additional evidence for a continuous phase transition. Taken together, the numerical solutions of the field theory confirm that *L* (and thus the droplet size) increases with larger κ and smaller |Δ*z*|, while χ has a minor influence beyond driving droplet formation in the first place.

To test whether our predictions hold more generally and in particular in 3D systems, we return to our MD simulations (details described in the Supporting Text). Figure 4a shows that a larger charge asymmetry Δ*z* indeed leads to smaller droplets. We also observe system spanning droplets close to charge neutrality (Δ*z* = 0; yellow symbols), which are consistent with macrophase separation (region H + H in Figure 3). The detailed size distributions shown in Supporting Figure S3 indicate this dependence unambiguously, although finite size effects are visible for small charge asymmetries. Moreover, Figure 4b shows that varying the interaction strength ε_LJ_ between the polymers affects the droplet size only weakly, consistent with the weak influence of χ shown in Figure 3d. In both cases, at weak charge asymmetry, droplet size slightly decreases for larger attraction, conversely to the behavior at strong charge asymmetry. Overall, these data confirm the predictions from field theory, suggesting that droplet size control by net charges is a general phenomenon.

**Figure 4:**
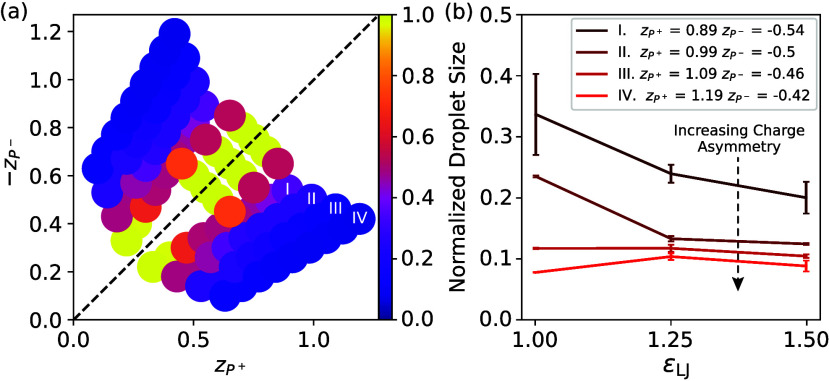
MD simulations corroborate field theory. (a) Simulations at various charge numbers (*z*_*P* ^+^_, *z*_*P* ^–^_) demonstrate that high charge asymmetries result in smaller droplet sizes (the color bar indicates normalized droplet size which is $\frac{\#\;\mathrm{chains}\;\mathrm{in}\;\mathrm{droplet}}{\#\;\mathrm{chains}\;\mathrm{in}\;\mathrm{simulation}}$ ). (b) Droplet size as a function of attraction strength ε_LJ_ for the four systems marked in (a). Decreasing ε_LJ_ mildly increases or decreases the droplet size depending on charge asymmetry.

To assess the relevance of the size-control mechanism to biomolecular condensates, we now constrain the mean-field theory parameters to values that are more representative of cellular conditions. Because the typical ion size is on the order of the Bjerrum length,^36^ we set $v=l_{B}^{3}$ and choose ν_*e* ^+^_ = ν_*e* ^–^_ = 1 with *z*_*e* ^+^_ = −*z*_*e* ^–^_ = 1. A typical example for a negatively charged biopolymer is RNA, with an effective charge density of ∼1 nm^–3^,^37^ so we set the charge number per unit volume *v* to *z*_*P* ^–^_ = −0.5. In contrast, we consider proteins as the positively charged polymer; we chose *z*_*P* ^+^_ = 0.2 since they typically have lower charge densities than RNA.^38^ We keep the volume fraction of the polymers at 10% as a representative estimate and to stay within the droplet forming regime, but we vary the ion concentration ϕ̅_ion_ = ϕ̅_*e* ^+^_ + ϕ̅_*e* ^–^_ and the polymer volume ν_*P*_ to explore these crucial parameters. Figure 5a shows that the mean-field theory still predicts a patterned phase, even when the polymer volume ν_*P*_ is much larger than the ion volume. We find that the period *L* is insensitive to variations in ν_*P*_ for ν_*P*_ ≫ 1, presumably because the entropic contributions of the large polymers play a minor role. Instead, *L* strongly depends on the salt concentration, which is controlled by ϕ̅_ion_ (Figure 5b). Since a salt concentration of around 100 mM corresponds to a volume fraction of ϕ̅ ∼ 0.02, our results imply that patterned phases can form for much higher salt concentrations. Taken together, these results suggest that condensate size control is, in principle, possible at physiological salt concentrations.

**Figure 5:**
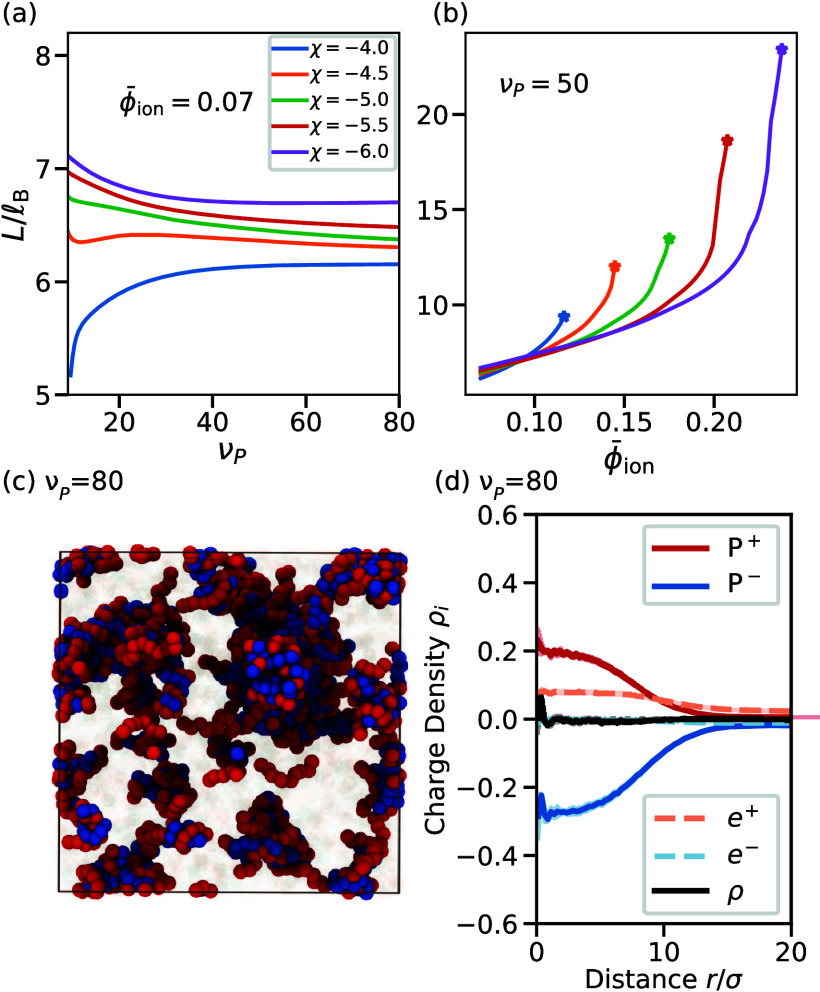
Size control of realistic condensates is restricted to molecular sizes. (a) Stable pattern length scale *L* predicted by the field theory as a function of the molecular volume $\nu_{P}l_{B}^{3}$ of both polymer types for various interaction strengths χ. (b) *L* as a function of the volume fraction ϕ̅_ion_ = ϕ̅_*e* ^+^_ + ϕ̅_*e* ^–^_ of ions for χ given in panel a. Stars indicate the transition to macrophase separation. (a, b) Additional parameters are $v=l_{B}^{3}$ , ν_*P* ^+^_ = ν_*P* ^–^_ = ν_*P*_, ν_*e* ^+^_ = ν_*e* ^–^_ = 1, ϕ̅_*P* ^–^_ = ϕ̅_*P* ^+^_ = 0.1, *z*_*P* ^+^_ = 0.2, *z*_*P* ^–^_ = −0.5, and $\kappa =0.1l_{B}^{2}$ . (c) An MD simulation snapshot of the ν_*P*_ = 80 case. (d) The charge density profile of the largest droplet in ν_*P*_ = 80 simulations indicates a vanishing net charge, which suggests a thermodynamically large droplet in the absence of finite-sized effects coexisting with a dilute phase containing clusters of molecular sizes. (c,d) MD simulation parameters are provided in section I.F of the Supporting Text.

In all physiologically relevant cases, the resulting pattern length scale *L* is only about an order of magnitude larger than the Bjerrum length $l_{B}$ , implying that the predicted condensates contain at most a few polymer chains. Since number fluctuations are then significant (particularly at large ν_*P*_), mean-field theory is not very reliable. Consequently, we also ran additional MD simulations, varying ν_*P*_ from 20 to 80 in intervals of 10. Histograms of the droplet sizes from these simulations are presented in Supporting Figures S21–23, and an example snapshot of the ν_*P*_ = 80 simulation is visualized in Figure 5c. This snapshot clearly shows small clusters (similar to ref 13). However, in contrast to the mean-field theory, the MD simulations predict a vanishing net charge for the largest cluster (Figure 5d for ν_*P*_ = 80, Supporting Figure S25 for other ν_*P*_), which suggests a thermodynamically large condensate under physiological conditions (i.e., for a simulation not subject to finite-sized effects). Despite this difference, both approaches predict small clusters under physiological conditions. In particular, the MD simulations indicate the existence of small clusters in the dilute phase coexisting with a larger droplet that is qualitatively different from a charge symmetric case (Supporting Figure S23). Taken together, both the field theoretic calculations and the MD simulations indicate charge asymmetry produces a distribution of droplet sizes restricted to molecular length scales, but a patterned phase with large period is likely only relevant if ions can be effectively expelled from droplets.

We described a mechanism of droplet size control that relies on electrostatic effects that are relevant when charge screening is weak. This is the case when ions are expelled from droplets and thus cannot neutralize the polymers, resulting in a net charge in our MD simulations (Supporting Figure S1) and the field theory (Figure 2). Such systems cannot be described as a dilute electrolyte with mobile ions, e.g., using Debye–Hückel theory,^9^ so the droplet size is not simply limited by the Debye screening length. Even for small ions that can neutralize condensed polymers, our MD simulations suggest clusters on the order of molecular length scales can coexist with a thermodynamically large droplet, implying that ions should generally be treated explicitly for condensates involving complex, charged molecules^39,40^ and similar systems.^41–43^

The described droplet size control essentially emerges from a trade-off between short-range attraction driving phase separation and long-range electrostatic repulsion if droplets accumulate net charges. At weak charge asymmetry, the system exhibits macrophase separation since the attraction dominates. Conversely, for strong charge asymmetry, the system will stay homogeneous to avoid accumulation of net charges. Between these two extremes, the patterned phases emerge as a compromise between phase separation and the accumulation of net charge. While similar descriptions of patterned phases (e.g., electrostatic microphase separation) exist in the polyelectrolyte literature,^21,23,30^ we have demonstrated that modulating molecular charge density—a task performed constantly within cells via chemical modifications to biomolecules which is known to regulate biomolecular condensates^44^—can produce dramatic changes in droplet sizes. Such patterned states also emerge in simpler phase separating systems with Coulomb-like interactions.^45–49^ In particular, when elastic effects mediate long-range repulsion, the patterned phase also exhibits a continuous transition to the homogeneous state.^28^ This similarity suggests that droplet size can be generally controlled when the short-range attraction leading to phase separation is opposed by some long-range repulsion,^50^ reminiscent of classical pattern formation.^51^ Another example for this mechanism are chemically active droplets, where long-range repulsion emerges from a reaction–diffusion system,^27,52–54^ which can in fact be interpreted using an electrostatic analogy.^55–57^

Here, we focused on the most basic mechanism of droplet size control by net charges. For instance, we assumed that polymers have a fixed charge, whereas realistic molecules will exhibit charge regulation; therefore, their behavior will depend on pH as well as dynamic modifications (e.g., post-translational modifications of amino acids). Molecules involved in typical biomolecular condensates are also much more complex: their charge distribution will be inhomogeneous, the nonspecific interactions will be heterogeneous and might depend on charge, and the polymer structure cannot be ignored, particularly for nucleic acids and proteins.^58,59^ Such effects inform short-range interactions within biomolecular condensates,^1,3^ but the impact on long-range electrostatics is less understood. Consequently, the behavior of real biomolecules might thus interpolate between simple polyelectrolytes^30^ and our model, where short-range attraction is not affected by salt. In any case, both approaches suggest that charge asymmetry could stabilize small condensates, which cannot be explained by the simple Debye–Hückel theory. Therefore, it will be interesting to see how the described size control and ion distribution behave in more complex systems as well as how these charge asymmetries may affect condensates inside cells.

## Supplementary Material



## Notes

Supporting text and figures for the MD simulations and the field theory. Details pertaining to molecular dynamics simulations including: Simulation procedure; Additional simulation results pertaining to main text figures; Demonstration of simulation equilibrium statistics; Simulations with reduced ion sizes; Simulations run in the overdamped limit; and Simulations performed at physiological conditions. Details pertaining to field theory calculations including: Free energy minimzation; Numerical minimization method; Linear stability analysis; Quantification of ion concentrations; Exploration of a concentration-dependent gradient energy; Chain number in droplets; and Additional results pertaining to main text figures (PDF)
