# Supplementary material for: Theory of Condensate Size Control by Molecular Charge Asymmetry
Source: ACS Macro Lett. 2025 Sep 29;14(10):1484–91. doi: 10.1021/acsmacrolett.5c00342 (PMC12548355; doi:10.1021/acsmacrolett.5c00342)
Supplement: Supplementary file 1 [file mz5c00342_si_001.pdf]

Supplementary Material for "Theory of Condensate Size Control by Molecular Charge Asymmetry"

Chengjie Luo,[1,*] Nathaniel Hess,[2,*] Dilimulati Aierken,[2,3]
Yicheng Qiang,[1] Jerelle A. Joseph,[2,3,†] and David Zwicker[1,†]

[1]*Max Planck Institute for Dynamics and Self-Organization, Am Faßberg 17, 37077 Göttingen, Germany*
[2]*Department of Chemical and Biological Engineering, Princeton University, Princeton, NJ 08544, USA*
[3]*Omenn–Darling Bioengineering Institute, Princeton University, Princeton, NJ 08544, USA*
(Dated: August 1, 2025)

CONTENTS

I. Details of the molecular dynamics (MD) simulations 1
A. Simulation procedure 1
B. Additional result figures 2
C. Demonstration of simulation equilibration 7
D. Simulations with reduced ion size 13
E. Simulations in the overdamped limit 20
F. Simulations at physiological conditions 24

II. Details of the field theory 30
A. Free energy minimization 30
B. Numerical minimization method 32
C. Linear stability analysis 34
D. Quantification of ion concentration 34
E. No Qualitative Change from Concentration-Dependent Gradient Energy 36
F. Number of chains increases with κ and ion concentration, and decreases with charge asymmetry 36
G. Additional result figures 37

References 46

I. DETAILS OF THE MOLECULAR DYNAMICS (MD) SIMULATIONS

Here we present additional details on the MD simulations performed in this investigation. In particular, we provide additional details on the simulation procedure in subsection I A. Additional figures pertaining to the data presented in the main text are provided in subsection I B. In subsection I C we show data that demonstrates the simulations discussed in the main text are sampling equilibrium statistics. Further, we explore the impact of ion size on the mechanism of droplet size control by investigating a subset of simulations with reduced ion sizes in subsection I D. We also validate that the damping constant of the Langevin thermostat does not alter simulation results if simulated in the overdamped limit in subsection I E. Finally, in subsection I F we provide additional details on our exploration of whether the mechanism of condensate size control is present under physiological conditions (i.e., a situation in which ions smaller than both the Bjerrum length and the monomers on the charged polymers).

A. Simulation procedure

All simulations are performed in the MD software LAMMPS [1]. MD simulations are run in the NVT ensemble with neutral mixtures of charged polymers and ions in implicit solvent (Main Figure 1a). The equations of motion are integrated using the velocity-Verlet algorithm and the temperature is controlled using a Langevin thermostat (discussed in more detail below). All particles (i.e., monomers and ions) are modeled with a unit size σ and a unit mass m. Note that all simulation quantities reported in this SI and in the main text are in reduced Lennard–Jones units.

* These two authors contributed equally
† To whom correspondence may be addressed. Email: jerellejoseph@princeton.edu and david.zwicker@ds.mpg.de

The total energy of the simulation system is given by:

$$U = U_{\rm el} + U_{\rm P} + U_{\rm EV}, \tag{S1}$$

where $U_{\rm el}$ describes the long-range electrostatic interactions between all charged species (i.e., charged monomers on the polymers as well as ions), $U_{\rm P}$ describes the same chain bonded interactions and short-range heterotypic attraction between positively charged monomers P^+ and negatively charged monomers P^-, and $U_{\rm EV}$ describes the ion–ion, ion–polymer, and homotypic polymer–polymer excluded-volume interactions. More specifically, $U_{\rm el}$ is modeled using Coulomb's potential:

$$U_{\rm el} = \sum_{i,j} \frac{z_i z_j}{\varepsilon r_{ij}}. \tag{S2}$$

In this form, z_i and z_j describe the charge on species i and j, r_{ij} is the distance between the charged species, and the dielectric constant is ε. Next, $U_{\rm P}$ models the combination of the bonded potential $V_{\rm bond}$ and the Lennard-Jones potential $V_{\rm LJ}$ with cut-off and shifting:

$$U_{\rm P} = \sum_{i=1}^{N} \sum_{j=1}^{n-1} V_{\rm bond}(r_{j+1,j}) + \sum_{i,j} \left(V_{\rm LJ}(r_{i,j}) - V_{\rm LJ}(r_{\rm c}) \right). \tag{S3}$$

Here, N is total number of polymers in the system, n is the polymer length. The bonded potential is modeled using a harmonic potential $V_{\rm bond}(r) = K(r - r_{\rm b})^2$. As for the Lennard-Jones potential we use:

$$V_{\rm LJ}(r) = 4\epsilon_{\rm LJ} \left[\left(\frac{\sigma}{r}\right)^{12} - \left(\frac{\sigma}{r}\right)^{6} \right],\ r \leq r_{\rm c}. \tag{S4}$$

Finally, for the excluded volume for all the particles (besides interactions between P^+ and P^-), we use the Weeks–Chandler–Andersen (WCA) potential

$$U_{\rm EV} = \sum_{i,j} \left[V_{\rm LJ}(r_{i,j}) + \epsilon_{\rm LJ} \right],\ r_{i,j} \leq 2^{1/6}\sigma. \tag{S5}$$

In all simulations, we use a box size of $60 \times 60 \times 60\ \sigma^3$ with our species volume fractions of $\phi_{P^+} = \phi_{P^-} = 0.1$ and $\phi_{e^-} = z_{P^+}\phi_{P^+}$ and $\phi_{e^+} = -z_{P^-}\phi_{P^-}$. This corresponds to $N = 512 + 512 = 1024$ polymers where each polymer is composed of $n = 10$ bonded monomers. We use a bond coefficient of $100.0\epsilon_{\rm LJ}$ and an equilibrium distance $r_{\rm b} = 1.0\sigma$. For the Lennard-Jones interactions, we use a cut-off of $r_{\rm c} = 2.5\sigma$ and attraction strength of $\epsilon_{\rm LJ} = 1.0$. A strength of $\epsilon = 1.0$ is also used for excluded volume interactions of the WCA potential. The charge of polymers is varied by simulation. The charge of ions is set to have a magnitude of 1.0. Electrostatic interactions are calculated using a PPPM grid [2] for interactions beyond 2.5σ with relative error in forces of less than 10^{-4}. Electrostatic interactions within 2.5σ are calculated directly. The dielectric constant is set to $\varepsilon = 80.0$.

Each simulation trajectory is first annealed at a temperature of $k_{\rm B}T/\epsilon_{\rm LJ} = 5.0$ for $10^4\tau$ using a Langevin thermostat with a damping of 100.0τ. Thereafter, the temperature of each simulation is set to $k_{\rm B}T/\epsilon_{\rm LJ} = 1.0$ also using a Langevin thermostat with a damping of 100.0τ. Simulation trajectories are then run for $10^6\tau$. The last $5 \times 10^5\tau$ is collected for data analysis, sampling a frame every 500τ. We use a timestep of 0.005τ in the simulations. Additionally, all simulations in this study are performed in triplicate. Errors depicted in simulation figures correspond to the standard error between these simulation replicates during the sampling period of $5 \times 10^5\tau$.

In the analysis of MD simulations, clusters (droplets) are defined using a cutoff of 2.0σ between polymers. The trajectory analysis software Ovito [3] is used to generate cluster data from simulation frames. An in-house code is used to generate radial density profiles from an Ovito [3] cluster analysis pipeline.

B. Additional result figures

Additional figures are presented in this section corresponding to results from MD simulations discussed in the main text. Figure S1 presents radial charge density profiles from MD simulations at ($z_{P^+} = 0.55$, $z_{P^-} = -0.55$), ($z_{P^+} = 0.64$, $z_{P^-} = -0.3$), and ($z_{P^+} = 1.05$, $z_{P^-} = -0.34$). See Main Figure 1b for a visualization of these droplets. Figure S2 presents the density profiles from the MD simulations for the systems listed above. Figure S3 shows normalized droplet size distributions corresponding to four charge pairings in Main Figure 4a. As in all figures in the text, normalized droplet size refers to the average number of chains in a droplet divided by the total number of chains in the simulation (normalized droplet size $= \frac{\#\ \text{chains in droplet}}{\#\ \text{chains in simulation}}$). Figure S4 analyzes the relationship between charge asymmetry and droplet size across three different values of $\epsilon_{\rm LJ}$. Figure S5 depicts the standard error in average droplet sizes across simulation replicates. Finally, Figure S6 quantifies the standard deviation in normalized droplet sizes within the simulations presented in Main Figure 4a.

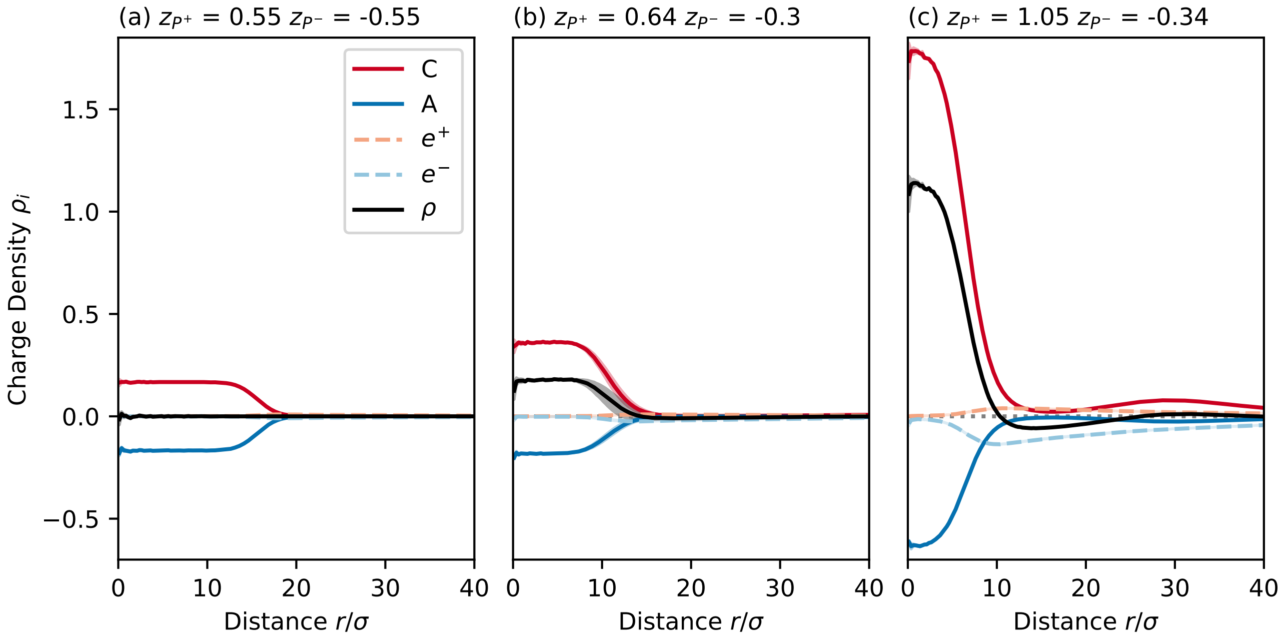

FIG. S1. **Radial charge density profiles indicate charge asymmetry controls droplet size.** Radial charge density profiles for (a) $z_{P+} = 0.55$, $z_{P-} = -0.55$ (b) $z_{P+} = 0.64$, $z_{P-} = -0.3$ and (c) $z_{P+} = 1.05$, $z_{P-} = -0.34$. Shaded regions indicate the standard error from three independent simulation replicates. The profiles indicate that increasing charge asymmetry decreases droplet size. They also demonstrate that ions are excluded from the dense phase. See Main Figure 1b for visualization of these droplets.

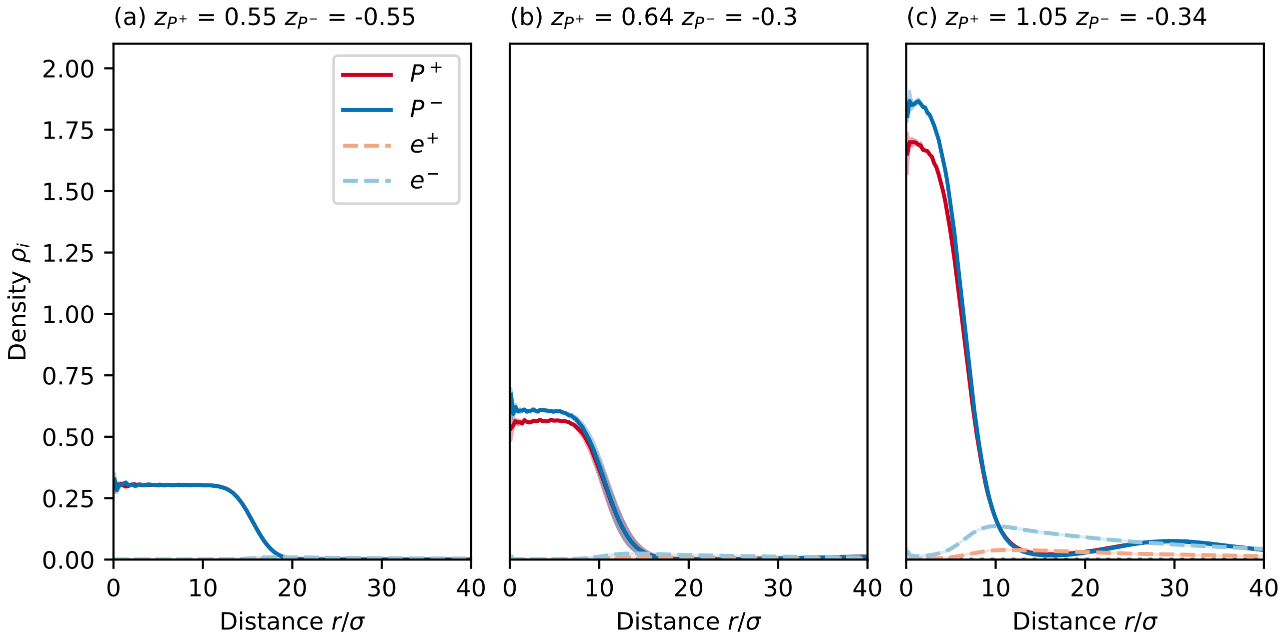

FIG. S2. **Radial density profiles indicates disparate concentrations of charged polymers in dense phase.** Radial density profiles for (a) $z_{P+} = 0.55$, $z_{P-} = -0.55$ (b) $z_{P+} = 0.64$, $z_{P-} = -0.3$ and (c) $z_{P+} = 1.05$, $z_{P-} = -0.34$. Shaded regions indicate the standard error from three independent simulation replicates.

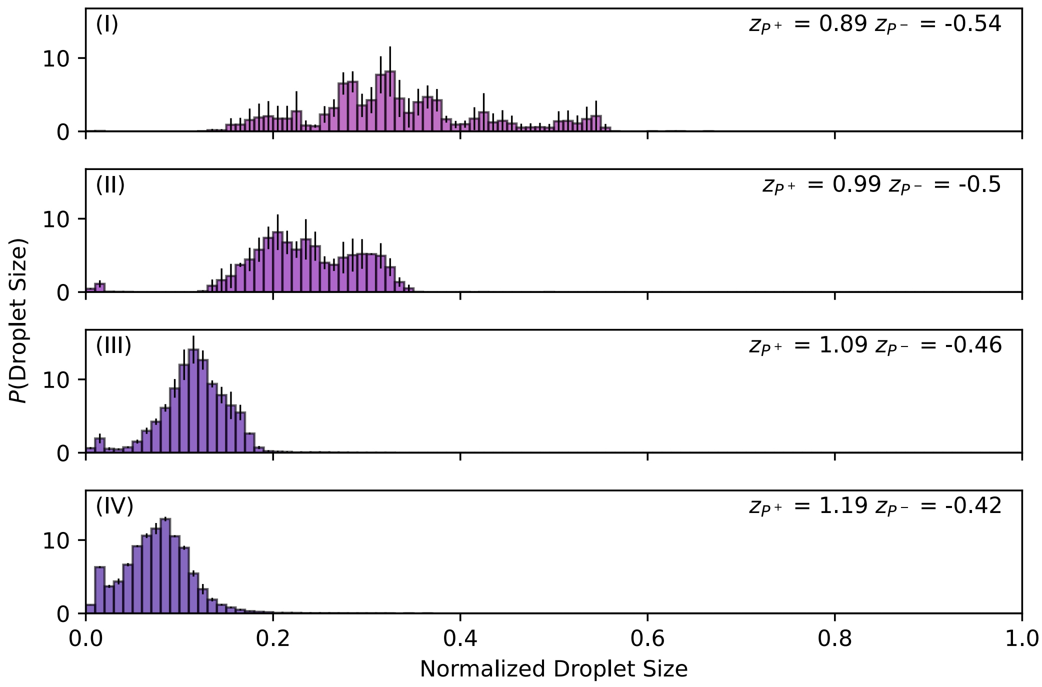

FIG. S3. **Charge asymmetries create distributions of droplet sizes.** Four simulation systems, (I) $z_{P+} = 0.89$, $z_{P-} = -0.54$ (II) $z_{P+} = 0.99$, $z_{P-} = -0.5$ (III) $z_{P+} = 1.09$, $z_{P-} = -0.46$ (IV) $z_{P+} = 1.19$, $z_{P-} = -0.42$, corresponding to Main Figure 4a, are plotted in histograms of normalized droplet sizes. Error bars indicate the standard error across histograms generated from three independent simulation replicates. At larger charge asymmetries (III and IV), a smaller average droplet size is favored. Finite-sized effects prevail at smaller charge asymmetries (I and II) indicated by a wide distribution of droplet sizes and high errors. Note that droplets of less than 10 polymer chains are excluded from the histogram.

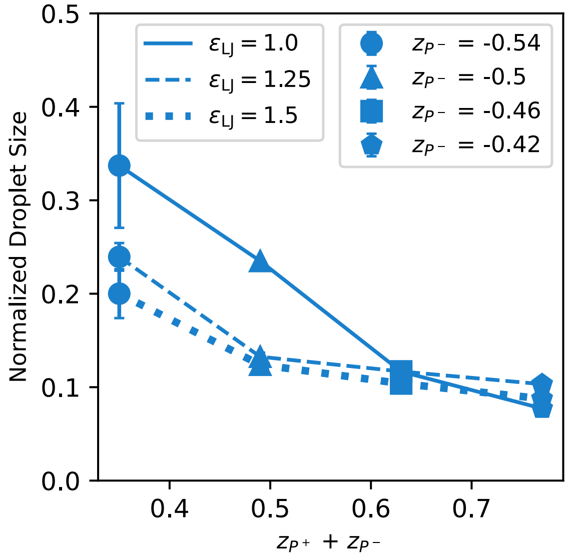

FIG. S4. **Charge asymmetry's effect on droplet size is preserved at higher polymer attraction strengths.** The normalized droplet size for four charge pairings $[(z_{P+} = 0.89, z_{P-} = -0.54), (z_{P+} = 0.99, z_{P-} = -0.5), (z_{P+} = 1.09, z_{P-} = -0.46), (z_{P+} = 1.19, z_{P-} = -0.42)$ corresponding to I-IV in Main Figure 4a] at three different attraction strengths between oppositely charged polymers ($\epsilon_{\rm LJ} = 1.0, 1.25, 1.5$). As charge asymmetry is increased, the normalized droplet size in the simulations decreases regardless of the value of $\epsilon_{\rm LJ}$. Error bars represent the standard error in average normalized droplet size for three independent simulation replicates.

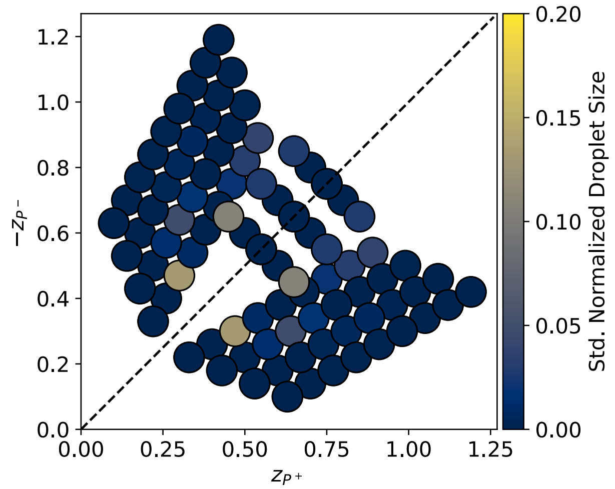

FIG. S5. **Error on average simulation droplet sizes indicate finite-sized effects.** The standard error in the average droplet size (across three simulation replicates) for the charge pairings plotted in Main Figure 4a is shown. At intermediate charge asymmetries (charge asymmetries closer to the diagonal that still present a patterned phase), the presence of finite-sized effects is demonstrated by sustained droplets of different sizes (yellow/gray circles).

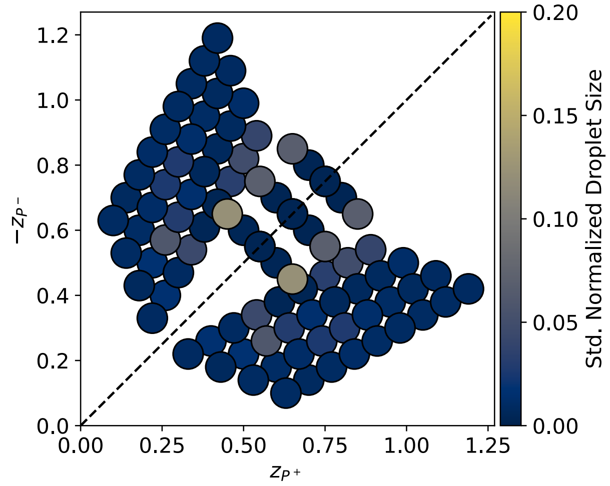

FIG. S6. **Average standard deviation of droplet sizes within simulation replicates indicate finite-sized effects.** The standard deviation in the normalized droplet sizes plotted in Main Figure 4a averaged across three replicate simulations. Larger standard deviation (yellow/gray circles) for moderate charge asymmetries are likely due to finite-sized effects resulting in size discrepancies among sustained droplets (see Fig. S3).

C. Demonstration of simulation equilibration

We consider a subset of the systems discussed in the main text to demonstrate our simulations have reached equilibrium. These simulations include ($z_{P+} = 0.89$, $z_{P-} = -0.54$), ($z_{P+} = 0.99$, $z_{P-} = -0.5$), ($z_{P+} = 1.09$, $z_{P-} = -0.46$), and ($z_{P+} = 1.19$, $z_{P-} = -0.42$) each of which was simulated with three independent replicates. To demonstrate equilibrium, we follow a three step process:

1. Begin with a simulation containing only 512 positively charged polymers and 512 negatively charged polymers at the same volume fraction discussed in subsection I A above. Simulate this mixture in the NpT ensemble with a Langevin thermostat ($T = 1.0$ and damping of 100.0τ) and a Berendsen barostat ($p = 5.0$ and damping of 100.0τ). This simulation is run for 2500τ. Turning off the Berendsen barostat, the simulation is progressed in the NVT ensemble under the same Langevin thermostat for an additional 2500τ. In this simulation, we use Debye–Hückel electrostatic interactions with a screening length of 5σ to ensure one large droplet is formed (i.e., 5σ is shorter than the predicted equilibrium droplet radii for all tested systems in the main text). This step produces one large droplet in a confined simulation box.

2. Increase the box size to restore the proper volume fraction of polymers. Add the requisite number of ions to the simulation box to achieve the desired volume fractions (i.e., charge neutrality in excess salt). We then simulate in the NVT ensemble under the same Langevin thermostat as above for 5000τ. For this step, we use Debye–Hückel electostatic interactions with a screening length of 2σ to ensure that electrostatic repulsions are minimized and the single large droplet remains stable.

3. Remove Debye–Hückel screening. Run a $10^6\tau$ simulation, collecting the last $5 \times 10^5\tau$ for analysis. This simulation is performed exactly as in subsection I A but without the thermal annealing step.

This process ensures that each simulation begins with one large droplet that contains all polymer chains, which is facilitated by the compression from the barostat and Debye–Hückel screened electrostatic interactions with a short screening length. From this point, the simulations progress exactly as they are performed in subsection I A but from this different initial state (one large droplet instead of thermal annealing). Below we compare the results of these simulations to those presented in the main text and subsections I A-I B above. In particular, we compare the histograms of droplet sizes (Figure S7) and the radial charge density profiles (Figures S8, S9). The similarity in the results for simulations that follow the procedure outlined in this section versus those that follow the procedure outlined in subsection I A provide strong evidence that we are sampling well-equilibrated systems.

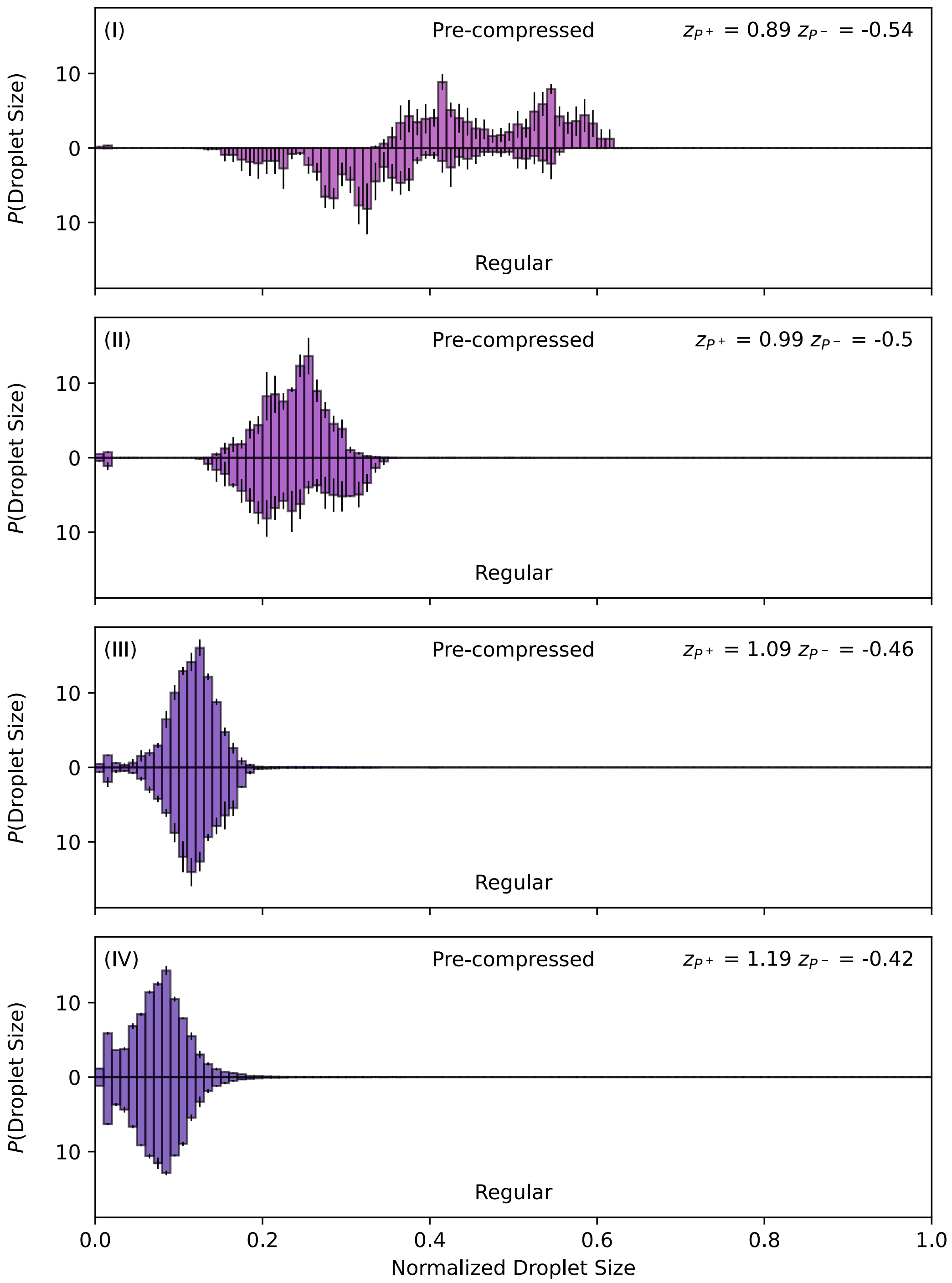

FIG. S7. **Normalized droplet size distributions for pre-compressed versus regular simulations.** The droplet size distributions for simulations that follow the 'pre-compressed' (top) protocol (explained above) versus the 'regular' (bottom) protocol (explained in subsection I A) are presented. The distributions are very similar for both simulation protocols with the largest discrepancy appearing in the simulations for the charge pairing of ($z_{P+} = 0.89$, $z_{P-} = -0.54$). The difference in the distributions for this simulation condition may be due to finite-sized effects creating long lasting droplets of various sizes that are difficult to quantitatively reproduce in two sets of simulation triplicates. Note that droplets of less than 10 polymer chains are excluded from the histogram.

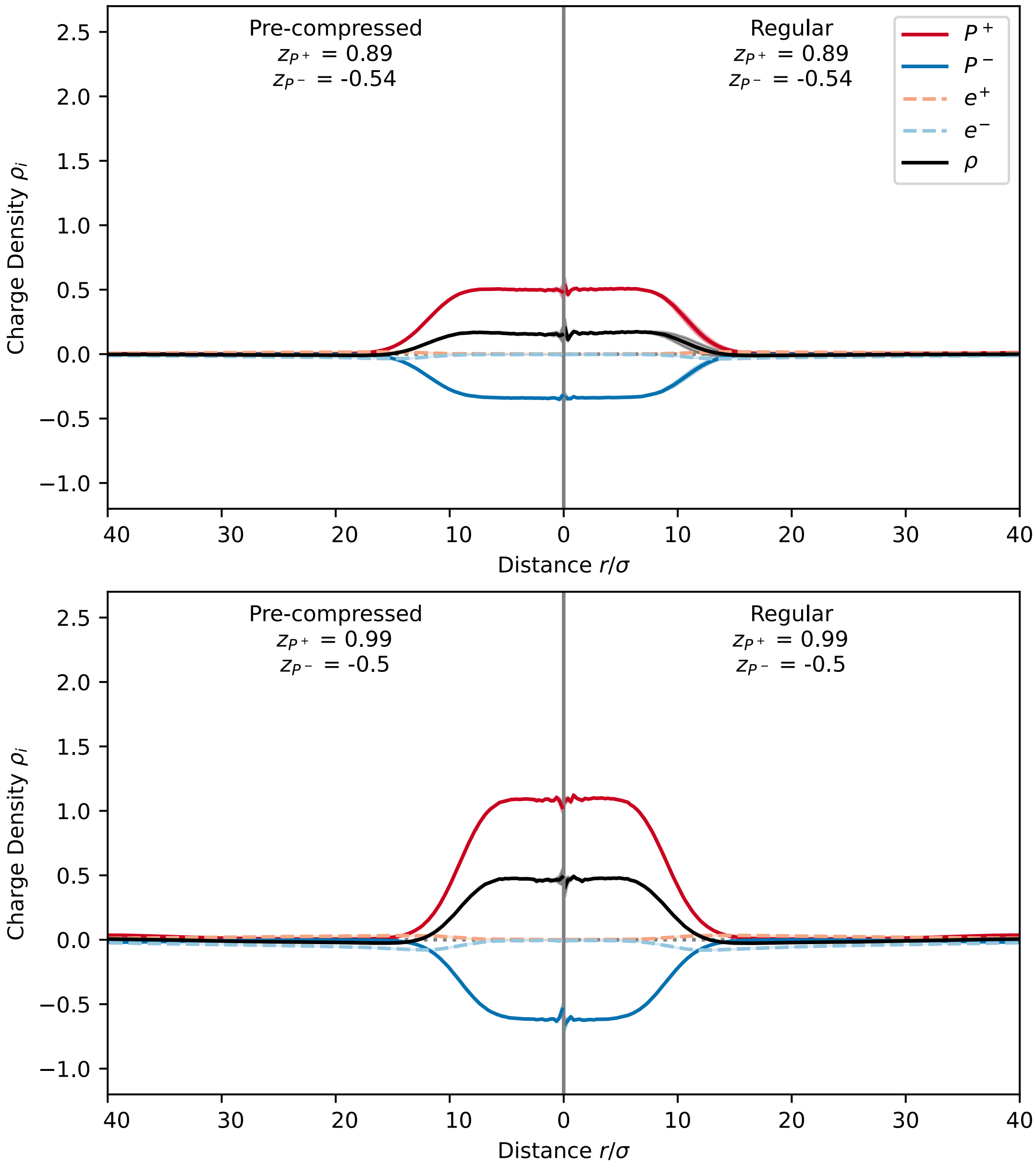

FIG. S8. **Radial charge density profiles for pre-compressed versus regular simulations.** The radial density profiles are presented for the 'pre-compressed' (left) versus 'regular' (right) simulation protocols for the charge pairings of ($z_{P+} = 1.09$, $z_{P-} = -0.46$) and ($z_{P+} = 1.19$, $z_{P-} = -0.42$). The similarity between these distributions demonstrates that the regular simulations are sampling an equilibrated state.

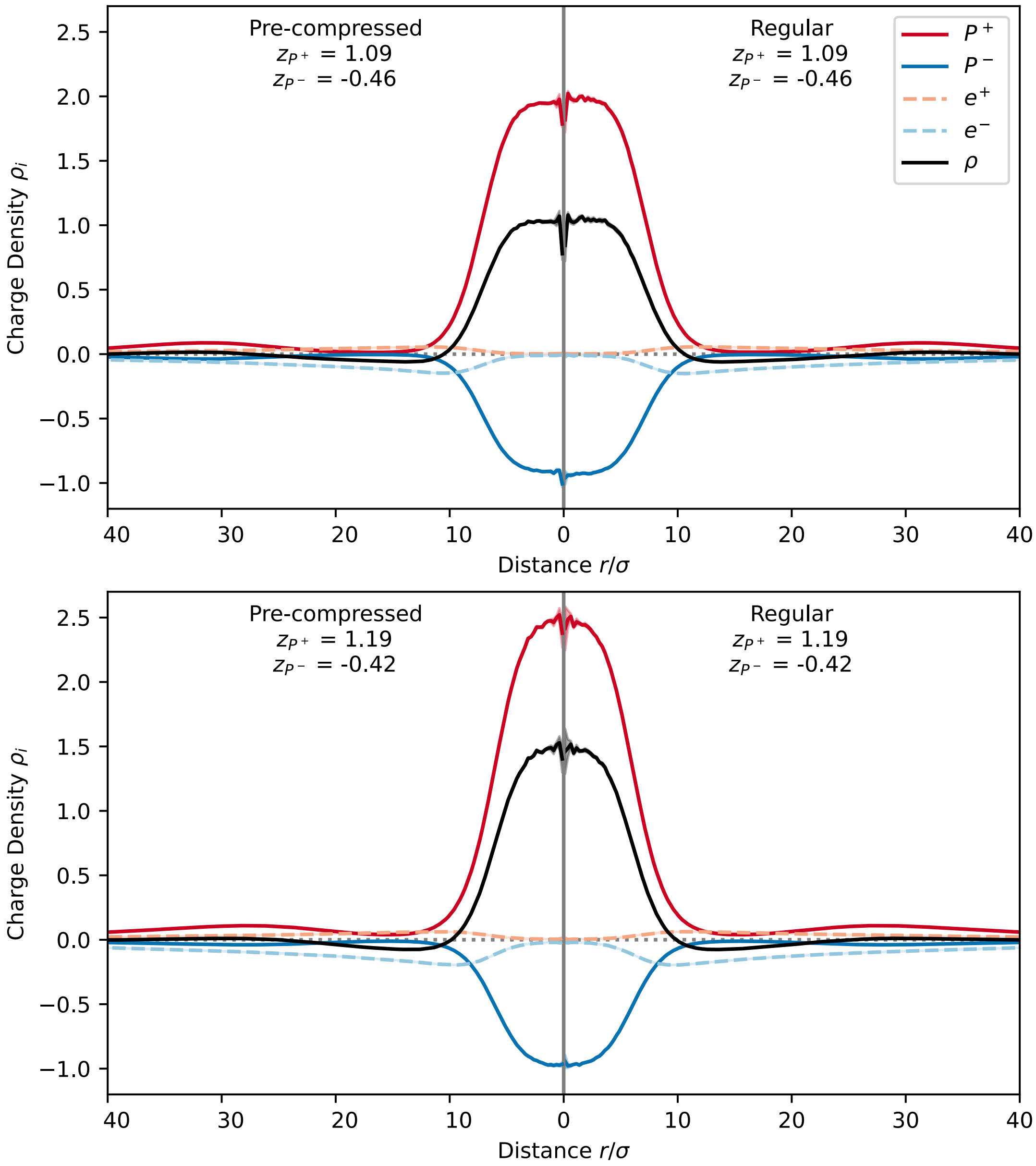

FIG. S9. **Radial charge density profiles for pre-compressed versus regular simulations.** The radial density profiles are presented for the 'pre-compressed' (left) versus 'regular' (right) simulation protocols for the charge pairings of ($z_{P+} = 0.89$, $z_{P-} = -0.54$) and ($z_{P+} = 0.99$, $z_{P-} = -0.5$). The similarity between these distributions demonstrates that the regular simulations are sampling an equilibrated state.

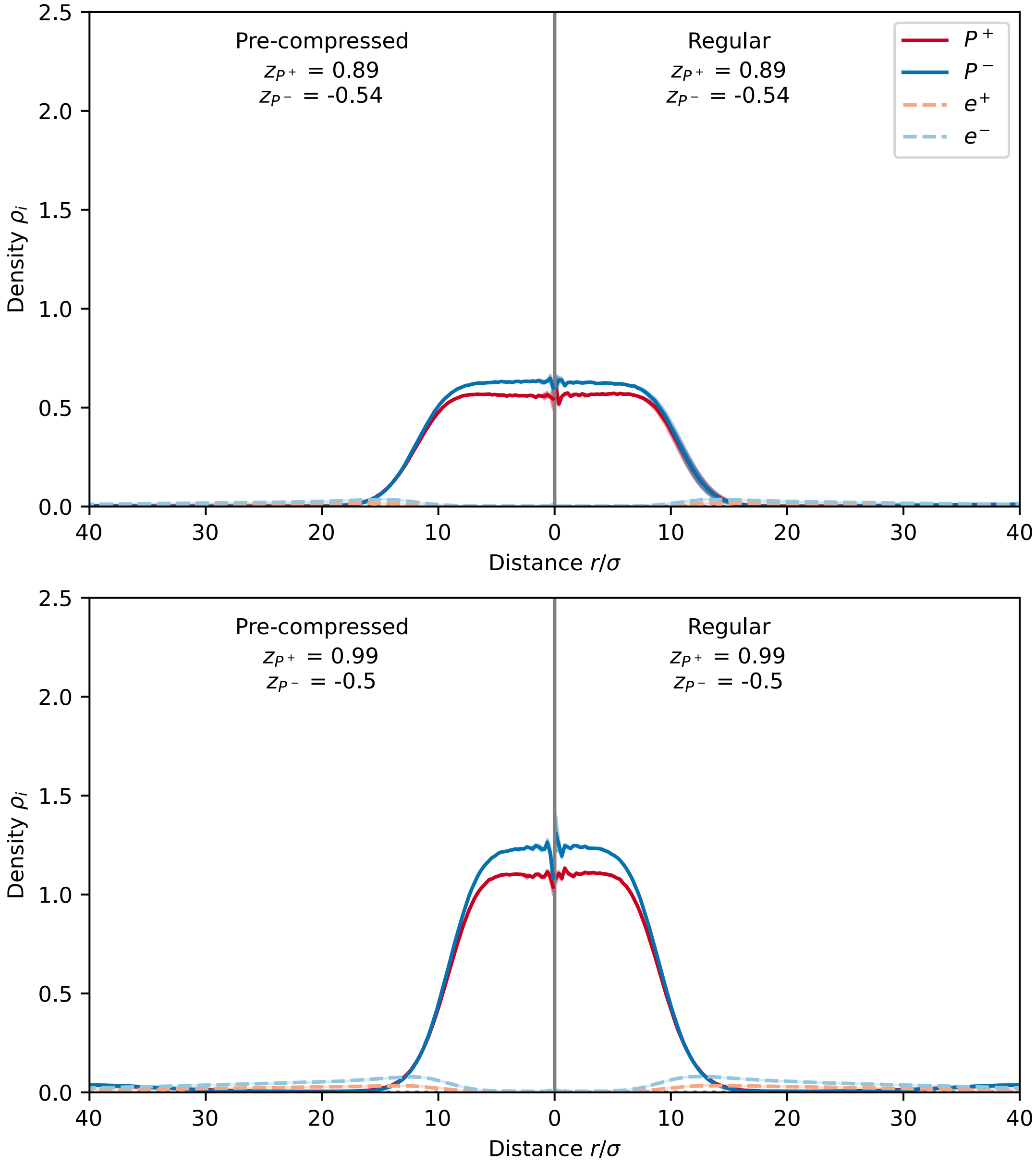

FIG. S10. **Radial density profiles for pre-compressed versus regular simulations.** The radial density profiles are presented for the 'pre-compressed' (left) versus 'regular' (right) simulation protocols for the charge pairings of ($z_{P+} = 1.09$, $z_{P-} = -0.46$) and ($z_{P+} = 1.19$, $z_{P-} = -0.42$).

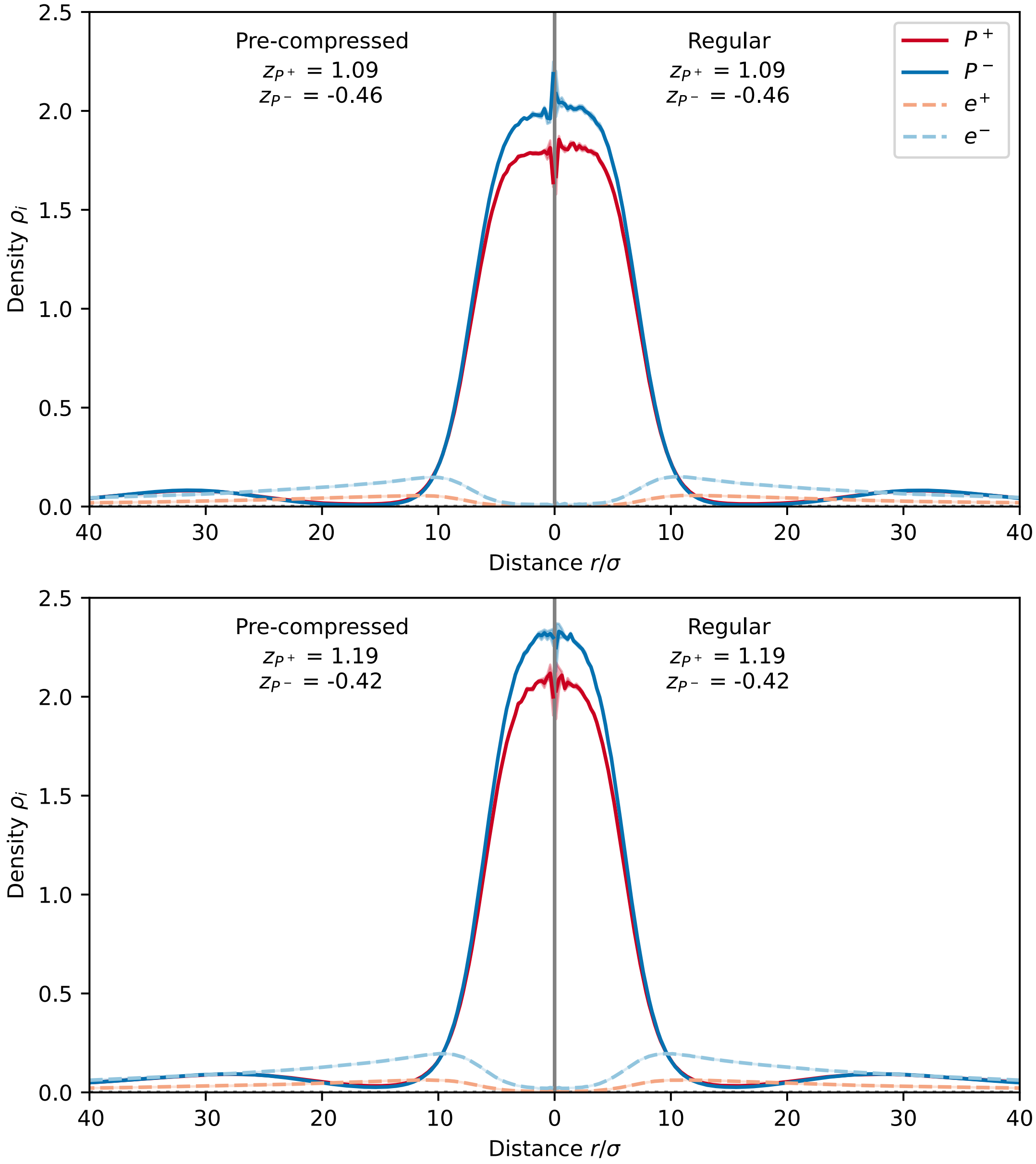

FIG. S11. **Radial density profiles for pre-compressed versus regular simulations.** The radial density profiles are presented for the 'pre-compressed' (left) versus 'regular' (right) simulation protocols for the charge pairings of ($z_{P+} = 0.89$, $z_{P-} = -0.54$) and ($z_{P+} = 0.99$, $z_{P-} = -0.5$).

D. Simulations with reduced ion size

In this section we provide results that suggest the mechanism of size control via charge asymmetry is robust to ion size—at least with the parameters used in this investigation. To do this, we took a subset of simulations—($z_{P+} = 0.89$, $z_{P-} = -0.54$), ($z_{P+} = 0.99$, $z_{P-} = -0.5$), ($z_{P+} = 1.09$, $z_{P-} = -0.46$), ($z_{P+} = 1.19$, $z_{P-} = -0.42$)—and repeated our simulation procedure discussed in subsection I A with a reduced ion size of $\sigma = 0.1$. This reduces the volume of a single ion to be $1/1000\text{th}$ the volume of a polymer monomer. The mass of the ions is still set to $m = 1$ for stability of the time integration (i.e., to preserve the same timestep as used previously). Additionally, in these simulations the interactions between polymers and ions are still modeled with the WCA potential, but the σ values of cross-interactions are found using a geometric mixing rule. These repulsive interactions are also still modeled with an $\epsilon = 1.0$. Interactions between polymers are the same as discussed in subsection I A. Finally, electrostatics are also treated identically to what was discussed in subsection I A.

The simulations of reduced ion size were repeated in triplicate. The results presented below compare these simulations with reduced ion size to those performed under the regular simulation protocol outlined in subsection I A. These results include a comparison of the normalized droplet size histograms (Figure S12) and comparisons of radial charge density profiles (Figures S13, S14). The similarity between the droplet size histograms and the charge density profiles indicate that the mechanism of droplet size regulation may be robust to ion size. However, the most notable difference caused by the reduced ion size is the increased penetration of ions into the dense phase (Figures S13, S14). This may indicate that as the discrepancy in polymer and ion sizes becomes quite large, ions will be able to invade the dense phase and neutralize the droplet thereby enabling full coarsening. Thus, the exact details of any given system of interest (magnitude of charge asymmetry, ion size, polymer length and interaction strength, etc.) may be very important in determining whether electrostatic interactions can contribute to droplet size control.

We also performed additional simulations to determine whether the simulations at a reduced ion size had reached equilibrium. To accomplish this, we ran the charge pairings simulated above—($z_{P+} = 0.89$, $z_{P-} = -0.54$), ($z_{P+} = 0.99$, $z_{P-} = -0.5$), ($z_{P+} = 1.09$, $z_{P-} = -0.46$), ($z_{P+} = 1.19$, $z_{P-} = -0.42$)—according to the procedure outlined in subsection I C (besides for replacing regular sized ions with ions of a reduced size). As was done in subsection I C, we ran each system in triplicate and compared droplet size histograms (Figure S15) and radial charge density profiles (Figures S16, S17) to the simulations performed with protocol from subsection I A (but with reduced ion size). These comparisons demonstrate that we are likely sampling an equilibrated system for the ($z_{P+} = 0.99$, $z_{P-} = -0.5$), ($z_{P+} = 1.09$, $z_{P-} = -0.46$), and ($z_{P+} = 1.19$, $z_{P-} = -0.42$) charge pairings. For the ($z_{P+} = 0.89$, $z_{P-} = -0.54$) charge pairing, the pre-compressed simulations result in a single large sustained droplet for the entire duration of sampling whereas the simulations under the regular protocol produced a distribution of smaller droplets. This suggests that the results presented on the ($z_{P+} = 0.89$, $z_{P-} = -0.54$) for simulations at a reduced ion size may not reflect thermodynamic equilibrium.

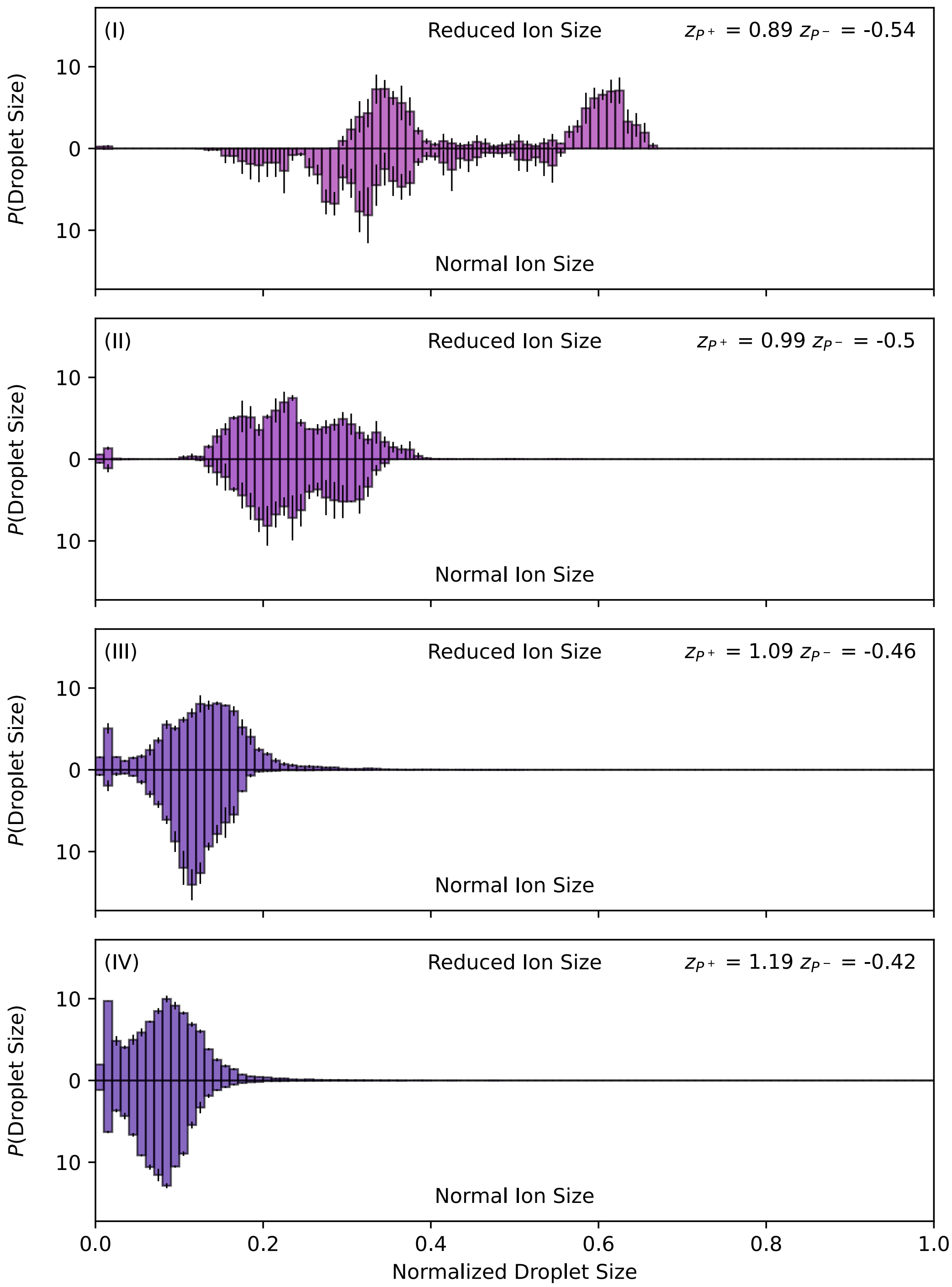

FIG. S12. **Normalized droplet size distributions for reduced ion size versus normal simulations.** Droplet size histograms are presented for four charge pairings—($z_{P+} = 0.89$, $z_{P-} = -0.54$), ($z_{P+} = 0.99$, $z_{P-} = -0.5$), ($z_{P+} = 1.09$, $z_{P-} = -0.46$), and ($z_{P+} = 1.19$, $z_{P-} = -0.42$). For each charge pairing, the results are shown for three simulation replicates run with reduced ($\sigma = 0.1$) and normal ($\sigma = 1.0$) ion sizes. The results are qualitatively similar in both cases; even at a reduced ion size charge asymmetry can still control droplet sizes. Note that droplets of less than 10 polymer chains are excluded from the histogram.

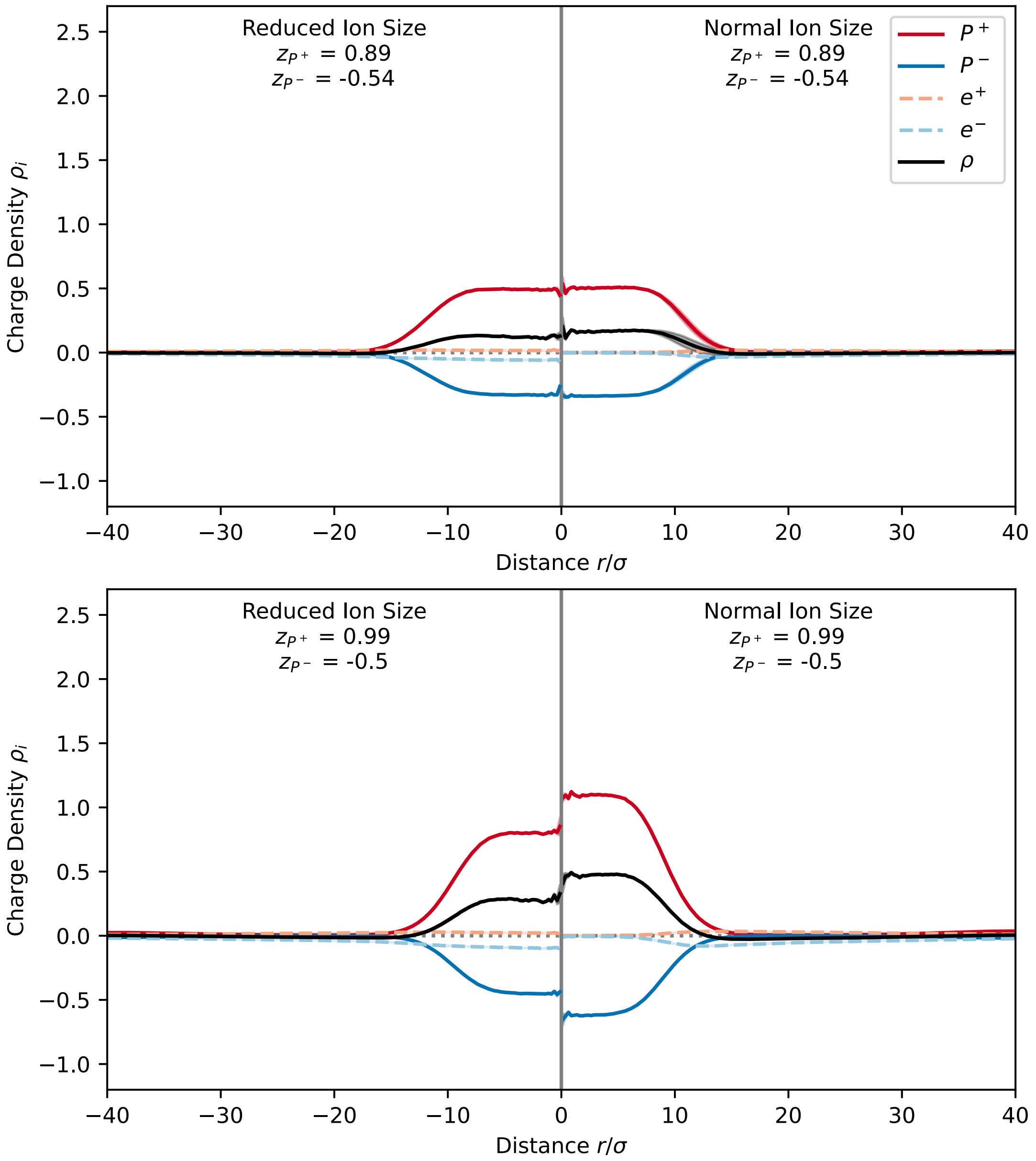

FIG. S13. **Radial charge density profiles for reduced ion size versus normal simulations.** Radial density profiles are presented for the charge pairings ($z_{P+} = 0.89$, $z_{P-} = -0.54$) and ($z_{P+} = 0.99$, $z_{P-} = -0.5$) at both reduced ion size (left) and normal ion size (right) simulations. The results demonstrate that smaller ion sizes lead to more penetration of ions into the dense phase and a lower net charge density in the droplets. However, despite the increased concentration of ions in the dense phase compared to simulations with a normal ion size, the ions still cannot partition to the extent needed to neutralize droplet charge. Thus, the mechanism of size control via charge asymmetry producing droplets with net charges still persists under these conditions.

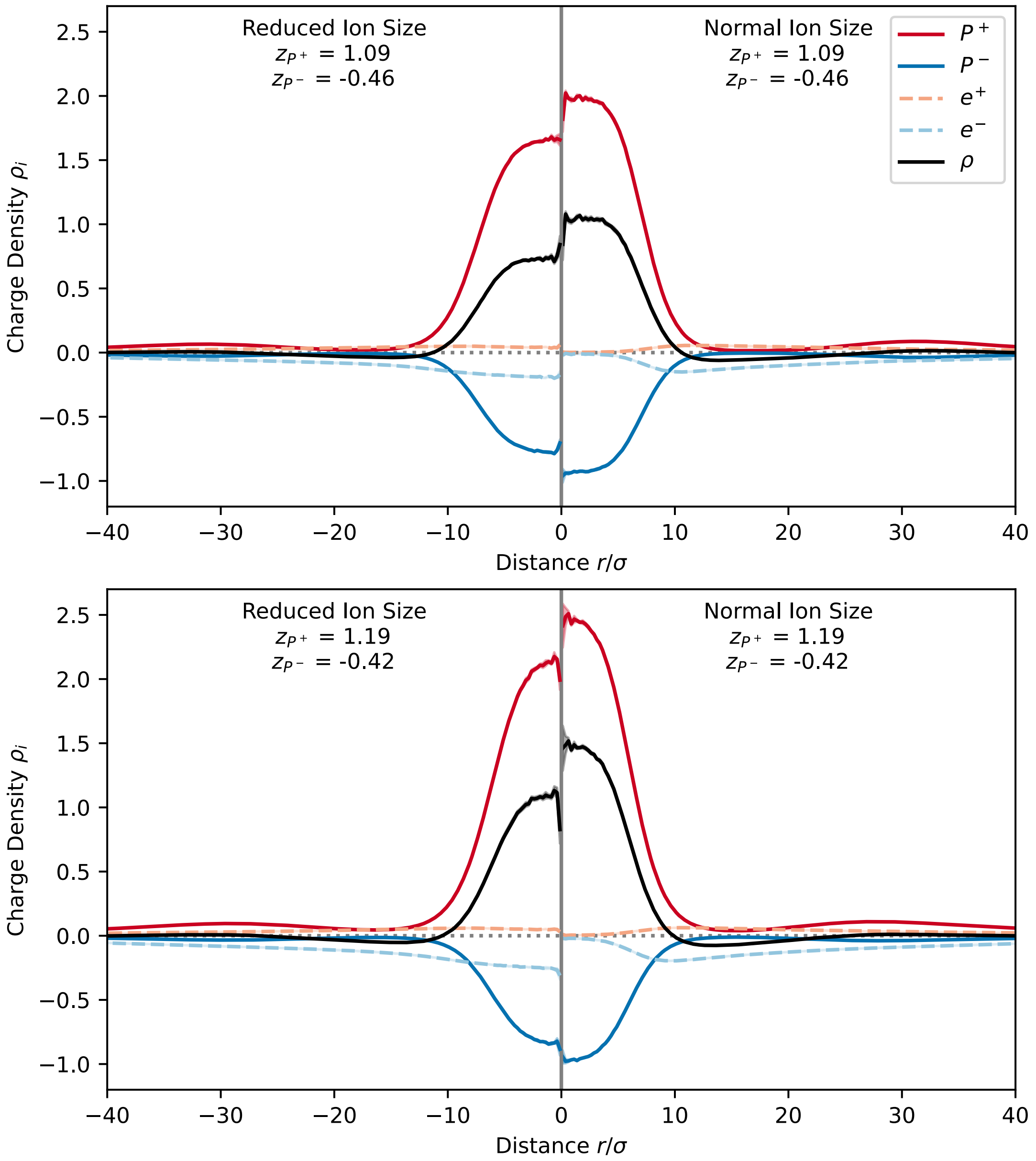

FIG. S14. **Radial charge density profiles for reduced ion size versus normal simulations.** Radial density profiles are presented for the charge pairings ($z_{P+} = 1.09$, $z_{P-} = -0.46$) and ($z_{P+} = 1.19$, $z_{P-} = -0.42$) at both reduced ion size (left) and normal ion size (right) simulations. The results demonstrate that smaller ion sizes lead to more penetration of ions into the dense phase and a lower net charge density in the droplets. However, despite the increased concentration of ions in the dense phase compared to simulations with a normal ion size, the ions still cannot partition to the extent needed to neutralize droplet charge. Thus, the mechanism of size control via charge asymmetry producing droplets with net charges still persists under these conditions.

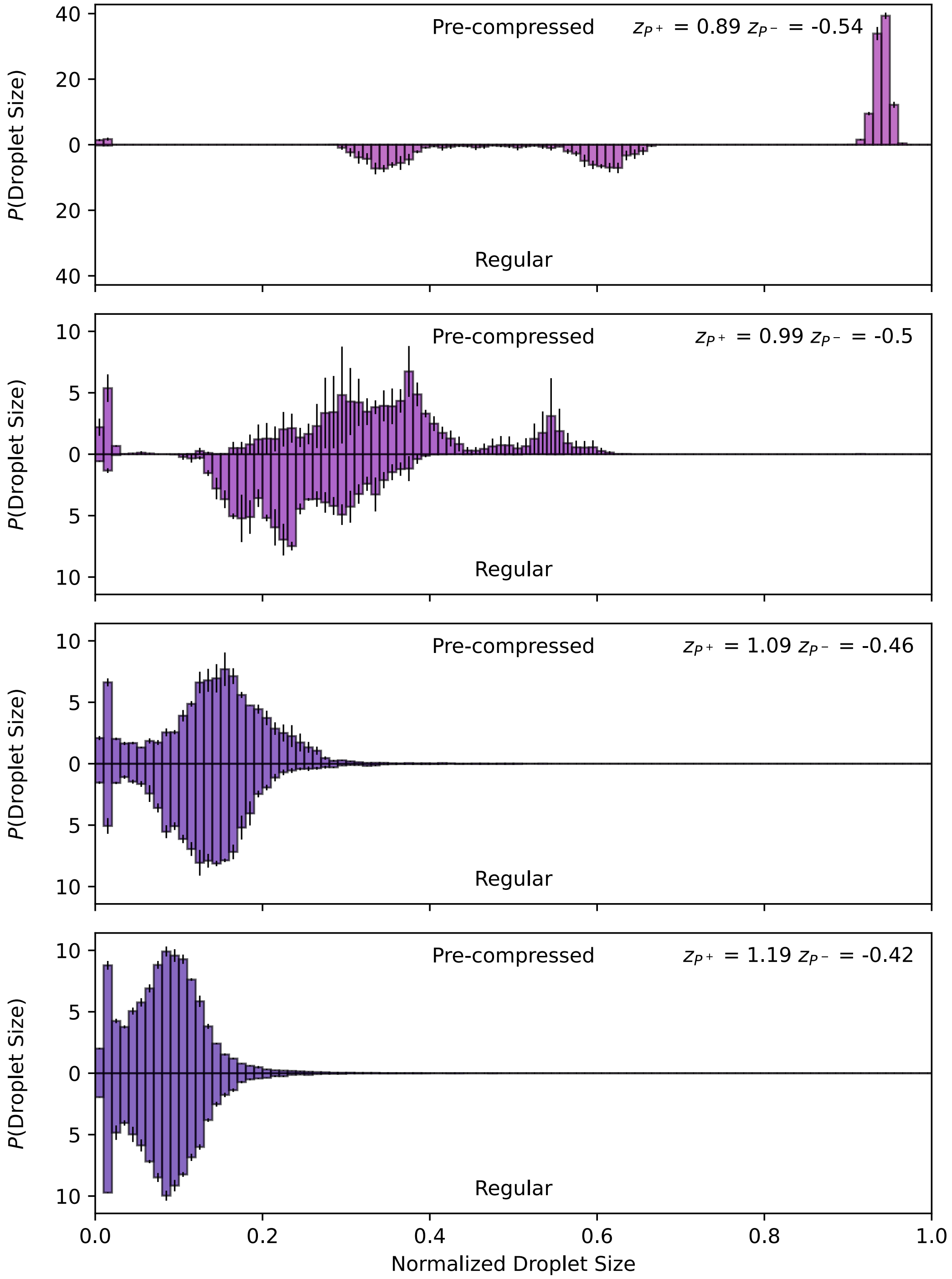

FIG. S15. **Normalized droplet size distributions with reduced ion sizes for both pre-compressed and regular simulations.** Simulations with reduced ion size were performed using the pre-compressed (top) (subsection I C) and the regular (bottom) (subsection I A) simulation procedures. Similarities between the droplet size distributions for the pre-compressed and regular simulations in the charge pairings ($z_{P+} = 0.99$, $z_{P-} = -0.5$), ($z_{P+} = 1.09$, $z_{P-} = -0.46$), and ($z_{P+} = 1.19$, $z_{P-} = -0.42$) indicate that these simulations are likely sampling equilibrium statistics. For the ($z_{P+} = 0.89$, $z_{P-} = -0.54$) charge pairing, discrepancy between the pre-compressed simulations—which shows one large droplet—and the regular simulations—which shows a distribution of smaller droplet sizes—indicates that for this charge pairing at least one of the two cases is not sampling equilibrium statistics. Note that droplets of less than 10 polymer chains are excluded from the histogram.

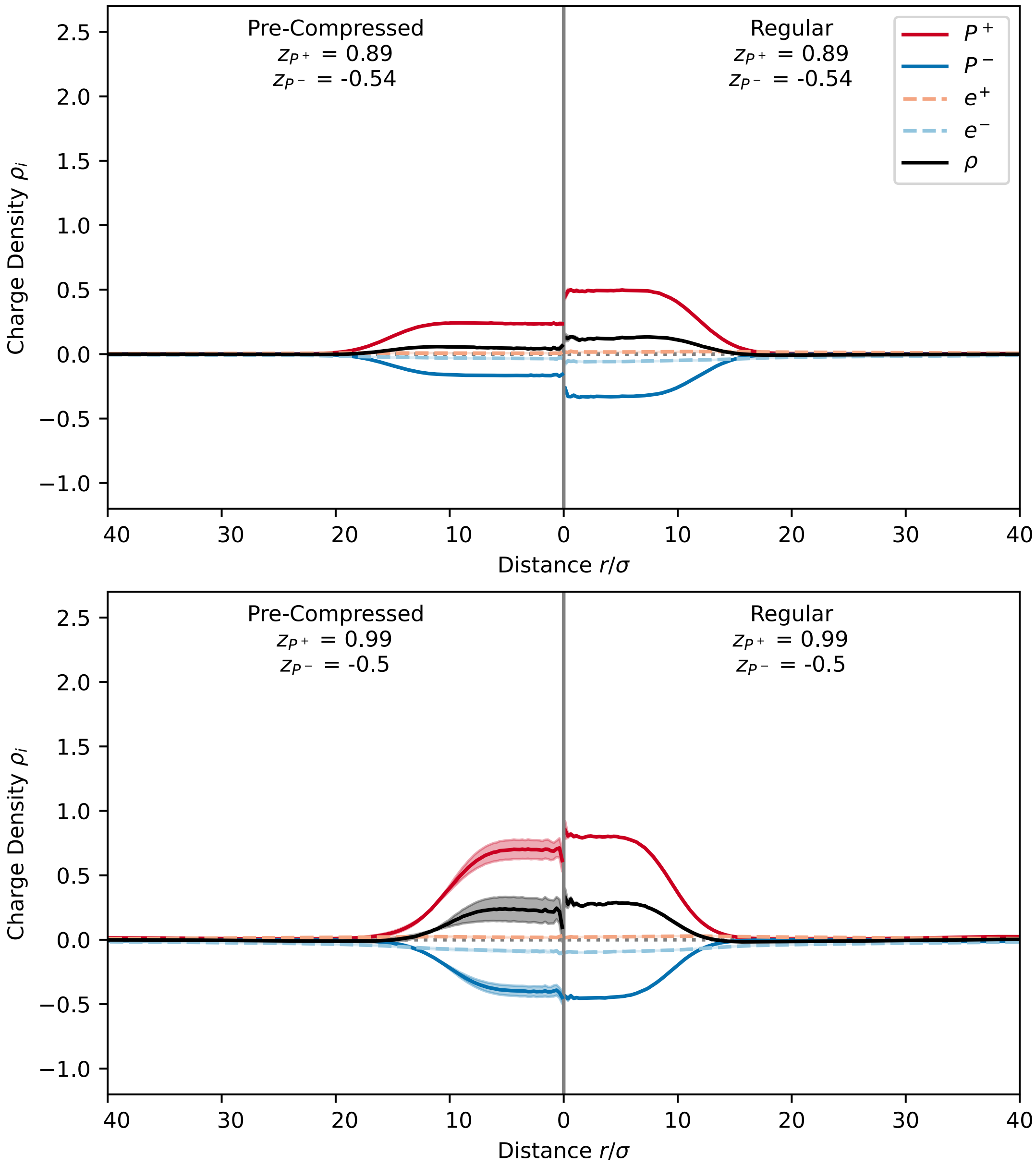

FIG. S16. **Radial charge density profiles with reduced ion size for both pre-compressed and regular simulations.** Simulations with reduced ion size were performed using the pre-compressed (left) (subsection I C) and the regular (right) (subsection I A) simulation procedures. Profiles are shown for the charge pairings of ($z_{P+} = 0.89$, $z_{P-} = -0.54$) and ($z_{P+} = 0.99$, $z_{P-} = -0.5$). The differences between the profiles for ($z_{P+} = 0.89$, $z_{P-} = -0.54$) suggests that at least one of the two simulation protocols is not sampling equilibrium statistics.

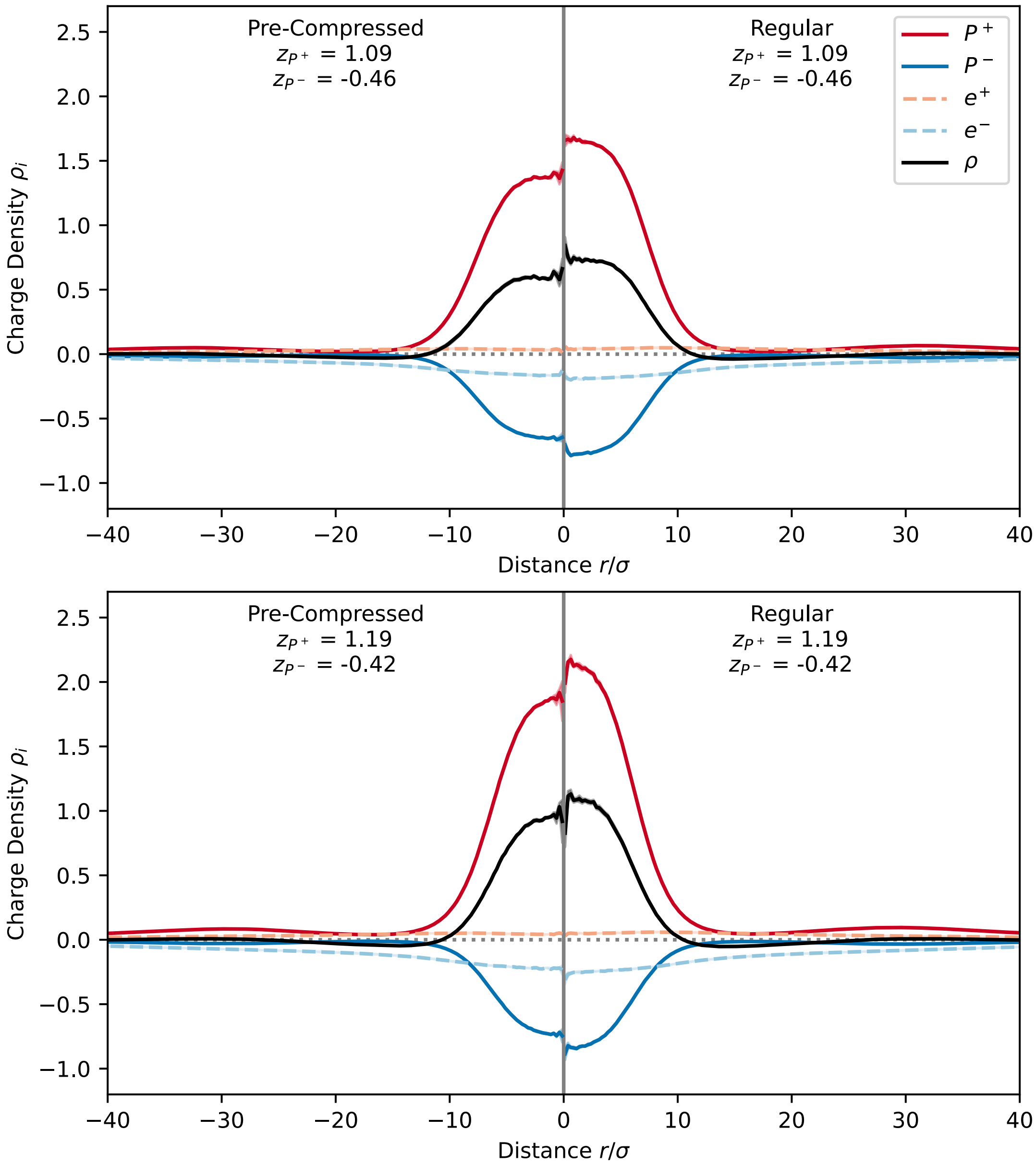

FIG. S17. **Radial charge density profiles with reduced ion size for both pre-compressed and regular simulations.** Simulations with reduced ion size were performed using the pre-compressed (left) (subsection I C) and the regular (right) (subsection I A) simulation procedures. Profiles are shown for the charge pairings of ($z_{P+} = 1.09$, $z_{P-} = -0.46$) and ($z_{P+} = 1.19$, $z_{P-} = -0.42$). The similarity in the profiles indicates that we are sampling equilibrium statistics.

E. Simulations in the overdamped limit

The Langevin thermostat used in the simulations discussed in the main text is underdamped (see the discussion of simulation protocol in subsection I A). This should not change the MD simulation results at equilibrium which can be demonstrated by simulating a subset of the simulations in the overdamped limit. The subset of simulations we choose to resimulate is ($z_{P+} = 0.89$, $z_{P-} = -0.54$), ($z_{P+} = 0.99$, $z_{P-} = -0.5$), ($z_{P+} = 1.09$, $z_{P-} = -0.46$), and ($z_{P+} = 1.19$, $z_{P-} = -0.42$). We run these simulations as discussed in subsection I A with the exception of setting the Langevin thermostat damping constant to a damping of 1.0τ. Below we compare the droplet size histograms (Figure S18) and radial charge density profiles (Figures S19-S20) between the sets of simulations at the two different damping constants. Near identical results are produced for the systems ($z_{P+} = 0.99$, $z_{P-} = -0.5$), ($z_{P+} = 1.09$, $z_{P-} = -0.46$), and ($z_{P+} = 1.19$, $z_{P-} = -0.42$). In the system ($z_{P+} = 0.89$, $z_{P-} = -0.54$) the overdamped simulations result in smaller droplets. This may be due to finite-sized effects at this charge pairing hampering the reproducibility of the distributions produced from the three simulation replicates.

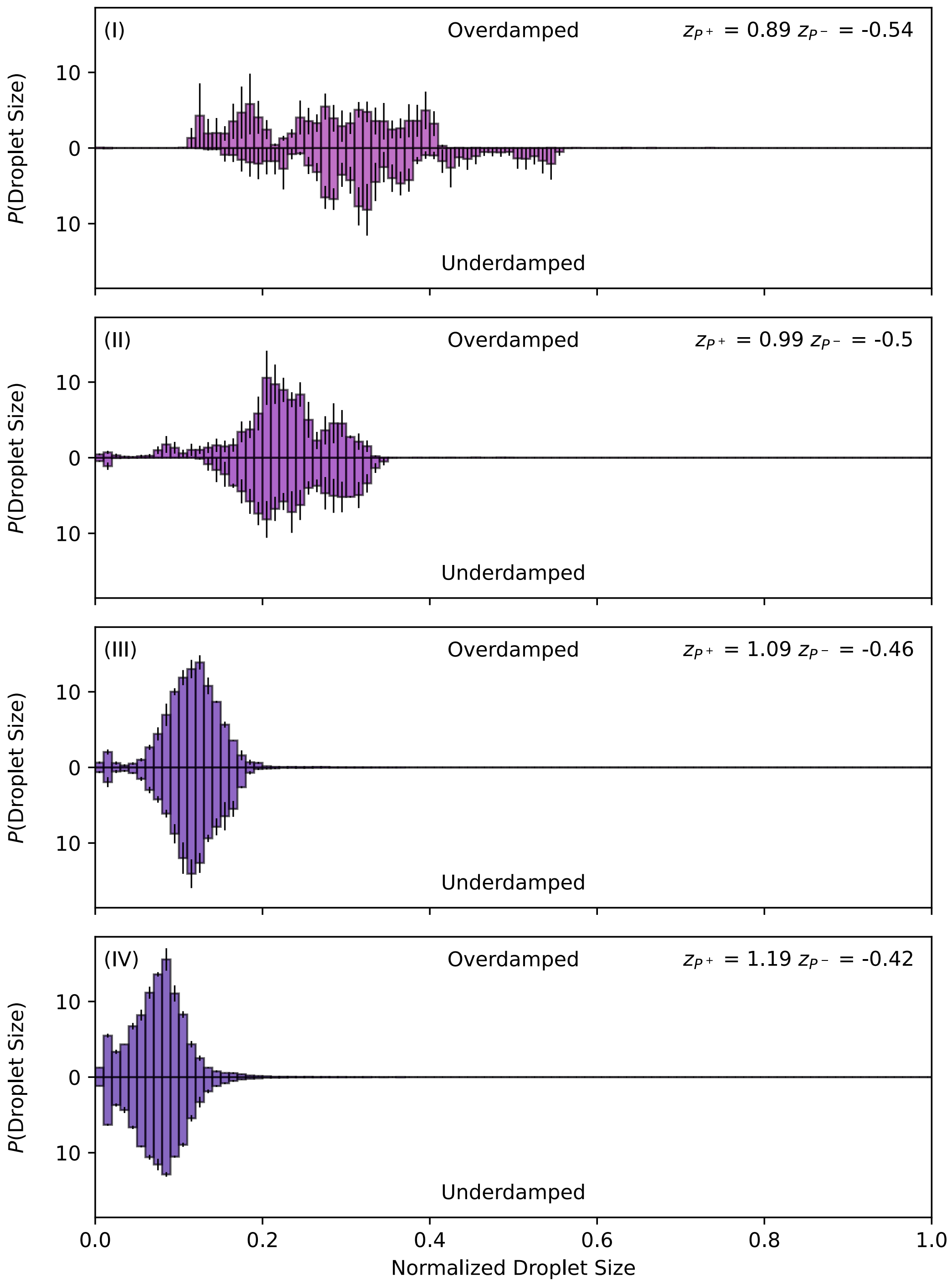

FIG. S18. **Normalized droplet size distributions for overdamped versus underdamped simulations.** Histograms of droplet sizes are presented for simulations run in both the overdamped (top, damping of $\tau = 1.0$) and underdamped (bottom, damping of $\tau = 100.0$) limits for four charge asymmetries ($z_{P+} = 0.89$, $z_{P-} = -0.54$), ($z_{P+} = 0.99$, $z_{P-} = -0.5$), ($z_{P+} = 1.09$, $z_{P-} = -0.46$), and ($z_{P+} = 1.19$, $z_{P-} = -0.42$). The similarities between the droplet size distributions indicates that the results sampled from the simulation protocol described in subsection I A are robust to the thermostat damping constant. The discrepancy in droplet sizes for the ($z_{P+} = 0.89$, $z_{P-} = -0.54$) case may be due to finite sized effects which creates a broad distribution of sizes that are difficult to reproduce quantitatively between two sets of simulation triplicates. Note that droplets of less than 10 polymer chains are excluded from the histogram.

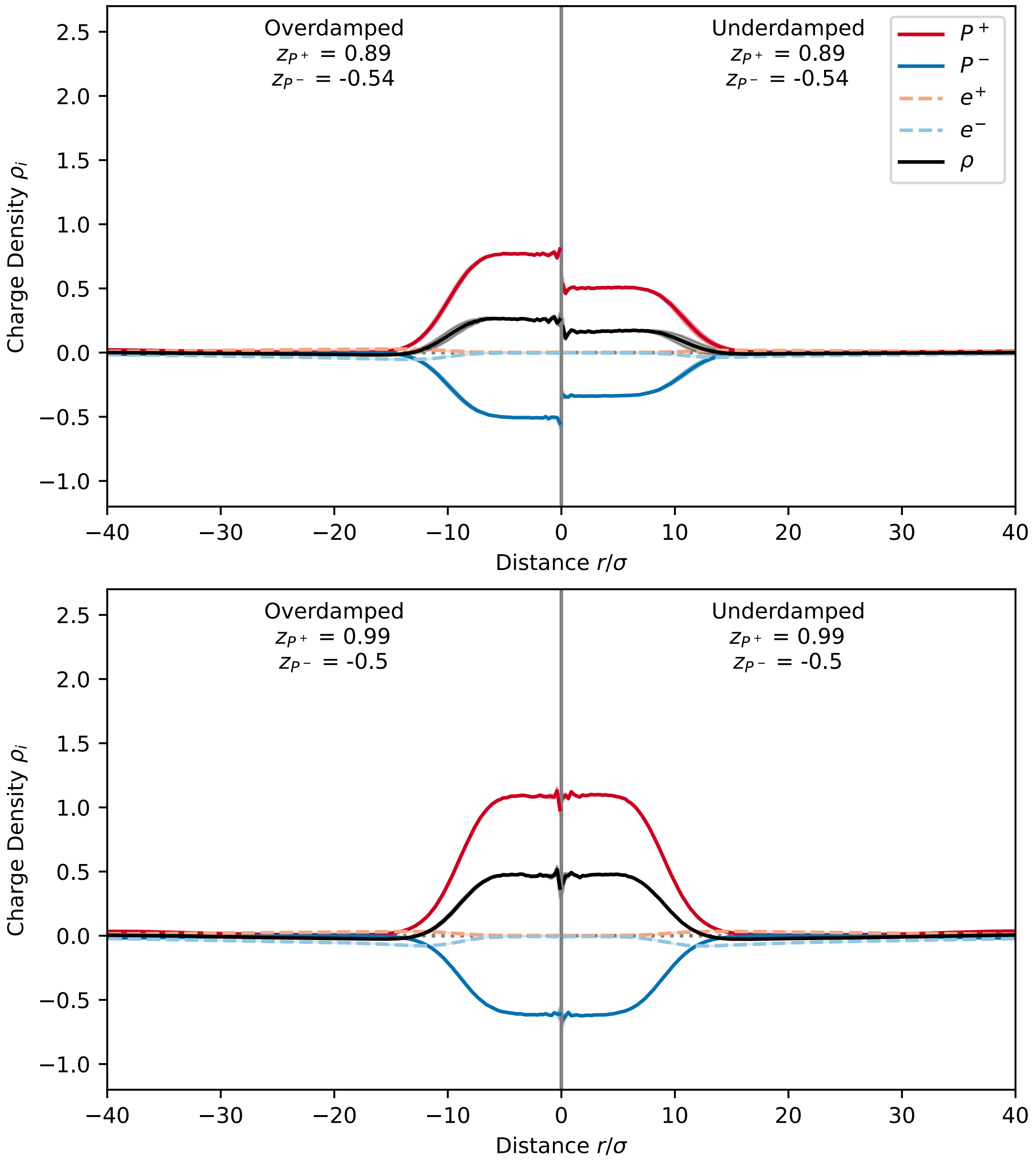

FIG. S19. **Radial charge density profiles for overdamped versus underdamped simulations.** Radial charge density profiles are presented for simulations run in both the overdamped (top, damping of $\tau = 1.0$) and underdamped (bottom, damping of $\tau = 100.0$) limits for the charge asymmetries ($z_{P+} = 0.89$, $z_{P-} = -0.54$) and ($z_{P+} = 0.99$, $z_{P-} = -0.5$). Similarities in the profiles indicate that the results sampled from the simulation protocol described in subsection I A are robust to thermostat damping constant. Discrepancies for the ($z_{P+} = 0.89$, $z_{P-} = -0.54$) system may be attributed to finite sized effects which is also demonstrated by the broad distribution of droplet sizes in Figure S18.

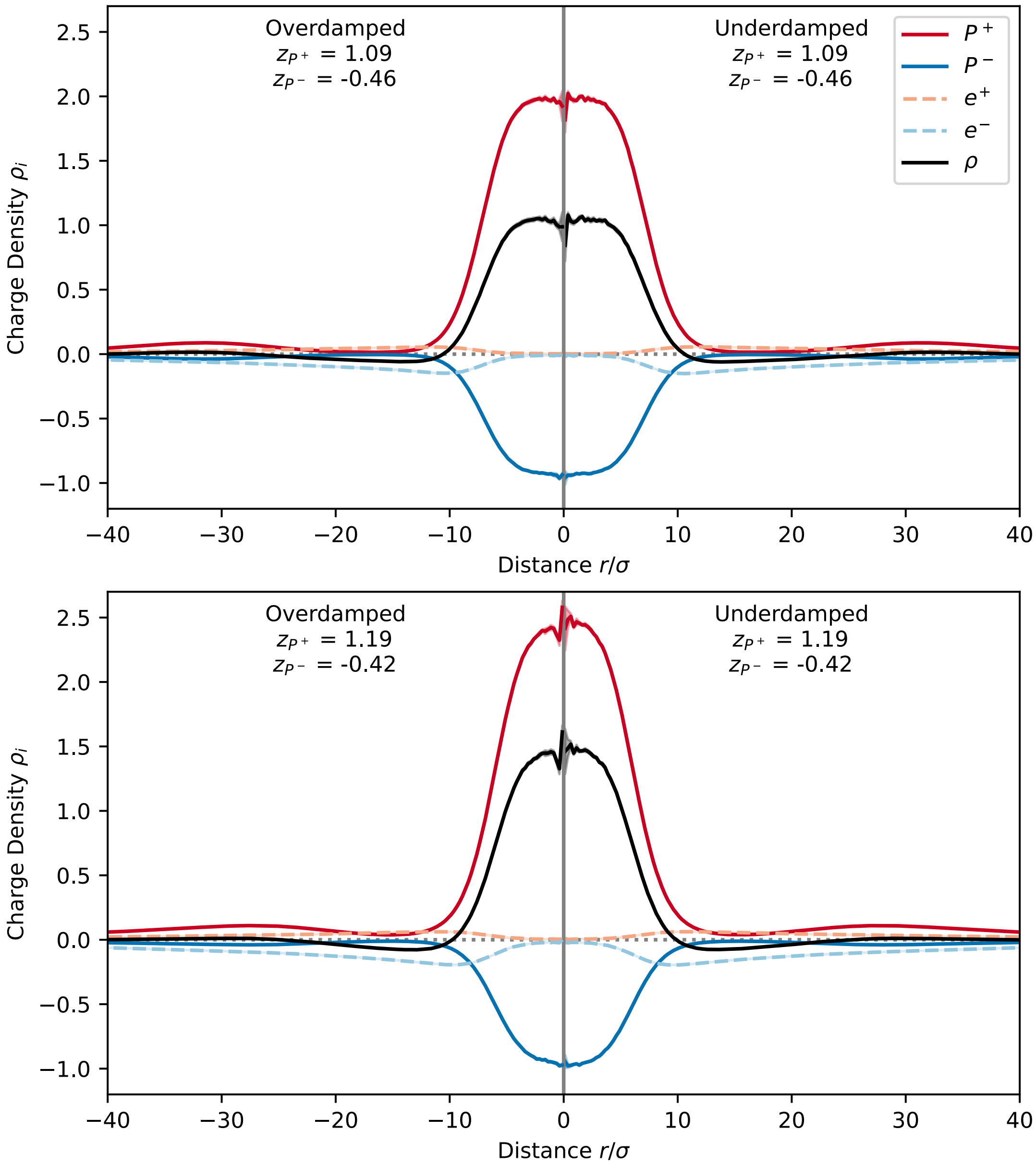

FIG. S20. **Radial charge density profiles for overdamped versus underdamped simulations.** Radial charge density profiles are presented for simulations run in both the overdamped (top, damping of $\tau = 1.0$) and underdamped (bottom, damping of $\tau = 100.0$) limits for the charge asymmetries ($z_{P+} = 1.09$, $z_{P-} = -0.46$) and ($z_{P+} = 1.19$, $z_{P-} = -0.42$). Similarities in the profiles indicate that the results sampled from the simulation protocol described in subsection I A are robust to thermostat damping constant.

F. Simulations at physiological conditions

We performed additional simulations to probe whether the mechanism of condensate size control can appear under conditions that more closely match those seen within cells (i.e., physiological conditions). In particular, this set of simulations match the field theory calculations used in the last part of the main text. Three changes were made to the simulation procedure to accomplish this. First, the dielectric constant was set to $\varepsilon = 1.0$. This set our simulated system's Bjerrum length to be $\ell_{\mathrm{B}} = 1.0\sigma$, setting the overall length scale of our system. Second, ions were set to have a size of $\sigma = 0.62$ whereas the size of a monomer for the charged polymers was varied such that we could explore a $v_{\mathrm{p}} = \{20, 30, 40, 50, 60, 70, 80\}\ell_{\mathrm{B}}^3$. In this way, we could simulate ions having a radius that is smaller than both the Bjerrum length and monomer units on the charged polymers (unlike the simulations done in section I D, where only the latter is true). The charge on each monomer was set such that $\hat{z}_{P+} = 0.5$ and $\hat{z}_{P-} = -0.2$ where $\hat{z}_i = z_i/v_i$ (note that the depiction of the $v_{\mathrm{p}} = 80$ case in Figure 5 of the main text plots the opposite charge asymmetry to align with the discussion in the main text). The only exception is for the $v_{\mathrm{p}} = 80$ case, where two separate systems were run, one with $\hat{z}_{P+} = 0.5$ and one with $\hat{z}_{P+} = 0.2$ (which we refer to as the equal charge or charge symmetric case). Additionally, for all polymer sizes interactions between all species were maintained at $\epsilon_{\mathrm{LJ}} = 1.0$ but cross interaction σ values between species of different sizes were determined using a geometric mixing rule. The attractive Lennard-Jones cutoff was set to be $2.5\sigma_{P+,P-}$ (as is done in section I A) where $\sigma_{P+,P-}$ is the size of the polymer monomers as determined from v_{p}. Further, the cutoff for making droplet size distribution histograms via Ovito clustering analysis [3] is set to $\frac{4}{5}\sigma_{P+,P-}$, identical to what is described in section I A. Third, in order to simulate these systems in a computationally tractable manner, we replicated our system $5 \times 5 \times 5$, resulting in 125 charged polymers of each type. Besides these three changes, the simulation procedure is identical to what is described in section I A.

With this procedure in place, we ran simulations from $v_{\mathrm{p}} = 20$ to $v_{\mathrm{p}} = 80$ to determine whether a patterned phase was discernible under physiological conditions in MD simulations. From the droplet size distributions shown in Figures S21 and S22, it appears that below $v_{\mathrm{p}} = 70$, there is a tailed, unimodal distribution of small droplet sizes. This supports two possible conclusions. The first is that the system is homogeneous and phase separation is not observed. Alternatively, finite-sized effects may dominate in this regime and any phase separated droplet is not readily discernible among small cluster formation and dissolution in the dilute phase. However, at a $v_{\mathrm{p}} = 80$, the distribution of droplet sizes becomes multimodal, supporting a conclusion that at $v_{\mathrm{p}} = 80$ phase separation is certainly occurring. This is presented in Figure S23, where the figure y-axis is adjusted from Figure S22 to highlight the additional mode. In order to determine whether or not $v_{\mathrm{p}} = 80$ represents a patterned phase, we analyzed the radial charge density profile of the $v_{\mathrm{p}} = 80$ case. As is shown in Figure S24, there is no net charge in the largest droplet from the simulation, which is qualitatively similar to the $v_{\mathrm{p}} = 80$ equal charge case (albeit with a smaller radius). Indeed, we additionally made radial charge density profiles of the $v_{\mathrm{p}} = 40$, $v_{\mathrm{p}} = 50$, and $v_{\mathrm{p}} = 60$ cases, averaging the largest droplet across all sampled frames (Figure S25). In all of these cases the largest droplet is charge neutral. Thus, the MD simulations predict that for the $v_{\mathrm{p}} = 80$ case, we should anticipate a homogeneous plus homogeneous phase (i.e., a thermodynamically large dense phase), and if the $v_{\mathrm{p}} = 40$ to 60 cases are phase separated, these should lead to a homogeneous plus homogeneous phases as well. Therefore, under the set of parameters investigated, the MD simulations do not support a conclusion that condensate size control via net charge persists at these conditions. However, we do note that there are small droplets that are produced in all charge asymmetric v_{p} cases analyzed here. These smaller droplets persist in a dilute phase coexisting with a (predicted) thermodynamically large condensate. Importantly, these are not produced under the charge symmetric $v_{\mathrm{p}} = 80$ case underscoring the role of charge asymmetry in creating these small droplets. Thus we still conclude that molecular charge asymmetry dictates the distribution of droplet sizes within cells, although the exact details depend on a variety of factors including ion and polymer sizes, the degree of charge asymmetry, and the strength of short-ranged attractions between polymers.

Finally, we demonstrate that our system has equilibrated by repeating the $v_{\mathrm{p}} = 80$ and $v_{\mathrm{p}} = 80$ equal charge case following the procedure described in section I C. The droplet size distribution histograms presented in Figure S26 and the radial charge density profiles presented in Figure S27 are similar, supporting a conclusion that we have sampled equilibrium statistics in our simulations.

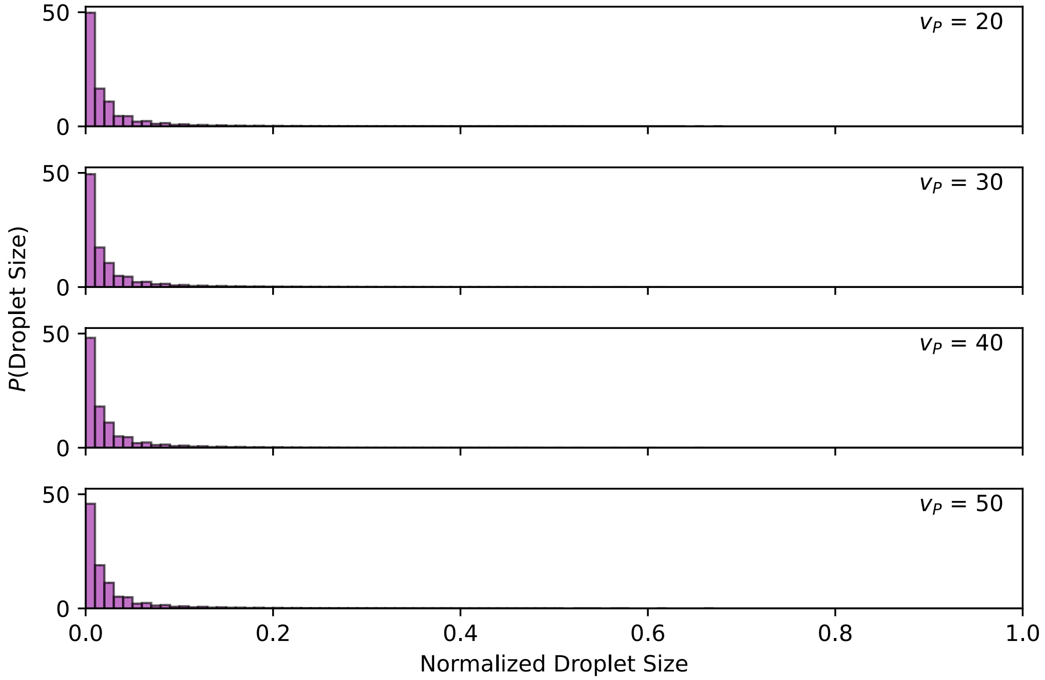

FIG. S21. **Normalized droplet size distributions for the $v_\mathrm{p} = 20$ to $v_\mathrm{p} = 60$ cases.** Four simulation systems, $v_\mathrm{p} = 20$, $v_\mathrm{p} = 30$, $v_\mathrm{p} = 40$, and $v_\mathrm{p} = 50$, are plotted in histograms of normalized droplet sizes. Error bars indicate the standard error across histograms generated from three independent simulation replicates. All histograms show a tailed, unimodal distribution of small droplet sizes which supports a conclusion of either a homogeneous phase or a phase-separated droplet that is indiscernible due to finite-sized effects.

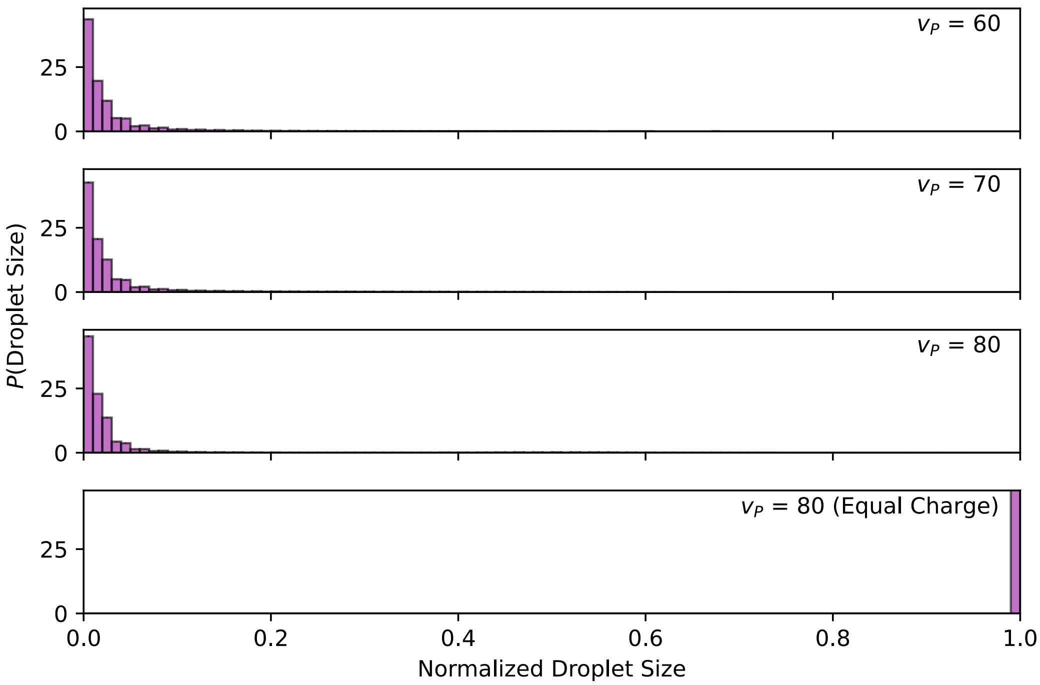

FIG. S22. **Normalized droplet size distributions for the** $v_{\mathrm{p}} = 60$ **to** $v_{\mathrm{p}} = 80$ **(equal charge) cases.** Four simulation systems, $v_{\mathrm{p}} = 60$, $v_{\mathrm{p}} = 70$, $v_{\mathrm{p}} = 80$, and $v_{\mathrm{p}} = 80$ (equal charge), are plotted in histograms of normalized droplet sizes. Error bars indicate the standard error across histograms generated from three independent simulation replicates. The $v_{\mathrm{p}} = 60$ and $v_{\mathrm{p}} = 70$ histograms show a tailed, unimodal distribution which supports an interpretation of either a homogeneous phase or a phase-separated droplet that is indiscernible due to finite-sized effects. The $v_{\mathrm{p}} = 80$ case shows a bimodal droplet distribution, indicating the presence of phase separation. The $v_{\mathrm{p}} = 80$ equal charge case shows one large droplet, also indicating phase separation.

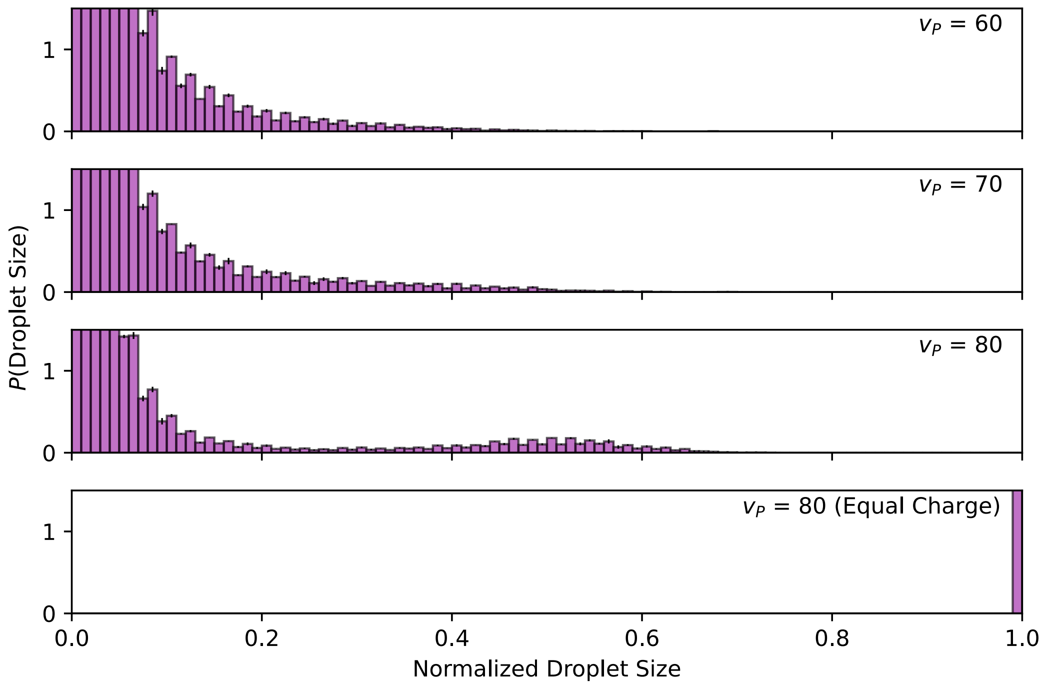

FIG. S23. **Normalized droplet size distributions for the $v_\mathrm{p} = 60$ to $v_\mathrm{p} = 80$ (equal charge) cases.** Four simulation systems, $v_\mathrm{p} = 60$, $v_\mathrm{p} = 70$, $v_\mathrm{p} = 80$, and $v_\mathrm{p} = 80$ (equal charge), are plotted in histograms of normalized droplet sizes. Error bars indicate the standard error across histograms generated from three independent simulation replicates. The $v_\mathrm{p} = 60$ and $v_\mathrm{p} = 70$ histograms show a tailed, unimodal distribution which supports an interpretation of either a homogeneous phase or a phase-separated droplet that is indiscernible due to finite-sized effects. The $v_\mathrm{p} = 80$ case shows a bimodal droplet distribution, indicating the presence of phase separation. The $v_\mathrm{p} = 80$ equal charge case shows one large droplet, also indicating phase separation.

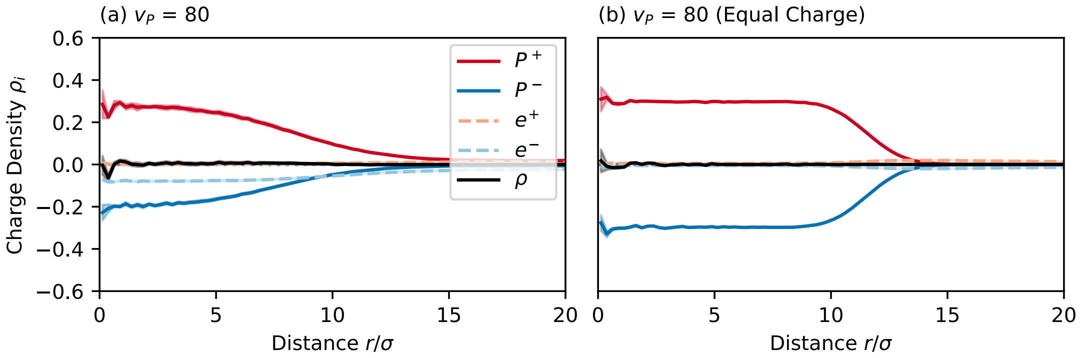

FIG. S24. **Radial charge density profiles for the $v_\mathrm{p} = 80$ and $v_\mathrm{p} = 80$ (equal charge) cases.** There is no net charge density within either profile, which leads to a prediction that both cases would result in a homogeneous plus homogeneous phase (i.e., no droplet size control via net charge).

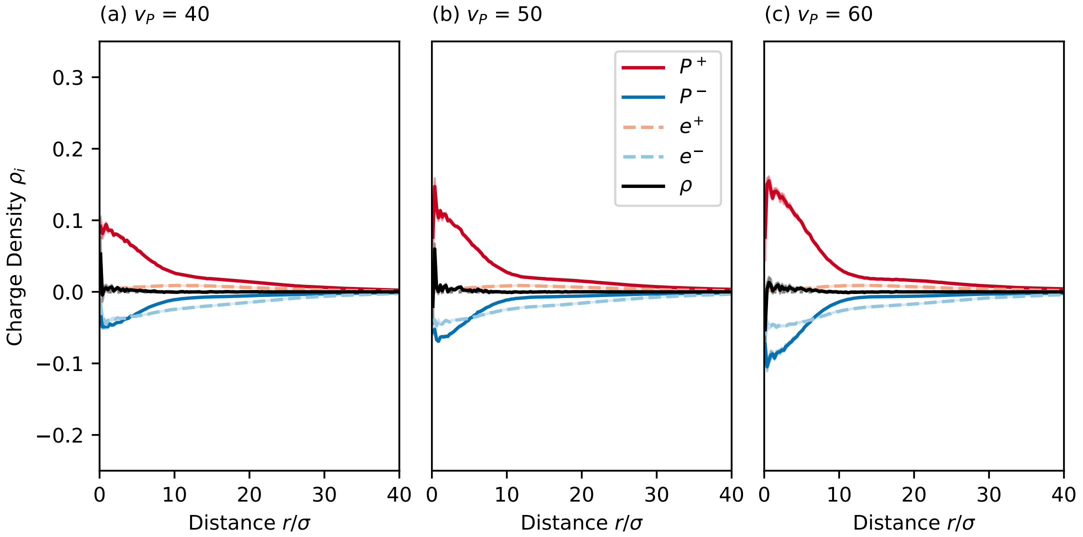

FIG. S25. **Radial charge density profiles for the $v_\mathrm{p} = 40$, $v_\mathrm{p} = 50$, and $v_\mathrm{p} = 60$ cases.** There is no net charge density within any profile, which leads to a prediction that both cases would result in either a homogeneous or a homogeneous plus homogeneous phase (i.e., no droplet size control via net charge).

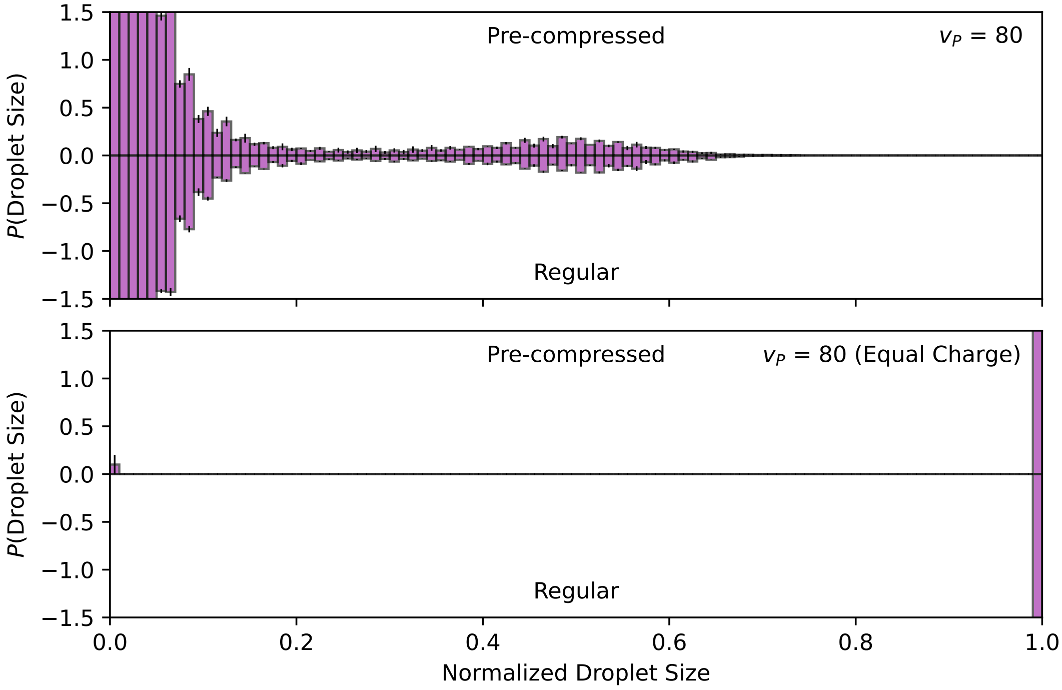

FIG. S26. **Normalized droplet size distributions at $v_\mathrm{p} = 80$ for pre-compressed versus regular simulations.** The similarity between the droplet size distributions obtained following the pre-compressed versus the regular simulation procedures indicates that we are sampling equilibrium statistics.

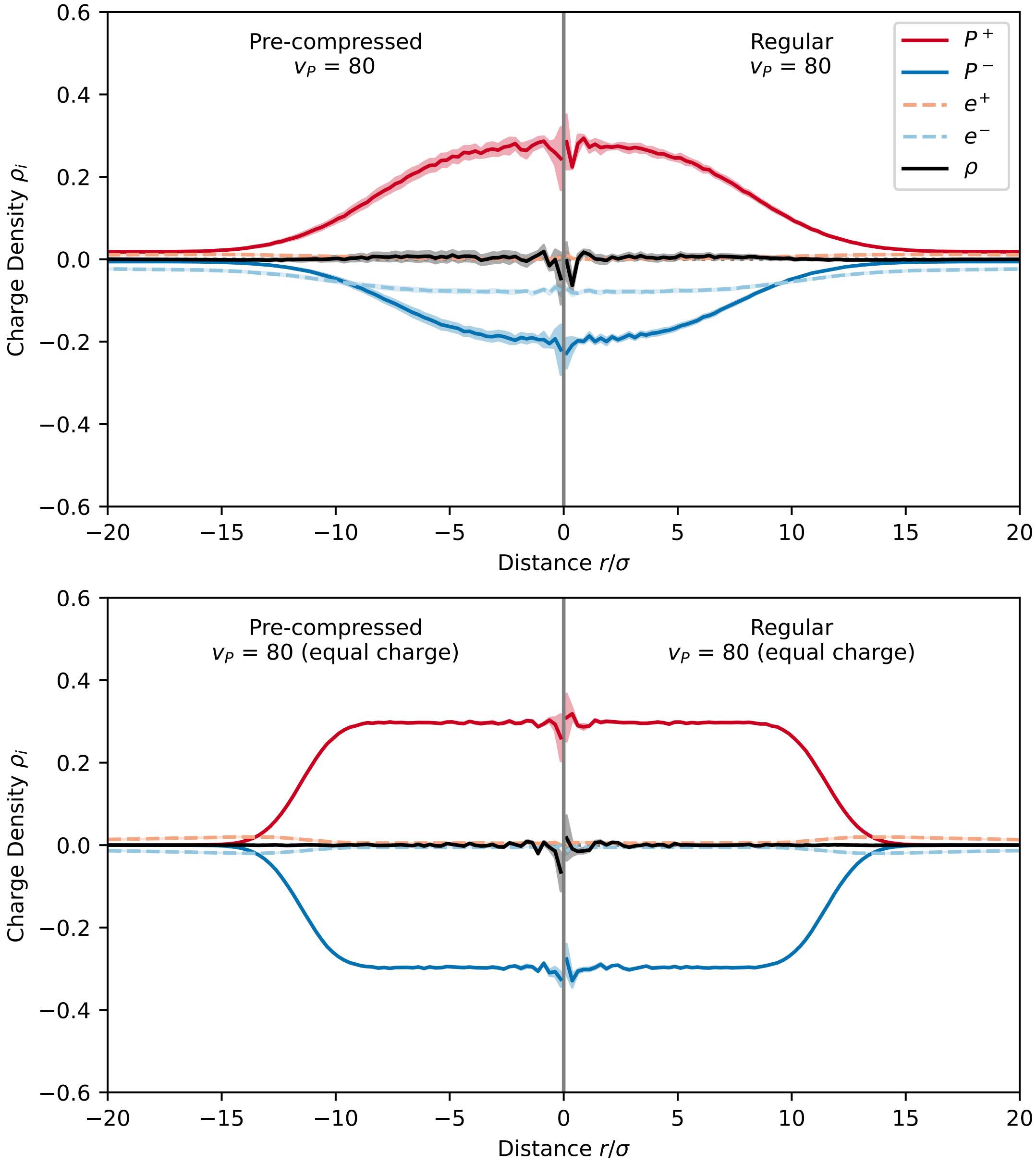

FIG. S27. **Radial charge density profiles at $v_\mathrm{p} = 80$ for pre-compressed versus regular simulations.** The similarity between the radial charge density profiles obtained following the pre-compressed versus the regular simulation procedures indicates that we are sampling equilibrium statistics.

II. DETAILS OF THE FIELD THEORY

We here present mathematical and numerical details of the analysis of the field theory defined by Eqs. (1)–(3) in the main text. The main idea of the numerical minimization to obtain equilibrium states is to separate the entire system into M different phases, which each can either be homogeneous or exhibit periodic patterns. Here, periodic patterns are described by the fields over one period together with the period length scale along each of the d space dimensions. This is discussed in subsection II A, which also includes the derivation that the interfacial energy is balanced by the electrostatic energy in equilibrium; see Eq. (S25). This approach naturally leads to a numerical minimization scheme, which we discuss in subsection II B. We also discuss details of the linear stability analysis of the theory in subsection II C, we quantify the distribution of ions inside and outside the droplets in subsection II D, and we provide additional figures in subsection II G.

A. Free energy minimization

We consider a d-dimensional incompressible, isothermal mixture composed of N_c components with charge numbers z_i for $i = 1, ..., N_\mathrm{c}$. The total free energy including local, interfacial, and long-ranged electrostatic interactions reads

$$F = \frac{k_\mathrm{B}T}{v}\int_V \left[\sum_{i=1}^{N_\mathrm{c}} \frac{\phi_i(\boldsymbol{r})}{l_i}\ln\phi_i(\boldsymbol{r}) + \frac{1}{2}\sum_{i=1}^{N_\mathrm{c}}\sum_{j=1}^{N_\mathrm{c}}\chi_{ij}\phi_i(\boldsymbol{r})\phi_j(\boldsymbol{r}) + \frac{1}{2}\sum_{i=1}^{N_\mathrm{c}}\kappa_i|\nabla\phi_i(\boldsymbol{r})|^2\right]\mathrm{d}\boldsymbol{r} + \int_V\left[-\frac{\varepsilon}{8\pi}|\nabla\psi(\boldsymbol{r})|^2 + \frac{e\psi(\boldsymbol{r})}{v}\sum_{i=1}^{N_\mathrm{c}} z_i\phi_i(\boldsymbol{r})\right]\mathrm{d}\boldsymbol{r} \ . \tag{S6}$$

Using the Bjerrum length $\ell_\mathrm{B} = e^2/(\varepsilon k_\mathrm{B}T)$, $v \to v\ell_\mathrm{B}^3$, $V \to V\ell_\mathrm{B}^3$, $\kappa_i \to \kappa_i\ell_\mathrm{B}^2$, $\nabla \to \nabla/\ell_\mathrm{B}$, and $\psi \to \psi k_\mathrm{B}T/e$, we obtain the dimensionless free energy density $\hat{f} = Fv/(k_\mathrm{B}TV)$,

$$\hat{f} = \frac{1}{V}\int_V\left[\sum_i \frac{\phi_i(\boldsymbol{r})}{l_i}\ln\phi_i(\boldsymbol{r}) + \frac{1}{2}\sum_{ij}\chi_{ij}\phi_i(\boldsymbol{r})\phi_j(\boldsymbol{r}) + \frac{1}{2}\sum_i\kappa_i|\nabla\phi_i(\boldsymbol{r})|^2 - \frac{v}{8\pi}|\nabla\psi(\boldsymbol{r})|^2 + \psi(\boldsymbol{r})\sum_i z_i\phi_i(\boldsymbol{r})\right]\mathrm{d}\boldsymbol{r} \ . \tag{S7}$$

To study the coexistence of different phases, we consider a Gibbs ensemble of M phases, where the fraction of volume of phase β is J_β, implying $\sum_{\beta=1}^{M} J_\beta = 1$. The average free energy density $\bar{f} = \sum_{\beta=1}^{M} J_\beta\hat{f}_\beta$ of the entire system then reads

$$\bar{f} = \sum_{\beta=1}^{M}\frac{J_\beta}{V_\beta}\int_{V_\beta}\left[\sum_i\frac{\phi_i(\beta,\boldsymbol{r})}{l_i}\ln\phi_i(\beta,\boldsymbol{r}) + \frac{1}{2}\sum_{ij}\chi_{ij}\phi_i(\beta,\boldsymbol{r})\phi_j(\beta,\boldsymbol{r}) + \frac{1}{2}\sum_i\kappa_i|\nabla\phi_i(\beta,\boldsymbol{r})|^2 - \frac{v}{8\pi}|\nabla\psi(\beta,\boldsymbol{r})|^2 + \psi(\beta,\boldsymbol{r})\sum_i z_i\phi_i(\beta,\boldsymbol{r})\right]\mathrm{d}\boldsymbol{r} \ .$$

Additionally, there are several constraints, including mass conservation for each species,

$$\bar{\phi}_i = \sum_{\beta=1}^{M}\frac{J_\beta}{V_\beta}\int_{V_\beta}\phi_i(\beta,\boldsymbol{r})\mathrm{d}\boldsymbol{r} \ , \tag{S8}$$

incompressibility everywhere in each phase,

$$\sum_{i=1}^{N_\mathrm{c}}\phi_i(\beta,\boldsymbol{r}) = 1 \ , \tag{S9}$$

and charge neutrality in each phase,

$$\frac{1}{V_\beta}\int_{V_\beta}\sum_{i=1}^{N_\mathrm{c}} z_i\phi_i(\beta,\boldsymbol{r})\mathrm{d}\boldsymbol{r} = 0 \ . \tag{S10}$$

Using these constraints, the equilibrium coexisting states can be obtained by minimizing $\bar{f}$ over $\phi_i(\boldsymbol{r})$, $\psi(\boldsymbol{r})$, J_β, and the vector $\boldsymbol{L}_\beta$ describing the size of the compartment β in all d dimensions, which can be different in each phase. To alleviate the problem of

negative volume fractions during the relaxation dynamics and to conserve the average volume fractions, we replace the logarithms associated with the translational entropies by introducing conjugated fields $\omega_i(\beta,\boldsymbol{r})$, akin to reference [4]. This leads to the extended average free energy density

$$f=\sum_{\beta=1}^{M}\frac{J_\beta}{V_\beta}\int_{V_\beta}\Bigg[-\sum_i\phi_i(\beta,\boldsymbol{r})\omega_i(\beta,\boldsymbol{r})+\frac{1}{2}\sum_{ij}\chi_{ij}\phi_i(\beta,\boldsymbol{r})\phi_j(\beta,\boldsymbol{r})+\frac{1}{2}\sum_i\kappa_i|\nabla\phi_i(\beta,\boldsymbol{r})|^2$$
$$-\frac{v}{8\pi}|\nabla\psi(\beta,\boldsymbol{r})|^2+\psi(\beta,\boldsymbol{r})\sum_i z_i\phi_i(\beta,\boldsymbol{r})+\xi(\beta,\boldsymbol{r})\Big(\sum_j\phi_j(\beta,\boldsymbol{r})-1\Big)\Bigg]\mathrm{d}\boldsymbol{r}-\sum_i\frac{\bar{\phi}_i}{l_i}\ln Q_i$$
$$+\eta\Big(\sum_\beta J_\beta-1\Big)+\sum_\beta J_\beta\zeta(\beta)\left(\frac{1}{V_\beta}\int_{V_\beta}\sum_i z_i\phi_i(\beta,\boldsymbol{r})\mathrm{d}\boldsymbol{r}\right)+\sum_\beta J_\beta C\left(\frac{1}{V_\beta}\int_{V_\beta}\sum_i z_i\phi_i(\beta,\boldsymbol{r})\mathrm{d}\boldsymbol{r}\right)^2, \tag{S11}$$

where

$$Q_i=\sum_\beta J_\beta\frac{1}{V_\beta}\int_{V_\beta}e^{-\omega_i(\beta,\boldsymbol{r})l_i}\mathrm{d}\boldsymbol{r}\ . \tag{S12}$$

Here, $\omega_i(\beta,\boldsymbol{r})$ are the conjugate fields of $\phi_i(\beta,\boldsymbol{r})$, whereas $\xi(\beta,\boldsymbol{r})$, η, and $\zeta(\beta)$ are the Lagrange multipliers for incompressibility, compartment volume conservation, and charge neutrality, respectively. The last term proportional to the constant C is added to guide the convergence toward charge neutrality. This term does not contribute once the system is fully converged since global charge neutrality is fulfilled when Eq. (S8) is obeyed.

The extremum of the free energy density f with respect to $\omega_i(\beta,\boldsymbol{r})$ provides

$$\frac{\delta f}{\delta\omega_i(\beta,\boldsymbol{r})}={V_\beta}^{-1}\left(-\phi_i(\beta,\boldsymbol{r})J_\beta+\bar{\phi}_i\frac{e^{-\omega_i(\beta,\boldsymbol{r})l_i}}{Q_i}J_\beta\right)=0\ , \tag{S13}$$

which gives

$$\phi_i(\beta,\boldsymbol{r})=\bar{\phi}_i\frac{e^{-\omega_i(\beta,\boldsymbol{r})l_i}}{Q_i}\ . \tag{S14}$$

Note that this automatically satisfies material conservation,

$$\sum_{\beta=1}^{M}\frac{J_\beta}{V_\beta}\int_{V_\beta}\phi_i(\beta,\boldsymbol{r})\mathrm{d}\boldsymbol{r}=\bar{\phi}_i\ . \tag{S15}$$

Inserting Eq. (S14) together with the constraints into the free energy density f gives exactly $\bar{f}$ except for a constant offset $-\sum_i\frac{\bar{\phi}_i}{l_i}\ln\bar{\phi}_i$, demonstrating the extremum of f is equivalent to the extremum of $\bar{f}$, which we want to calculate.

The extremum of f with respect to $\phi_i(\beta,\boldsymbol{r})$ provides

$$\omega_i(\beta,\boldsymbol{r})=\sum_j\chi_{ij}\phi_j(\beta,\boldsymbol{r})-\kappa_i\nabla^2\phi_i(\beta,\boldsymbol{r})+\xi(\beta,\boldsymbol{r})+z_i\psi(\beta,\boldsymbol{r})+z_i\zeta(\beta)+2Cz_i\left(\frac{1}{V_\beta}\int_{V_\beta}\sum_j z_j\phi_j(\beta,\boldsymbol{r})\mathrm{d}\boldsymbol{r}\right)\ . \tag{S16}$$

The extremum of f with respect to $\psi(\beta,\boldsymbol{r})$ results in the Poisson's equation of electrostatics,

$$\nabla^2\psi(\beta,\boldsymbol{r})=-\frac{4\pi}{v}\sum_i z_i\phi_i(\beta,\boldsymbol{r})\ . \tag{S17}$$

The extremum of f with respect to J_β provides

$$-\eta=\frac{1}{V_\beta}\int_0^{V_\beta}\Bigg[-\sum_i\phi_i(\beta,\boldsymbol{r})\omega_i(\beta,\boldsymbol{r})+\sum_{ij}\frac{1}{2}\chi_{ij}\phi_i(\beta,\boldsymbol{r})\phi_j(\beta,\boldsymbol{r})+\frac{1}{2}\sum_i\kappa_i|\nabla\phi_i(\beta,\boldsymbol{r})|^2$$
$$-\frac{v}{8\pi}|\nabla\psi(\beta,\boldsymbol{r})|^2+\psi(\beta,\boldsymbol{r})\sum_i z_i\phi_i(\beta,\boldsymbol{r})+\xi(\beta,\boldsymbol{r})(\sum_j\phi_j(\beta,\boldsymbol{r})-1)\Bigg]\mathrm{d}\boldsymbol{r}$$
$$-\sum_i\frac{1}{l_i}\frac{1}{V_\beta}\int_{V_\beta}\phi_i(\beta,\boldsymbol{r})\mathrm{d}\boldsymbol{r}+\zeta(\beta)\frac{1}{V_\beta}\int_{V_\beta}\sum_i z_i\phi_i(\beta,\boldsymbol{r})\mathrm{d}\boldsymbol{r}+C\left(\frac{1}{V_\beta}\int_{V_\beta}\sum_i z_i\phi_i(\beta,\boldsymbol{r})\mathrm{d}\boldsymbol{r}\right)^2\ . \tag{S18}$$

The extremum of f with respect to $\zeta(\beta)$ gives charge neutrality in each compartment,

$$\frac{1}{V_\beta}\int_{V_\beta}\sum_i z_i\phi_i(\beta,\boldsymbol{r})\mathrm{d}\boldsymbol{r}=0\;. \tag{S19}$$

The extremum of f with respect to η and $\xi(\beta,\boldsymbol{r})$ simply gives incompressibility and volume conservation constraints,

$$\sum_i \phi_i(\beta,\boldsymbol{r})=1\;, \tag{S20}$$

and

$$\sum_\beta J_\beta=1\;, \tag{S21}$$

respectively. The extremum of f with respect to ${L_\beta}^j$, the j-th component of $\boldsymbol{L}_\beta$, gives

$$\begin{aligned}\frac{\mathrm{d}f^*}{\mathrm{d}{L_\beta}^j}&=\left.\frac{\partial f}{\partial {L_\beta}^j}\right|_*+\int\sum_i\left.\frac{\delta f}{\delta\phi_i}\right|_*\frac{\partial\phi_i^*}{\partial {L_\beta}^j}\mathrm{d}\boldsymbol{r}+\int\sum_i\left.\frac{\delta f}{\delta w_i}\right|_*\frac{\partial w_i^*}{\partial {L_\beta}^j}\mathrm{d}\boldsymbol{r}+\int\left.\frac{\delta f}{\delta\psi}\right|_*\frac{\partial\psi^*}{\partial {L_\beta}^j}\mathrm{d}\boldsymbol{r}+\int\left.\frac{\delta f}{\delta\xi}\right|_*\frac{\partial\xi^*}{\partial {L_\beta}^j}\mathrm{d}\boldsymbol{r}+...\\&=\left.\frac{\partial f}{\partial {L_\beta}^j}\right|_*\\&=\frac{\partial}{\partial {L_\beta}^j}\left[J_\beta\frac{1}{V_\beta}\int_{V_\beta}\left(\frac{1}{2}\sum_i\kappa_i|\nabla\phi_i(\beta,\boldsymbol{r})|^2-\frac{v}{8\pi}|\nabla\psi(\beta,\boldsymbol{r})|^2\right)\mathrm{d}\boldsymbol{r}\right]_*=0\;,\end{aligned} \tag{S22}$$

where the star denotes that quantities are evaluated for profiles that have been obtained by optimizing over all fields and parameters except ${L_\beta}^j$. In particular, $f^*({L_\beta}^j)$ denotes the associated free energy density, which then only depends on ${L_\beta}^j$. Using $f^j_{\text{int}}={V_\beta}^{-1}\int_{V_\beta}\frac{1}{2}\sum_i\kappa_i|\partial_j\phi_i(\beta,\boldsymbol{r})|^2\mathrm{d}\boldsymbol{r}$ and $f^j_\psi=-{V_\beta}^{-1}\int_{V_\beta}\frac{v}{8\pi}(\partial_j\psi(\beta,\boldsymbol{r}))^2\mathrm{d}\boldsymbol{r}$, we obtain

$$\frac{\mathrm{d}f^*}{\mathrm{d}{L_\beta}^j}=-J_\beta\frac{2}{{L_\beta}^j}(f^j_{\text{int}}+f^j_\psi)=0\;, \tag{S23}$$

which gives

$$f^j_{\text{int}}=-f^j_\psi\;. \tag{S24}$$

Using Poisson's equation and integration by parts, we can express the electrostatic free energy density as

$$f_{\text{el}}=\frac{1}{V_\beta}\int_{V_\beta}\left[-\frac{v}{8\pi}(\nabla\psi(\beta,\boldsymbol{r}))^2+\psi(\beta,\boldsymbol{r})\sum_i z_i\phi_i(\beta,\boldsymbol{r})\right]\mathrm{d}\boldsymbol{r}=-\sum_j f^j_\psi, \tag{S25}$$

and we thus find $f_{\text{el}}=f_{\text{int}}$ for all compartments, where $f_{\text{int}}=\sum_j f^j_{\text{int}}$ is the free energy density associated with interfaces. This indicates that the electrostatic energy is balanced by the interfacial energy in equilibrium. Specifically, in the 1D case, we have

$$\begin{aligned}\frac{\mathrm{d}f}{\mathrm{d}L_\beta}&=-J_\beta\frac{2}{L_\beta}\frac{1}{L_\beta}\int_0^{L_\beta}\left[\frac{1}{2}\sum_i\kappa_i|\partial_x\phi_i(\beta,x)|^2-\frac{v}{8\pi}|\partial_x\psi(\beta,x)|^2\right]\mathrm{d}x\\&=-J_\beta\frac{2}{L_\beta}\frac{1}{L_\beta}\int_0^{L_\beta}\left[-\frac{1}{2}\sum_i\kappa_i\phi_i(\beta,x)\partial_x^2\phi_i(\beta,x)+\frac{v}{8\pi}\psi(\beta,x)\partial_x^2\psi(\beta,x)\right]\mathrm{d}x=0\;.\end{aligned} \tag{S26}$$

In summary, we obtain the self-consistent equations (S12), (S14), (S16)–(S21), and (S23) to determine equilibrium states.

B. Numerical minimization method

We designed an iterative scheme based on the self-consistent equations above, where we update all values of fields and variables based on their current approximated values, so the iteration converges to the free energy minimum. To improve convergence, we extensively use a simple mixing formula to improve numerical stability, i.e., for a generic variable X, we determine its value

X^{new} at the next iteration step as $X^{\text{new}} = X^{\text{old}} + A_X(X^{\text{ideal}} - X^{\text{old}})$, where X^{old} is the current value and X^{ideal} is the value suggested by the iteration scheme. Here, A_X is an empirically determined mixing rate.

In our numerical scheme, we first calculate Q_i using Eq. (S12), and $\phi_i(\beta, \boldsymbol{r})$ using Eq. (S14). We then calculate $\psi(\beta, \boldsymbol{r})$ via

$$\psi(\beta, \boldsymbol{r}) = \mathcal{F}^{-1}\left[-\frac{1}{k^2}\mathcal{F}\left[-\frac{4\pi}{v}\sum_i z_i\phi_i(\beta, \boldsymbol{r})\right]\right] , \tag{S27}$$

where k is the wavenumber of the Fourier transform $\mathcal{F}$ and its inverse $\mathcal{F}^{-1}$. Next, we calculate $\xi(\beta, \boldsymbol{r})$ using Eq. (S16) and the incompressibility condition given by Eq. (S20),

$$\xi(\beta, \boldsymbol{r}) = -\frac{1}{N_\text{c}}\sum_i\left[\sum_j\chi_{ij}\left(\phi_j(\beta, \boldsymbol{r}) + 1 - \sum_k\phi_k(\beta, \boldsymbol{r})\right) - \kappa_i\nabla^2\phi_i(\beta, \boldsymbol{r}) + z_i\psi(\beta, \boldsymbol{r}) + z_i\zeta(\beta)\right.$$
$$\left. + 2Cz_i\left(\frac{1}{V_\beta}\int_{V_\beta}\sum_j z_j\phi_j(\beta, \boldsymbol{r})\mathrm{d}\boldsymbol{r}\right) - \omega_i(\beta, \boldsymbol{r})\right]. \tag{S28}$$

We now use Eq. (S16) to update the ideal part of the new field, $\omega_i^{\text{ideal}}(\beta, \boldsymbol{r})$, via

$$\omega_i^{\text{ideal}}(\beta, \boldsymbol{r}) = \sum_j\chi_{ij}\phi_j(\beta, \boldsymbol{r}) - \kappa_i\nabla^2\phi_i(\beta, \boldsymbol{r}) + z_i\psi(\beta, \boldsymbol{r}) + z_i\zeta(\beta) + \xi(\beta, \boldsymbol{r}) . \tag{S29}$$

To ensure numerical stability, we update the new field $\omega_i^{\text{new}}(\beta, \boldsymbol{r})$ using a mixture of the old and the ideal one together with the kernel $R_i = \kappa_i$,

$$\omega_i^{\text{new}}(\beta, \boldsymbol{r}) = \omega_i(\beta, \boldsymbol{r}) + \mathcal{F}^{-1}\left[\frac{1}{A_\omega^{-1} + R_ik^2}\mathcal{F}\left[\omega_i^{\text{ideal}}(\beta, \boldsymbol{r}) - \omega_i(\beta, \boldsymbol{r})\right]\right] , \tag{S30}$$

where A_ω is an empirical mixing rate, typically around 0.001. In fact, Eq. (S30) is a simple mixing strategy together with a low pass filter, where the iteration is faster for low frequencies while it is slower slower for high frequencies to make the self-consistent iteration scheme more stable [5, 6]. This approach empirically improved the convergence. We next use Eq. (S18) to update J_β. More specifically, using

$$-\eta_\beta = \frac{1}{V_\beta}\int_0^{V_\beta}\left[-\sum_i\phi_i(\beta, \boldsymbol{r})\omega_i(\beta, \boldsymbol{r}) + \sum_{ij}\frac{1}{2}\chi_{ij}\phi_i(\beta, \boldsymbol{r})\phi_j(\beta, \boldsymbol{r}) + \frac{1}{2}\sum_i\kappa_i|\nabla\phi_i(\beta, \boldsymbol{r})|^2\right.$$
$$\left. - \frac{v}{8\pi}|\nabla\psi(\beta, \boldsymbol{r})|^2 + \psi(\beta, \boldsymbol{r})\sum_i z_i\phi_i(\beta, \boldsymbol{r}) + \xi(\beta, \boldsymbol{r})(\sum_j\phi_j(\beta, \boldsymbol{r}) - 1)\right]\mathrm{d}\boldsymbol{r}$$
$$- \sum_i\frac{1}{l_i}\frac{1}{V_\beta}\int_{V_\beta}\phi_i(\beta, \boldsymbol{r})\mathrm{d}\boldsymbol{r} + \zeta(\beta)\frac{1}{V_\beta}\int_{V_\beta}\sum_i z_i\phi_i(\beta, \boldsymbol{r})\mathrm{d}\boldsymbol{r} + C\left(\frac{1}{V_\beta}\int_{V_\beta}\sum_i z_i\phi_i(\beta, \boldsymbol{r})\mathrm{d}\boldsymbol{r}\right)^2 , \tag{S31}$$

and

$$D_\beta = -\sum_{\gamma=1}^{M}\eta_\gamma J_\gamma + \eta_\beta , \tag{S32}$$

we update J_β via

$$J_\beta^{\text{new}} = J_\beta + A_J D_\beta . \tag{S33}$$

Here, A_J is another empirical mixing rate, which is typically set to 0.001. Afterwards, we shift J_β^{new} to satisfy Eq. (S21) which reads $\sum_\beta J_\beta^{\text{new}} = 1$. Next, we update $\zeta(\beta)$ using Eq. (S19),

$$\zeta^{\text{new}}(\beta) = \zeta(\beta) + A_\zeta J_\beta\frac{1}{V_\beta}\int_{V_\beta}\sum_i z_i\phi_i(\beta, \boldsymbol{r})\mathrm{d}\boldsymbol{r} , \tag{S34}$$

where A_ζ is the third mixing rate, which is set to 10. Finally, in 1D, we update L_β using Eq. (S26), via

$$L_\beta^{\text{new}} = L_\beta + A_L J_\beta\frac{2}{L_\beta}\frac{1}{L_\beta}\int_0^{L_\beta}\left[-\frac{1}{2}\sum_i\kappa_i\phi_i(\beta, x)\partial_x^2\phi_i(\beta, x) + \frac{v}{8\pi}\psi(\beta, x)\partial_x^2\psi(\beta, x)\right]\mathrm{d}x , \tag{S35}$$

where A_L is an empirical mixing rate around 10. We use our iterative scheme to find equilibrium states where each phase is described by fields $\phi_i(\boldsymbol{r})$ and $\psi(\boldsymbol{r})$ together with optimized sizes L_β. To produce the results of the main text, we use $N_\text{c} = 5$, assume $M = 2$, and discretize fields at 128 points along the single dimension. We have checked the correctness at selected parameter values using $M = 8$ phases; All convergent results are consistent with those using $M = 2$.

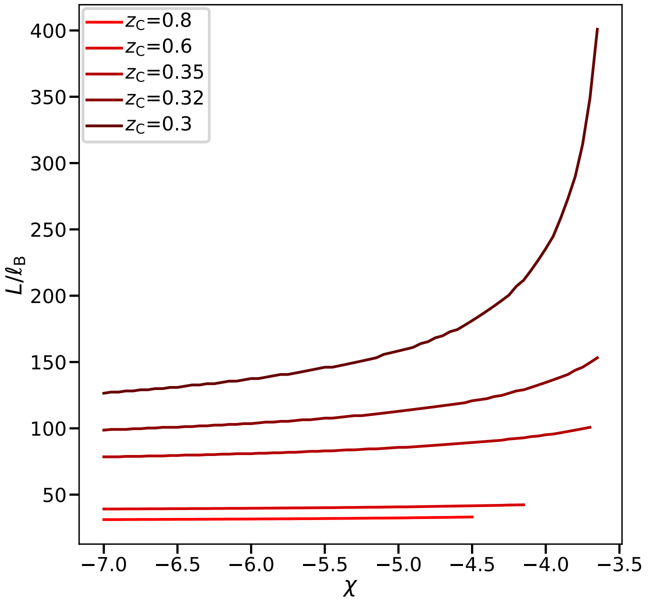

FIG. S28. The most unstable length scale L predicted from linear stability analysis as a function of the interaction parameter χ. Parameters are the same as in Fig. 4 of the main text.

C. Linear stability analysis

We here present the linear stability analysis of the field theory around the uniform state $\phi_i(x) = \bar{\phi}_i$. We perturb $\bar{\phi}_i$ by a small value, setting $\phi_i(x) = \bar{\phi} + \epsilon_i \cos(qx)$, where ϵ_i is the perturbation amplitude and q is the associated wave number. By evaluating Eq. (S7), taking the second-order derivative of $\hat{f}$ with respect to ϵ_i, and then taking the limit $\epsilon_i \to 0$, we obtain the 4×4 Hessian matrix

$$H_{ij} = \left(\frac{1}{\bar{\phi}_i l_i} \delta_{ij} + \frac{1}{\bar{\phi}_\mathrm{S}} \right) + q^2 \kappa_i \delta_{ij} + \frac{4\pi}{v} \frac{z_i z_j}{q^2} \ . \tag{S36}$$

For the homogeneous state to be stable, all eigenvalues λ_i of the Hessian matrix must be positive for all q, which we check numerically. If the homogeneous state is unstable, we numerically calculate the eigenvalues as a function of q and determine the minimal value at $q = q_\mathrm{min}$. The length scale associated with this most unstable mode is $2\pi/q_\mathrm{min}$ and shown in Fig. 3 of the main text. It provides an estimate for the length scale originating when an unstable homogeneous system is prepared, and it sometimes provides a reasonable estimate for the final pattern length scale. To test this, we show the most unstable length scale for small charge asymmetry in Fig. S28, indicating that this length strongly depends on χ especially for weak charge asymmetry, in contrast to the results for the equilibrium length scale presented in the main text.

D. Quantification of ion concentration

One of the main messages of the main text is that ions are unevenly distributed in the patterned phase, with a higher concentration of ions outside the droplet compared to the interior. We here quantify this effect by calculating the average fraction of ions inside droplets and outside droplets separately. Fig. S29 shows that ions are enriched outside droplets (right column) while they are depleted inside droplets (left columns) in the patterned phase. This disparity arises because the short-range attraction between polymers effectively expels ions from the droplet. The strength of this expulsion increases with stronger polymer attractions, (larger $-\chi$) as illustrated in Fig. S30. However, the expulsion weakens as charge asymmetry increases; see Fig. S29.

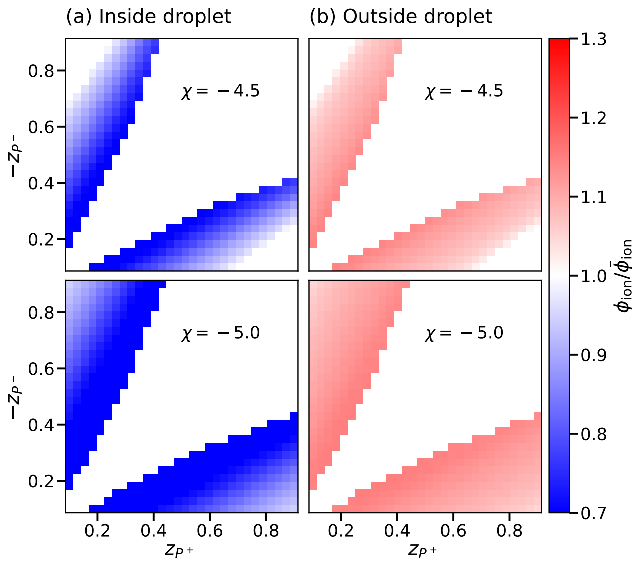

FIG. S29. The relative mean volume fraction of ions, $\phi_{\mathrm{ion}}/\bar{\phi}_{\mathrm{ion}}$, for (a) inside and (b) outside the droplet as a function of the charge numbers of the polymers. Here, ϕ_{ion} is the mean volume fraction of all small ions (e^+ and e^-) inside or outside the droplet, and $\bar{\phi}_{\mathrm{ion}} = \bar{\phi}_{e^+} + \bar{\phi}_{e^-}$. The inside of the droplet is defined as the region where $\phi_{\mathrm{s}} < \frac{1}{2}(\phi_{\mathrm{s}}^{\mathrm{min}} + \phi_{\mathrm{s}}^{\mathrm{max}})$, with $\phi_{\mathrm{s}}^{\mathrm{min}}$ and $\phi_{\mathrm{s}}^{\mathrm{max}}$ representing the minimum and maximum values of the solvent volume fraction, respectively. Conversely, regions where $\phi_{\mathrm{s}} \geq \frac{1}{2}(\phi_{\mathrm{s}}^{\mathrm{min}} + \phi_{\mathrm{s}}^{\mathrm{max}})$ are considered outside the droplet.

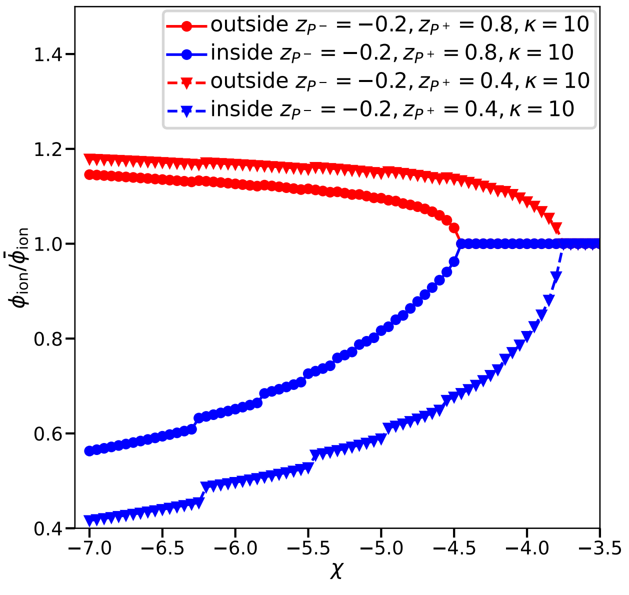

FIG. S30. The relative mean volume fraction of ions, $\phi_{\mathrm{ion}}/\bar{\phi}_{\mathrm{ion}}$, as a function of the interaction strength χ for various charge numbers of the polymers. A stronger short-range attraction (more negative χ) results in a greater expulsion of ions from the droplet.

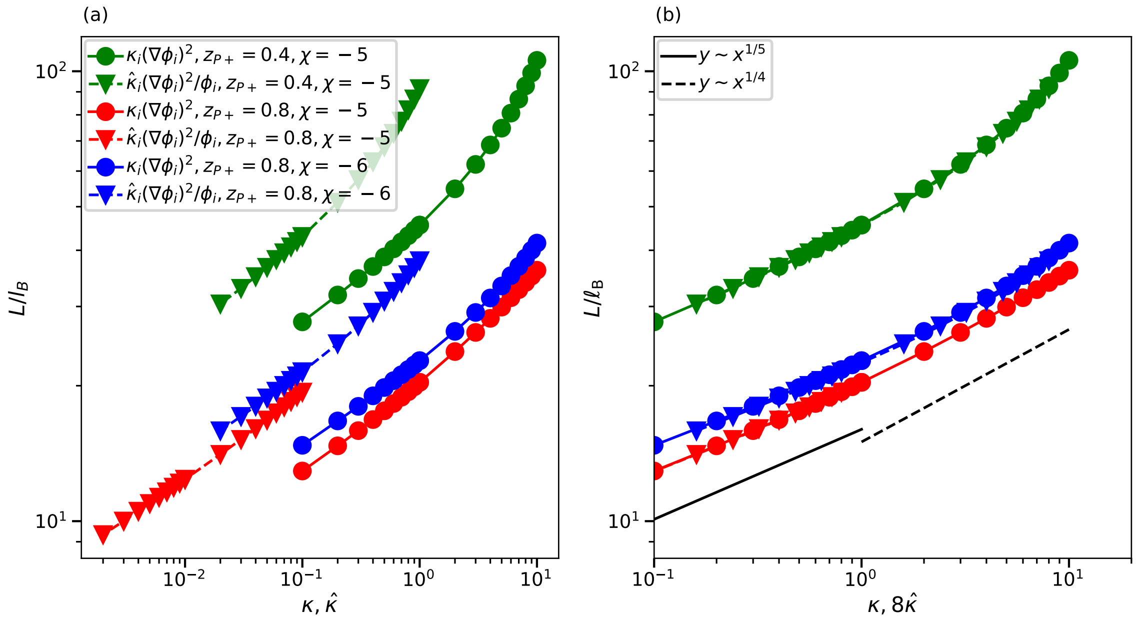

FIG. S31. Effect of incorporating volume fraction into the κ term: while the length scale L changes quantitatively, the qualitative features remain unchanged. $z_{P-} = -0.2$ and other parameters are the same as in Fig. 2 in the main manuscript.

E. No Qualitative Change from Concentration-Dependent Gradient Energy

In literatures [7–9], a concentration-dependent Lifshitz entropy is widely used for polymers rather than the simple Cahn-Hilliard interfacical energy we introduced in the main manuscript. To check if the $\frac{1}{\phi(r)}$ multiplier will qualitatively affect our result about κ, we replace the interfacial energy term

$$\frac{1}{2}\sum_i \kappa_i \left(\nabla\phi_i(\mathbf{r})\right)^2$$

with a concentration-weighted form:

$$\frac{1}{2}\sum_i \hat{\kappa}_i \frac{\left(\nabla\phi_i(\mathbf{r})\right)^2}{\phi_i(\mathbf{r})}.$$

As shown in Fig. S31, this modification results in a quantitative shift in the characteristic length scale L, but does not qualitatively alter the system's behavior or the conclusions drawn from it. Interestingly, by rescaling $\hat{\kappa}$ by approximately a factor of 8, the length scale L closely matches the results presented in Fig. 3 of the main manuscript. This suggests that the relationship between κ and the pattern length scale is preserved, regardless of whether the gradient energy term is concentration-dependent or independent. Moreover, the curves exhibit noticeable curvature, indicating that there is no simple power-law relationship between L and κ. We point out that the electrostatic energy is still balanced by the interfacial energy in equilibrium $f_{\text{el}} = f_{\text{int}}$.

F. Number of chains increases with κ and ion concentration, and decreases with charge asymmetry

For the patterned phase, we define the droplet region as the domain where $\phi_{P+} > \frac{1}{2}\left(\max(\phi_{P+}) + \min(\phi_{P+})\right)$. This allows us to compute the droplet size, denoted by l_{in}. We then calculate the mean volume fraction of species i within the droplet using

$$\phi_i^{\text{in}} = \frac{1}{l_{\text{in}}} \int_{\text{inside}} \phi_i \, dx. \tag{S37}$$

The total amount of polymer chains of species i is proportional to $\phi_i^{\text{in}}\, l_{\text{in}}$, assuming fixed monomer size and chain length.

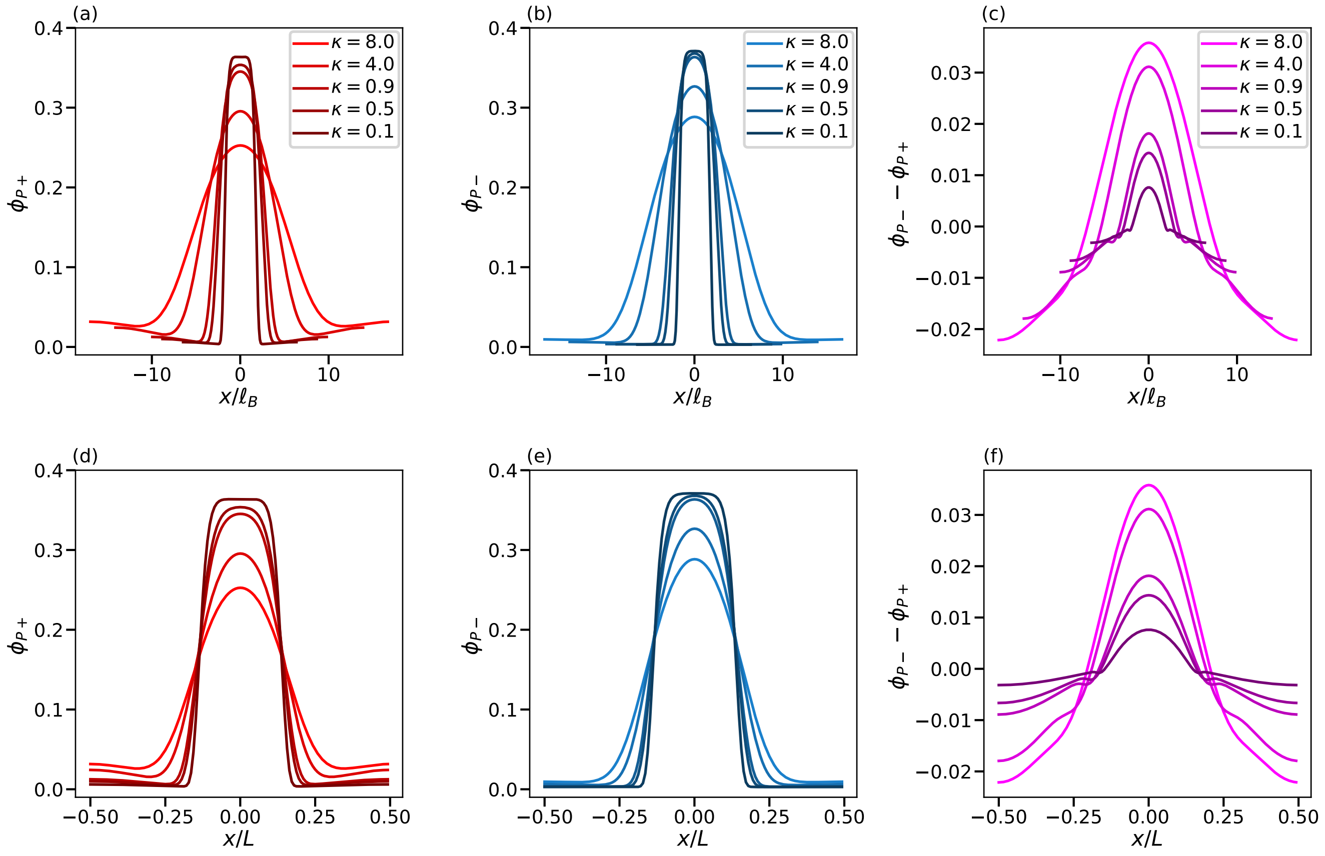

FIG. S32. Profiles for polymers at different κ for $z_{P+} = 0.8$ and $z_{P-} = 0.2$. Panel (c) and (d) show the volume fraction difference between $P+$ and $P-$. Other parameters are the same as in Fig. 2 in the main manuscript.

From Fig. S32 and Fig. S33, we observe that the droplet size l_{in} increases with increasing κ, which is also evident in Fig. S34(a). Although the peak value of ϕ_{P+} decreases with increasing κ [see Fig. S32(a) and Fig. S33(a)], the total polymer content $\phi_i^{\text{in}}\, l_{\text{in}}$ increases with κ [Fig. S34(b)]. This indicates that more polymers accumulate within the droplet as κ increases. Furthermore, the disparity in the number of chains between the two polymer species also grows with κ, as shown in Fig. S34(c). Similary, the droplet size l_{in}, the volume of polymers, and the disparity in the number of chains between the two polymer species all increase with increasing the ion volume fractions $\bar{\phi}_{\text{ion}}$ [Fig. S35]. In contrast, increasing the charge asymmetry reduces both ϕ_{P+}^{in}, as already shown by the amplitude decrease in Fig. 3(b) of the main text, and the droplet size l_{in}, as illustrated in Fig. S36. Consequently, the total number of chains, $\phi_{P+}^{\text{in}} l_{\text{in}}$ and $\phi_{P-}^{\text{in}} l_{\text{in}}$, decreases with charge asymmetry [Fig. S36(a) and (b)]. The difference in chain number between the two polymer species also decreases with increasing charge asymmetry [Fig. S36(c)].

G. Additional result figures

Figure S37 shows phase diagram of states, pattern periods, amplitudes, and electrostatic energy for $\chi = -4$ and $\chi = -7$, analogous to Fig. 3(b) in the main text. Figures S38 and S39 provide additional details for states depicted in Fig. 2 of the main text. Figure S40 shows a typical profile near the continuous transition. The nature of this transition can be investigate from the dependence of the amplitude of the volume fraction of the negatively-charged polymer P_- as a function of χ; see Fig. S41. The different contributions to the free energy are shown in Fig. S42. The last two columns, in particular, demonstrate that the interfacial energy F_{int} is equal to the electrostatic energy F_{el}.

To check whether dimensionality matters, we also obtain the patterned phase by solving the two-dimensional mean-field theory equations. We show the profiles of volume fractions in Fig. S43 and charge densities in Fig. S44, corresponding to the same states as in Fig. 2 of the main text. The 2D disk patterns qualitatively agree with the 1D results; Notably, we also observe a dip inside the droplet at $z_{P+} = 0.4$. Given the low total volume fractions of polymers, $\phi_{P+} + \phi_{P-} = 0.2$, we expect spheres, rather

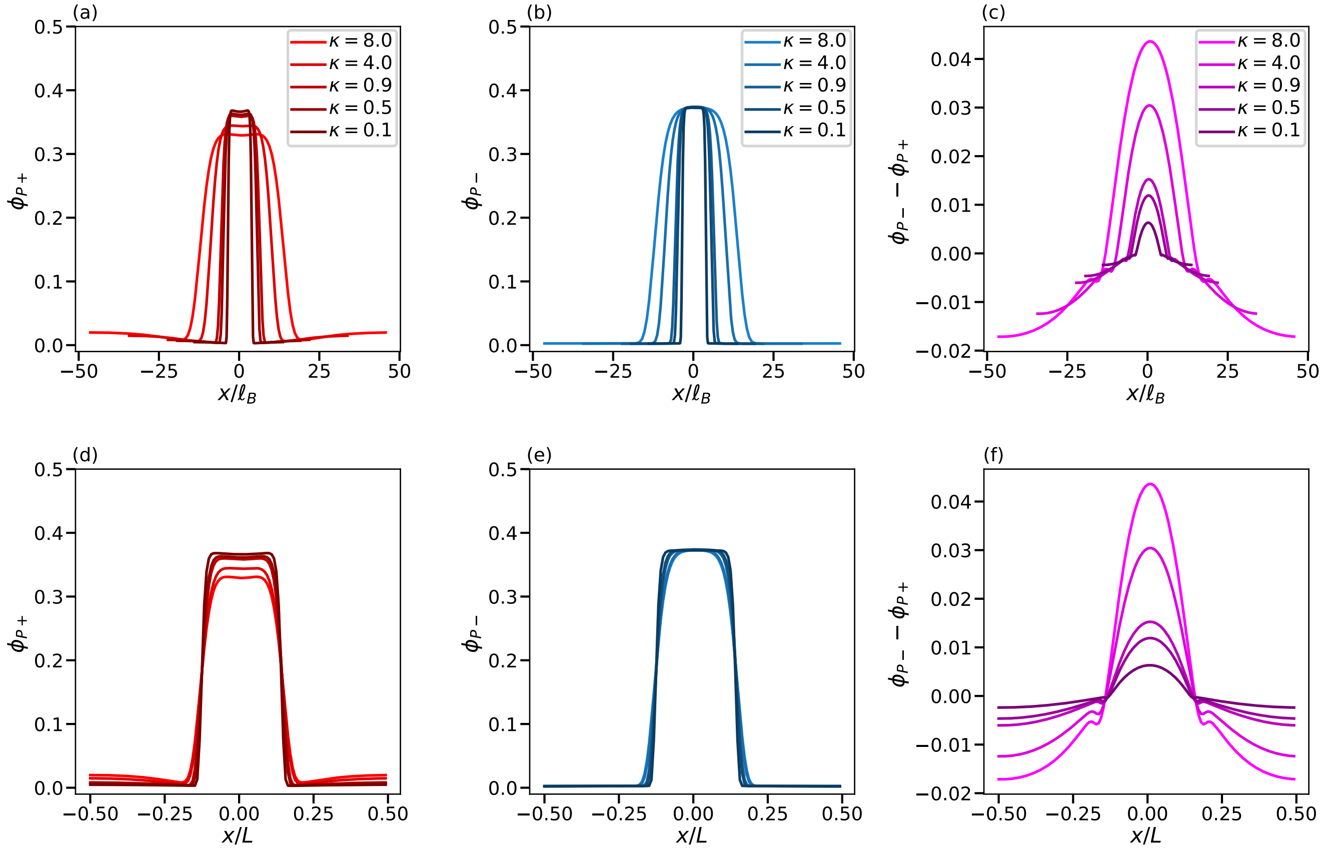

FIG. S33. Profiles for polymers at different κ for $z_{P+} = 0.4$ and $z_{P-} = 0.2$. Panel (c) and (d) show the volume fraction difference between $P+$ and $P-$. Other parameters are the same as in Fig. 2 in the main manuscript.

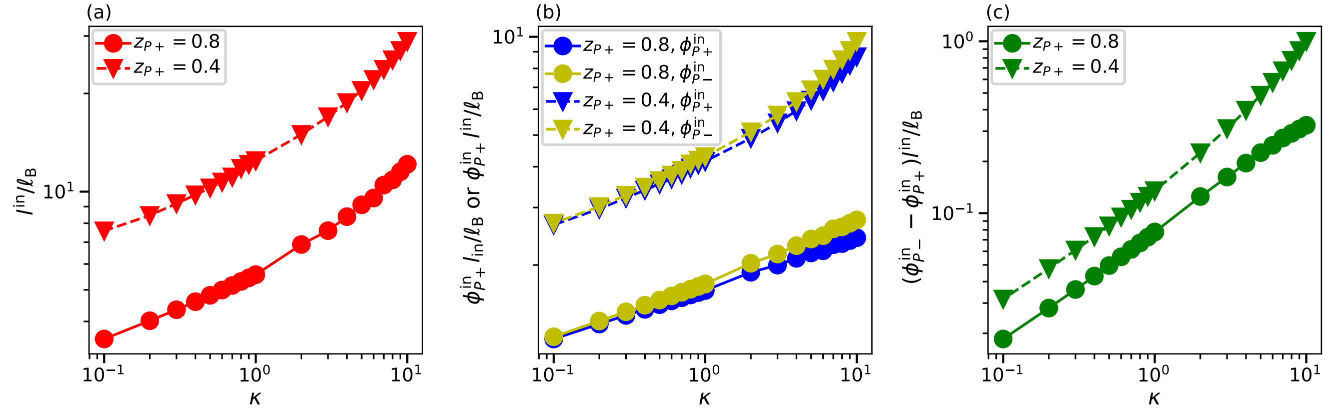

FIG. S34. Number of chains increases with parameter κ. (a) Droplet size $l_{\rm in}$ defined as the length of the region where $\phi_{P+} > (\max(\phi_{P+}) + \min(\phi_{P+}))/2$. (b) The volume of polymers inside the droplet where $\phi^{\rm in}_{P+} = \frac{1}{l_{\rm in}} \int_{\rm inside} \phi_{P+} dx$ ($\phi^{\rm in}_{P-} = \frac{1}{l_{\rm in}} \int_{\rm inside} \phi_{P-} dx$) for positively (negatively) charged polymers. (c) Volume difference between P_+ and P_- within droplet. All other parameters are identical to those used in Fig. 2 of the main manuscript.

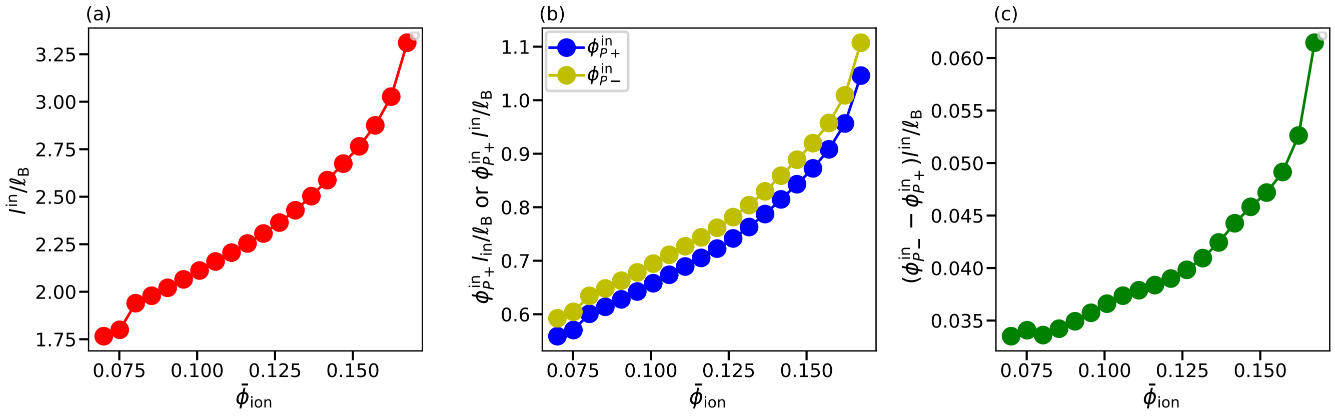

FIG. S35. Number of chains increases with ion volume fractions. (a) Droplet size $l_{\rm in}$ defined as the length of the region where $\phi_{P+} > (\max(\phi_{P+}) + \min(\phi_{P+}))/2$. (b) The volume of polymers inside the droplet where $\phi^{\rm in}_{P+} = \frac{1}{l_{\rm in}} \int_{\rm inside} \phi_{P+} dx$ ($\phi^{\rm in}_{P-} = \frac{1}{l_{\rm in}} \int_{\rm inside} \phi_{P-} dx$) for positively (negatively) charged polymers. (c) Volume difference between P_+ and P_- within droplet. Parameter $\chi = -5$. All other parameters are identical to those used in Fig. 5(b) of the main manuscript.

than lamellar or cylindrical structures, will form in 3D within our parameter regime.

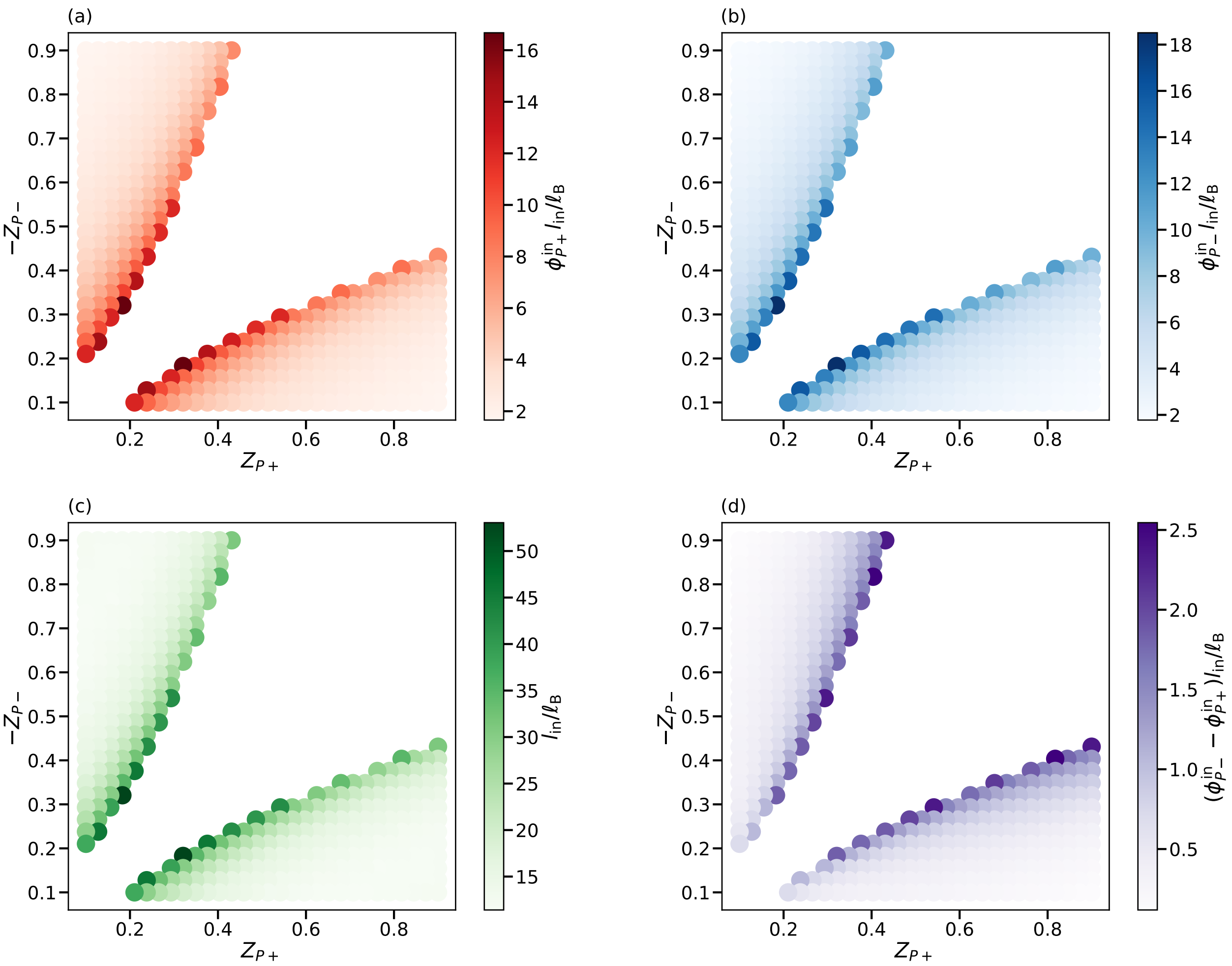

FIG. S36. Number of chains decrease with charge asymmetry. (a) The volume of positively charged polymers inside the droplet. (b) The volume of negatively charged polymers inside the droplet. (c) Droplet size l_{in} defined as the length of the region where $\phi_{P+} > (\max(\phi_{P+}) + \min(\phi_{P+}))/2$. (d) Volume difference between P_+ and P_- within droplet. All other parameters are identical to those used in Fig. 2 of the main manuscript.

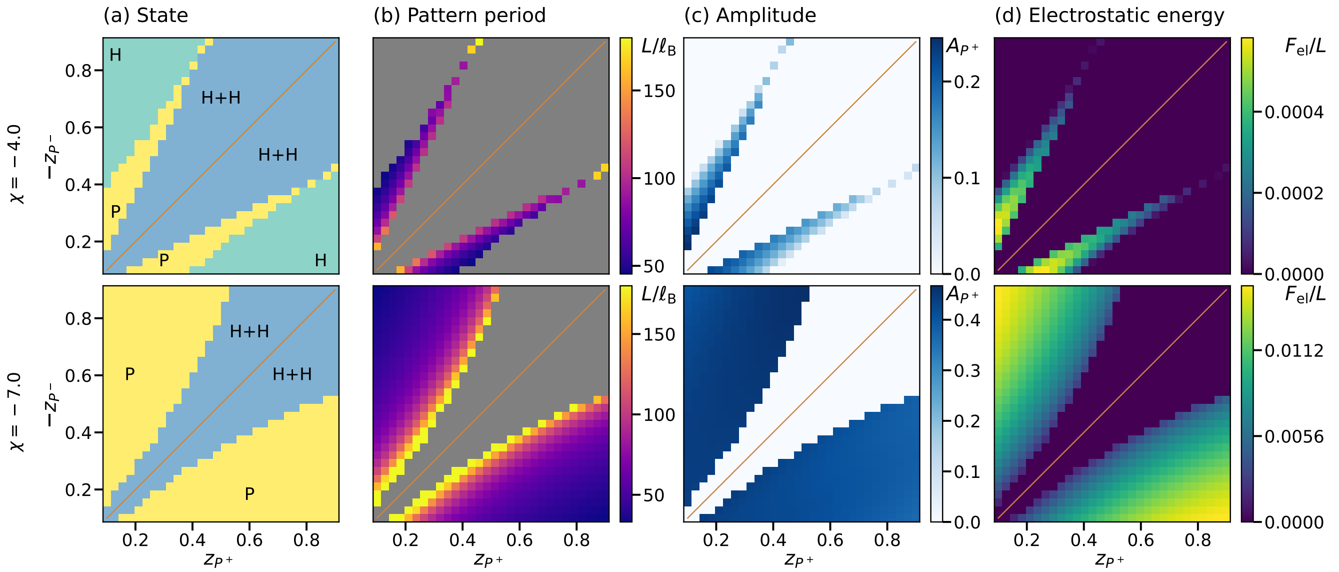

FIG. S37. Results analogous to Fig. 3 in the main text for $\chi = -4$ (upper row) and $\chi = -7$ (lower row). (a) Phase diagram as a function of the charge numbers z_{P+} and z_{P-} of the polymers revealing parameter regions with the coexistence of two homogeneous phases (H+H), patterned phases (P), a single homogeneous phase (H). (b) Period L in the patterned phase corresponding to panel a. (c) Amplitude $A_{P+} = \max(\phi_{P+}) - \min(\phi_{P+})$ corresponding to panel a. (d) Electrostatic energy F_{el} corresponding to panel a.

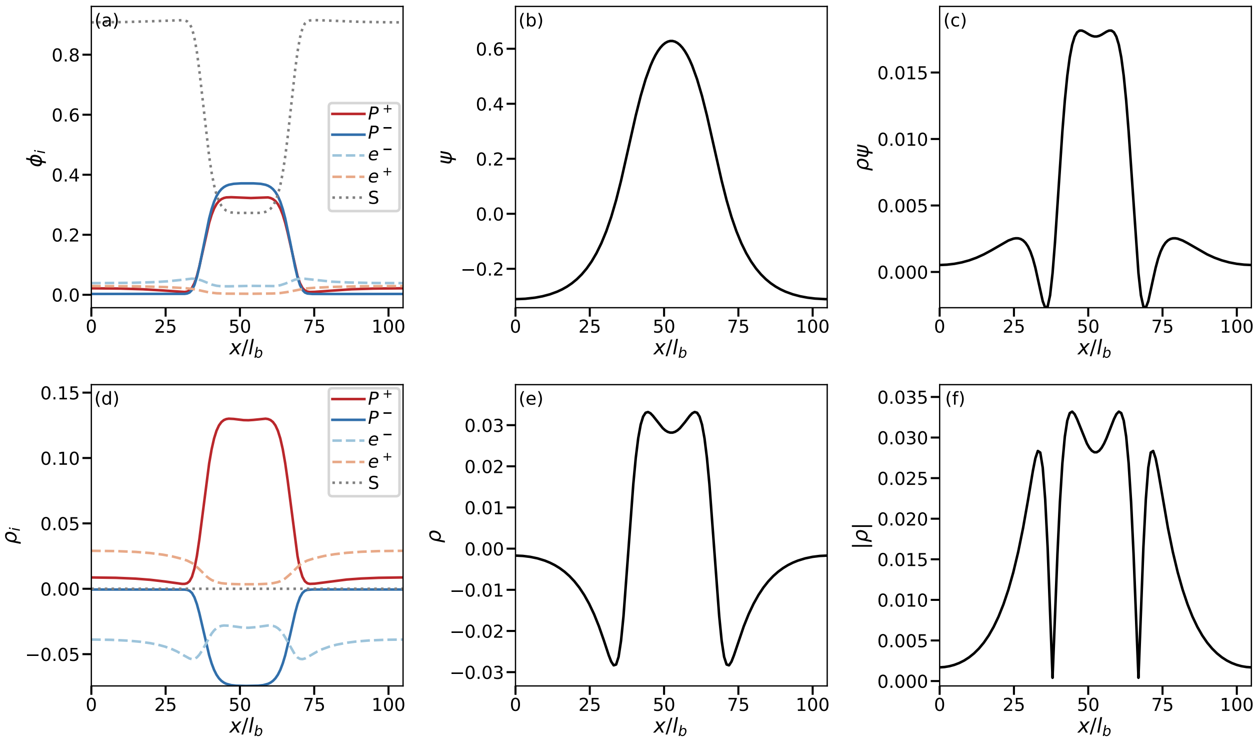

FIG. S38. Additional details for the state for weak charge asymmetry shown in Fig. 2a in the main text. Parameters are $z_{P+} = 0.4$, $z_{P-} = -0.2$, $\chi = -5$, and $\kappa = 10$. (a) Volume fractions ϕ_i as a function of position x. (b) Electrostatic potential $\psi(x)$. (c) Local electrostatic energy density $\rho\psi(x)$. (d) Charge density $\rho_i(x) = z_i\phi_i(x)$ for each species. (e) Total charge density $\rho(x) = \sum_i \rho_i(x)$. (f) Absolute value of total charge density $|\rho(x)|$.

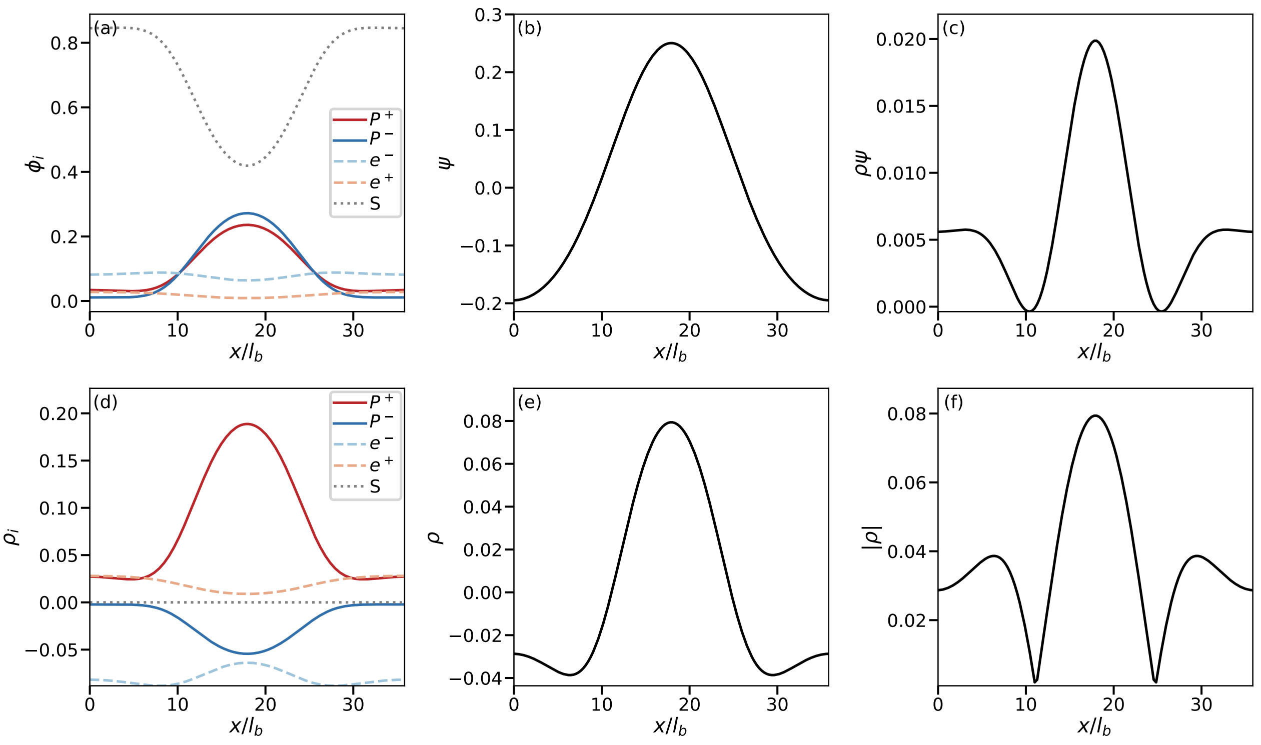

FIG. S39. Additional details for the state for weak charge asymmetry shown in Fig. 2b in the main text. Parameters are $z_{P+} = 0.8$, $z_{P-} = -0.2$, $\chi = -5$, and $\kappa = 10$. (a) Volume fractions ϕ_i as a function of position x. (b) Electrostatic potential $\psi(x)$. (c) Local electrostatic energy density $\rho\psi(x)$. (d) Charge density $\rho_i(x) = z_i\phi_i(x)$ for each species. (e) Total charge density $\rho(x) = \sum_i \rho_i(x)$. (f) Absolute value of total charge density $|\rho(x)|$.

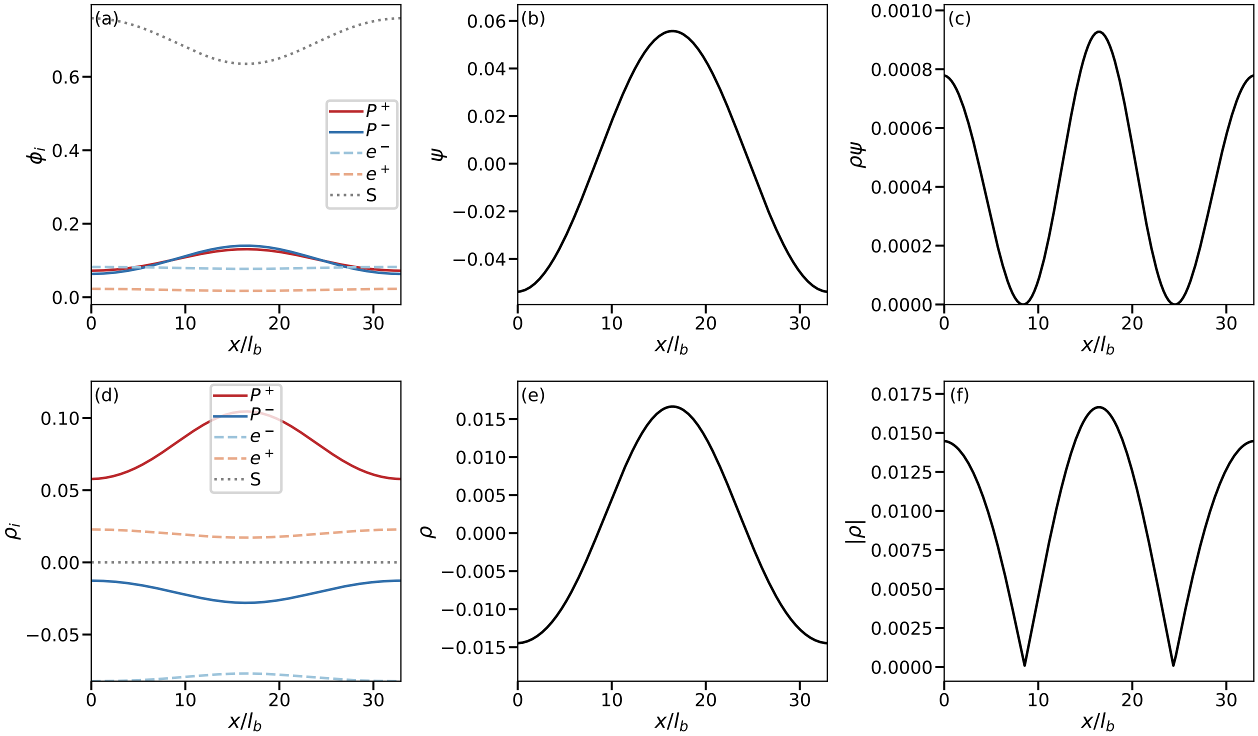

FIG. S40. Typical profile close to continuous transition. Parameters are $z_{P+} = 0.8$, $z_{P-} = -0.2$, $\chi = -4.5$ (the critical point is at $\chi^* \approx -4.46$), and $\kappa = 10$. (a) Volume fractions ϕ_i as a function of position x. (b) Electrostatic potential $\psi(x)$. (c) Local electrostatic energy density $\rho\psi(x)$. (d) Charge density $\rho_i(x) = z_i\phi_i(x)$ for each species. (e) Total charge density $\rho(x) = \sum_i \rho_i(x)$. (f) Absolute value of total charge density $|\rho(x)|$.

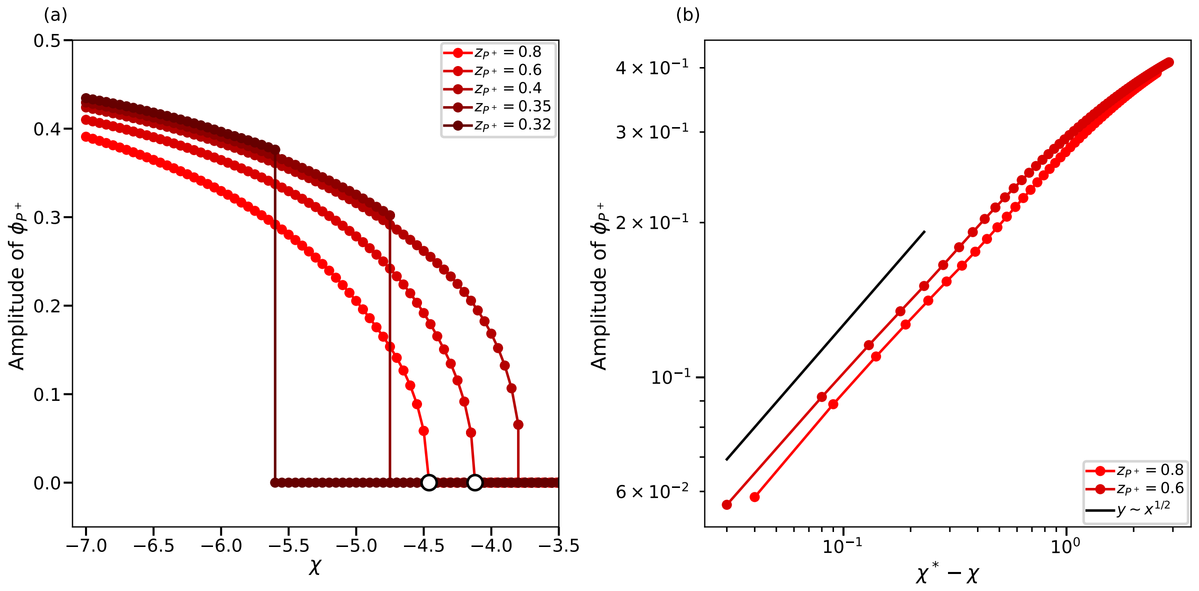

FIG. S41. **Amplitude decreases as χ increases.** (a) The amplitude of fraction ϕ_{P+} of the positively-charged polymer decreases as the interaction parameter χ increases. The circle indicates the critical point at $\chi = \chi^*$ where $\chi^* \approx -4.46$ for $z_{P+} = 0.8$ and $\chi^* \approx -4.12$ for $z_{P+} = 0.6$. (b) Rescaled data of panel a, now shown as a function of the relative interaction strength $\chi^* - \chi$. The exponent $\frac{1}{2}$ is consistent with the critical exponent in mean-field theory, indicating that the transition is continuous. Parameters are $z_{P-} = -0.2$ and $\kappa = 10$. Remaining parameters are the same as in Fig. 2 in the main text.

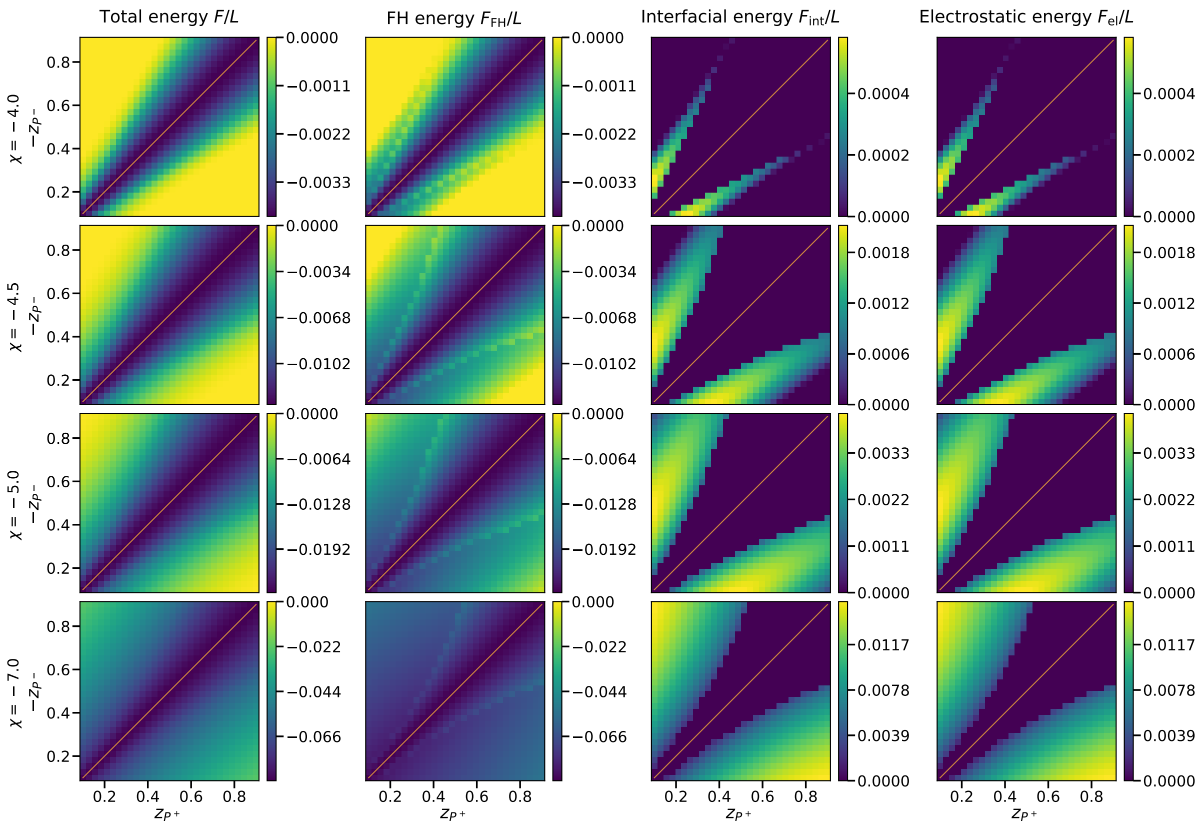

FIG. S42. **Different contributions to the free energy.** Total energy density F, and its three contributions F_{FH}, F_{int}, and F_{el}, normalized to the size L as a function of the charge numbers z_{P+} and z_{P-} of the polymers for various interaction strengths χ (across rows). To highlight the details, F and F_{FH} have been shifted by the free energy $F(\phi_i(x) = \bar{\phi}_i)$ of the respective homogeneous states. Consistent with Eq. (S25), F_{int} and F_{el} are always identical.

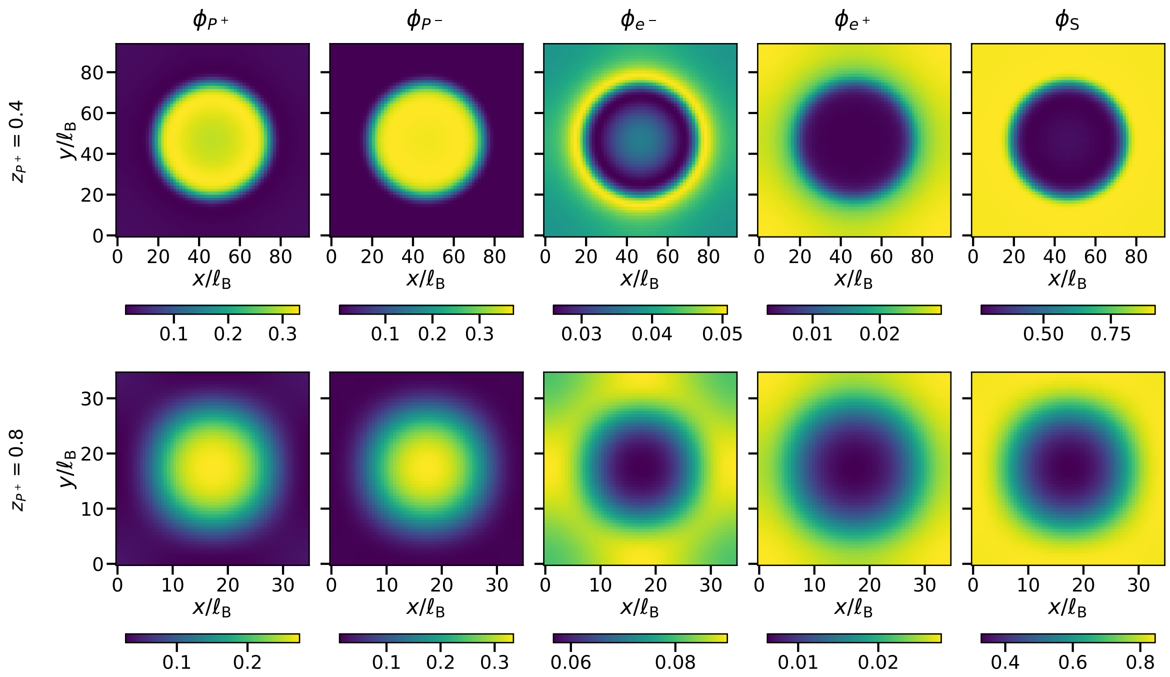

FIG. S43. Two-dimensional profiles the volume fractions $\phi_i(x, y)$ of all species (across columns) predicted by the field theory for two different charge numbers z_{P+} of the positively-charged polymers (across rows). The remaining parameters are the same as in Fig. 2 of the main text.

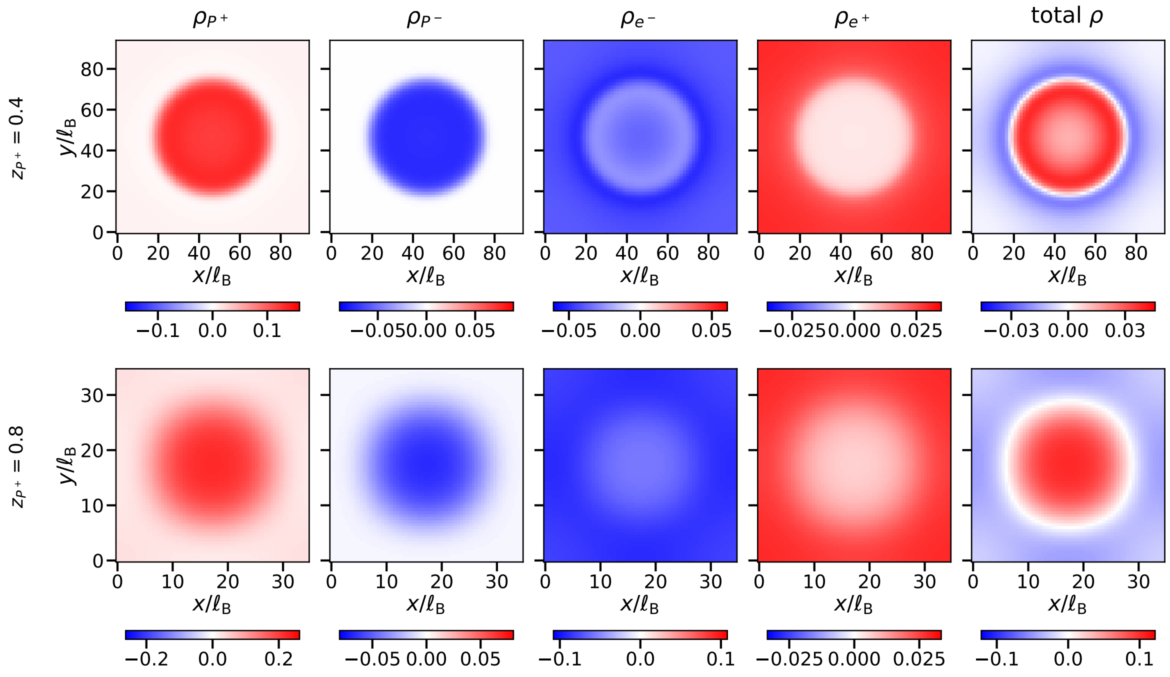

FIG. S44. Two-dimensional profiles of charge densities, $\rho_i(x, y) = z_i\phi_i(x, y)$, of charged species (across columns) predicted by the field theory for two charge numbers z_{P+} of the positively-charged polymers (across rows). Last column shows the total charge density $\sum_i \rho_i(x, y)$.

[1] A. P. Thompson, H. M. Aktulga, R. Berger, D. S. Bolintineanu, W. M. Brown, P. S. Crozier, P. J. in 't Veld, A. Kohlmeyer, S. G. Moore, T. D. Nguyen, R. Shan, M. J. Stevens, J. Tranchida, C. Trott, and S. J. Plimpton, LAMMPS - a flexible simulation tool for particle-based materials modeling at the atomic, meso, and continuum scales, Computer Physics Communications **271**, 108171 (2022).
[2] R. W. Hockney and J. W. Eastwood, *Computer simulation using particles* (crc Press, 2021).
[3] A. Stukowski, Visualization and analysis of atomistic simulation data with OVITO-the Open Visualization Tool, MODELLING AND SIMULATION IN MATERIALS SCIENCE AND ENGINEERING **18**, 10.1088/0965-0393/18/1/015012 (2010).
[4] Y. Qiang, C. Luo, and D. Zwicker, Scaling of phase count in multicomponent liquids, arXiv preprint arXiv:2405.01138 (2024).
[5] L. Q. Chen and J. Shen, Applications of semi-implicit fourier-spectral method to phase field equations, Computer Physics Communications **108**, 147 (1998).
[6] R. B. Thompson, K. O. Rasmussen, and T. Lookman, Improved convergence in block copolymer self-consistent field theory by anderson mixing, The Journal of chemical physics **120**, 31 (2004).
[7] P.-G. de Gennes, Dynamics of fluctuations and spinodal decomposition in polymer blends, The Journal of Chemical Physics **72**, 4756 (1980).
[8] J. Qin, D. Priftis, R. Farina, S. L. Perry, L. Leon, J. Whitmer, K. Hoffmann, M. Tirrell, and J. J. De Pablo, Interfacial tension of polyelectrolyte complex coacervate phases, ACS Macro Letters **3**, 565 (2014).
[9] P. Zhang and Z.-G. Wang, Interfacial structure and tension of polyelectrolyte complex coacervates, Macromolecules **54**, 10994 (2021).
